# Engineering suppressor tRNAs for effective treatment of Duchenne muscular dystrophy

**DOI:** 10.1126/sciadv.aeg3466

**Published:** 2026-09-09

**Authors:** Julien Oury, Ying-Hsin Chen, Sean McFarland, Delilah Jewel, Joel Wen, Teerithveen Pasricha, Steffany Villaseñor, William Mannheim, Victoria Rose, Sophia Zheng, Gokul Sriman Thanigai Arasu, Michael Calhoun, Alexis Dilts, McKenna Montminy, Nathan Strozewski, Nitusingh Thakur, Paul L. Kaplan, Peter M. Eimon, Jeff Coller, Harvey Lodish

**Affiliations:** ^1^ Tevard Biosciences, Lilly Gateway Labs, 15 Necco St., Boston, MA, USA.; ^2^ University of California, Berkeley University, Berkeley, CA, USA.; ^3^ University of California, San Francisco, San Francisco, CA, USA.; ^4^ Stanford University, Stanford, CA, USA.; ^5^ University of Michigan—Ann Arbor, Ann Arbor, MI, USA.; ^6^ University of Pennsylvania, Philadelphia, PA, USA.; ^7^ Johns Hopkins University, RNA Innovation Center, Baltimore, MD, USA.; ^8^ Massachusetts Institute of Technology, Cambridge, MA, USA.; ^9^ Whitehead Institute for Biomedical Research, Cambridge, MA, USA.

## Abstract

Duchenne muscular dystrophy (DMD) is a fatal disorder caused by loss of dystrophin, a protein essential for muscle cell integrity. To date, no therapeutic has restored full-length dystrophin. Suppressor transfer RNAs (sup-tRNAs) have the potential to treat the ∼15% of patients with DMD and nonsense mutations. We have evolved highly potent UAA sup-tRNAs through an extensive mutagenesis screening campaign, optimized our adeno-associated virus (AAV) expression vectors, and developed efficient RNA polymerase III promoters. We show that systemic delivery of these sup-tRNAs restores full-length dystrophin and recovers muscle strength and motor function in mice at lower doses than AAV microdystrophin therapies. We see no adverse toxicology and observe proteome-wide reversion of molecular pathophysiology. These data establish sup-tRNAs as a promising therapeutic platform for DMD and other disorders.

## INTRODUCTION

Duchenne muscular dystrophy (DMD) affects approximately 1 in 5000 boys worldwide and results from loss-of-function mutations in the dystrophin gene. While several therapeutic approaches that slow disease progression have gained approval in the United States, no cure exists for DMD or other muscular dystrophies (*1*). The extraordinary size of the dystrophin gene presents a major obstacle to traditional gene therapy. Located on the X chromosome, the dystrophin gene spans 2.4 million base pairs, contains 79 exons, and encodes 3685 amino acids—making dystrophin one of the largest known human proteins (*2*). Given dystrophin’s size, current dystrophin-restorative therapies rely on either antisense oligonucleotide-based exon skipping to remove mutant exons or viral vector delivery of truncated “microdystrophin” variants (*3*). Microdystrophin therapies deliver shortened dystrophin proteins associated with the milder Becker muscular dystrophy phenotype, while exon skipping approaches are limited to specific exons. These approaches have often resulted in extremely low levels of dystrophin rescue clinically (*4*). To date, no gene therapy has successfully restored full-length dystrophin and concomitantly reversed the physical manifestations of DMD (*5*). Here, we report the development of a tRNA-based gene therapy for the treatment of DMD that restores full-length protein, ameliorates disease pathophysiology, and improves motor function.

Nonsense mutations, in which coding triplets are converted to premature stop codons, are estimated to underlie 11 to 20% of all inherited genetic diseases (*6*), with a prevalence of more than 40% in some disorders (*7*). These mutations represent the leading cause of genetic disease at the molecular level. Living organisms typically express approximately 50 distinct tRNA isoacceptors that collectively decode the genetic code (*8*). Each tRNA features an anticodon sequence that base pairs with corresponding mRNA codons. Groundbreaking studies in the 1960s revealed that certain bacterial and fungal tRNAs harbor anticodon mutations enabling recognition of stop codons (*9*). These “suppressor tRNAs” earned their name through their capacity to rescue nonsense mutations by facilitating ribosomal readthrough of premature termination signals (*10*). Given their direct mechanism of action—decoding disease-causing stop codons as sense codons—sup-tRNAs represent a promising yet underdeveloped therapeutic strategy for patients with nonsense mutation-related genetic disorders (*9*).

In this work, we demonstrate that the in vivo readthrough efficiency of sup-tRNAs derived from endogenous tRNAs can be substantially enhanced by strategic nucleotide changes outside of the anticodon. Using a combination of in vitro and in vivo screening modalities, we evolved sup-tRNAs capable of reading through the highly stringent UAA nonsense mutation in a mouse model of DMD to restore full-length dystrophin protein and recover lost motor function. By combining potent evolved sup-tRNAs with an optimized adeno-associated virus (AAV) delivery vector and a highly efficient RNA polymerase III (Pol III) promoter, we achieved nearly complete recovery of skeletal muscle function across the board at a systemic dose of 1 × 10^14^ vg/kg and at levels of viral transduction substantially less than reported clinically for microdystrophin therapies. Systemic delivery resulted in dose-dependent dystrophin protein rescue, restoration of motor function, and reversal of molecular pathophysiology. The rescued dystrophin protein was correctly localized to the plasma membrane and detectable across all affected tissues analyzed. Sup-tRNAs were well tolerated and did not induce a detectable increase in the background level of native stop codon readthrough. These findings establish sup-tRNAs as a promising therapeutic strategy for patients with DMD and nonsense mutations.

## RESULTS

### Non-optimized parental sup-tRNAs fail to rescue DMD muscle deficits in vivo

The predominant strategy for developing suppressor tRNAs involves modifying natural tRNAs to decode premature termination codons through targeted nucleotide substitutions in their anticodons. We began by evaluating 18 UUA sup-tRNAs—in which a UUA anticodon pairs with a UAA nonsense mutation—derived from endogenous human and mouse Gln-tRNAs (tRNA^Gln^UUA sup-tRNAs). All sup-tRNAs were generated via exchange of the anticodon triplet without additional nucleotide changes (table S1). We term such sup-tRNAs, which lack sequence alterations outside of the anticodon, “parental sup-tRNAs.” Sup-tRNA activity was first quantified in vitro in human embryonic kidney (HEK) 293 cells via transient transfection. To quantify readthrough activity, we used a dual luciferase reporter assay consisting of an intact N-terminal firefly luciferase fused to a C-terminal Nanoluciferase (NanoLuc) luciferase; expression of the latter was prevented by an inactivating glutamine-to-UAA nonsense mutation (Q586*). Suppressor sequences were synthesized as double-stranded DNA fragments incorporating a downstream RNA Pol III termination signal and coupled to an upstream human U6 promoter to enhance expression compared to endogenous tRNA promoters (*10*, *11*). At 24 hours posttransfection, suppressor activity was determined by the NanoLuc to firefly luciferase ratio. Activity was normalized to a benchmark tRNA^Gln^UUA parental sup-tRNA (tr0157) that contained a mutation only in the anticodon and was included as an internal control on all plates screened. Six “parental” sup-tRNAs were chosen for further in vivo validation based on in vitro activity (fig. S1A) and overall sequence diversity from one another (fig. S1B).

We next tested parental sup-tRNAs for in vivo efficacy in a mouse model of DMD. D2.mdx mice harbor an inactivating nonsense mutation in dystrophin exon 23, creating a UAA termination codon in place of a glutamine codon. Of the three stop codons, UAA has the highest termination fidelity and has proven difficult to address with sup-tRNAs in previous studies (*11*, *12*). The DBA/2J (D2) genetic background produces more severe skeletal muscle pathology, enhanced inflammation, and accelerated cardiac dysfunction compared to the standard B10-*mdx* strain. This combination of a severe, clinically relevant mouse model and a challenging nonsense mutation provides a stringent platform to evaluate therapeutic sup-tRNAs (*13*).

In addition to the six tRNA^Gln^UUA sup-tRNAs selected on the basis of in vitro testing, we evaluated UUA sup-tRNAs derived from two Ser-tRNAs (tRNA^Ser^UUA) and two Tyr-tRNAs (tRNA^Tyr^UUA) in the D2.mdx mouse model (table S1). Like the Gln-tRNAs, these were parental sup-tRNAs in which nucleotide changes were limited to the anticodon. To achieve robust sup-tRNA expression in mice, we constructed AAV vectors containing three tandem copies of the tRNA. Each copy incorporated a downstream RNA Pol III termination signal and was coupled to an upstream human U6 promoter to enhance expression compared to endogenous tRNA promoters (fig. S2) (*10*). These vectors were packaged into MyoAAV2A, an AAV serotype optimized for myotropic delivery (*14*, *15*). AAVs expressing sup-tRNAs were initially administered via intramuscular (IM) injection into tibialis anterior (TA) muscle of 12-week-old D2.mdx hemizygous male mice, allowing for rapid in vivo screening requiring minimal amounts of AAV. Dystrophin protein levels were measured at 6 weeks posttreatment by capillary Western blot (*16*). Full-length dystrophin protein was detected in untreated wild-type mice and in all cohorts of D2.mdx mice dosed with sup-tRNAs, but not in untreated D2.mdx mice. Rescue of full-length dystrophin varied by sup-tRNA, reaching a peak of 15.0 ± 5.9% of normal in mice dosed with the parental sup-tRNA designated tr0524 (fig. S2).

In patients, DMD pathology is present in all skeletal muscles as well as cardiac muscle, with mortality typically due to either respiratory or heart failure (*17*). We therefore sought to determine whether systemic delivery of tr0524—the best parental sup-tRNA identified by IM screening—would be capable of restoring sufficient full-length dystrophin to ameliorate motor function deficits in D2.mdx mice. MyoAAV2A expressing sup-tRNA tr0524 (AAV-tr0524) was injected intravenously (IV; tail vein) into 12-week-old mice using escalating doses of 1, 2, or 4 × 10^14^ vg/kg. These doses were chosen to align with doses used for DMD microdystrophin gene therapies in the clinic. Specifically, Elevidys (Sarepta Therapeutics) is dosed at 1.33 × 10^14^ vg/kg, while SGT-003 (Solid Biosciences) and RGX-202 (REGENXBIO) have been tested in clinical trials at 1 and 2 × 10^14^ vg/kg. We therefore selected our two lower doses to match existing DMD gene therapies while including a third higher dose to explore the efficacy and toxicity of sup-tRNAs in vivo. Muscle strength and motor coordination were assessed using grip strength and Rotarod assays (*18*) at 11 weeks posttreatment. Untreated D2.mdx mice exhibited profound deficits in both assays when compared with wild-type controls. Systemic delivery of AAV-tr0524 failed to significantly improve muscle function at all of the doses tested (fig. S3). The failure of even the most efficient UUA sup-tRNA identified by IM screening suggested to us that parental sup-tRNAs may generally lack sufficient potency to effect meaningful improvement in a challenging disease model such as DMD—at least when used to target UAA nonsense mutations.

### Evolution of engineered sup-tRNAs with increased activity in vivo

Optimization of suppressor tRNAs via sequence changes outside of the anticodon has been shown to augment readthrough activity in specific contexts (*19*). We therefore screened a library of tRNAs (table S1) for more efficient UAA suppressors using the same dual luciferase reporter assay used previously. The bulk of this library comprised “engineered” tRNA^Gln^UUA sup-tRNAs containing one or more nucleotide substitutions outside of the anticodon in addition to the anticodon exchange required to target UAA nonsense mutations. All engineered sup-tRNAs in the library were within four nucleotide changes of one or more parental sup-tRNAs (edit distance ≤4). Nucleotide substitutions were explored in the following regions: acceptor stem (altered in 39% of engineered tRNAs), D-arm (altered in 19% of engineered tRNAs), anticodon arm (excluding the anticodon itself; altered in 29% of engineered tRNAs), the T-arm (altered in 23% of engineered tRNAs), and positions outside of these four domains, including the variable loop (altered in 33% engineered tRNAs). A number of engineered tRNAs contained substitutions in more than one region. In addition to UAA suppressors, the library contained several controls not anticipated to suppress UAA stop codons: (i) wild-type Gln-TTG tRNAs (*n* = 7), (ii) wild-type Gln-CTG tRNAs (*n* = 11), and (iii) tRNA^Gln^CUA sup-tRNAs with anticodons targeting UAG stop codons (*n* = 26). As expected, negative controls showed virtually no readthrough activity when screened in the dual luciferase assay (Fig. 1A; controls indicated in blue at the left of the plot). The mean luciferase score for parental suppressors without additional changes was 0.94 ± 0.26 (fig. S1A; five low-performing parental sup-tRNAs were excluded when calculating mean) with the best parental reaching 1.12 ± 0.33 (tr0520). Among the 309 engineered sup-tRNAs included in the library, 128 achieved luciferase reporter readthrough equal to or better than the parental sup-tRNA mean. The top tRNA^Gln^UUA sup-tRNA identified, tr1626, contained four unique nucleotide changes relative to the most closely related parental suppressor (tr0520) and had a dual luciferase activity score of 1.75 ± 0.30. While the precise mechanistic basis for enhanced suppression activity remains to be fully elucidated, nucleotide changes outside the anticodon may influence multiple steps in tRNA function, including aminoacylation efficiency by glutaminyl-tRNA synthetase, binding affinity to elongation factor EF-1A, stability of codon-anticodon interactions in the ribosomal decoding center, or resistance to quality control mechanisms that normally discriminate against stop codon recognition.

**Fig. 1.**
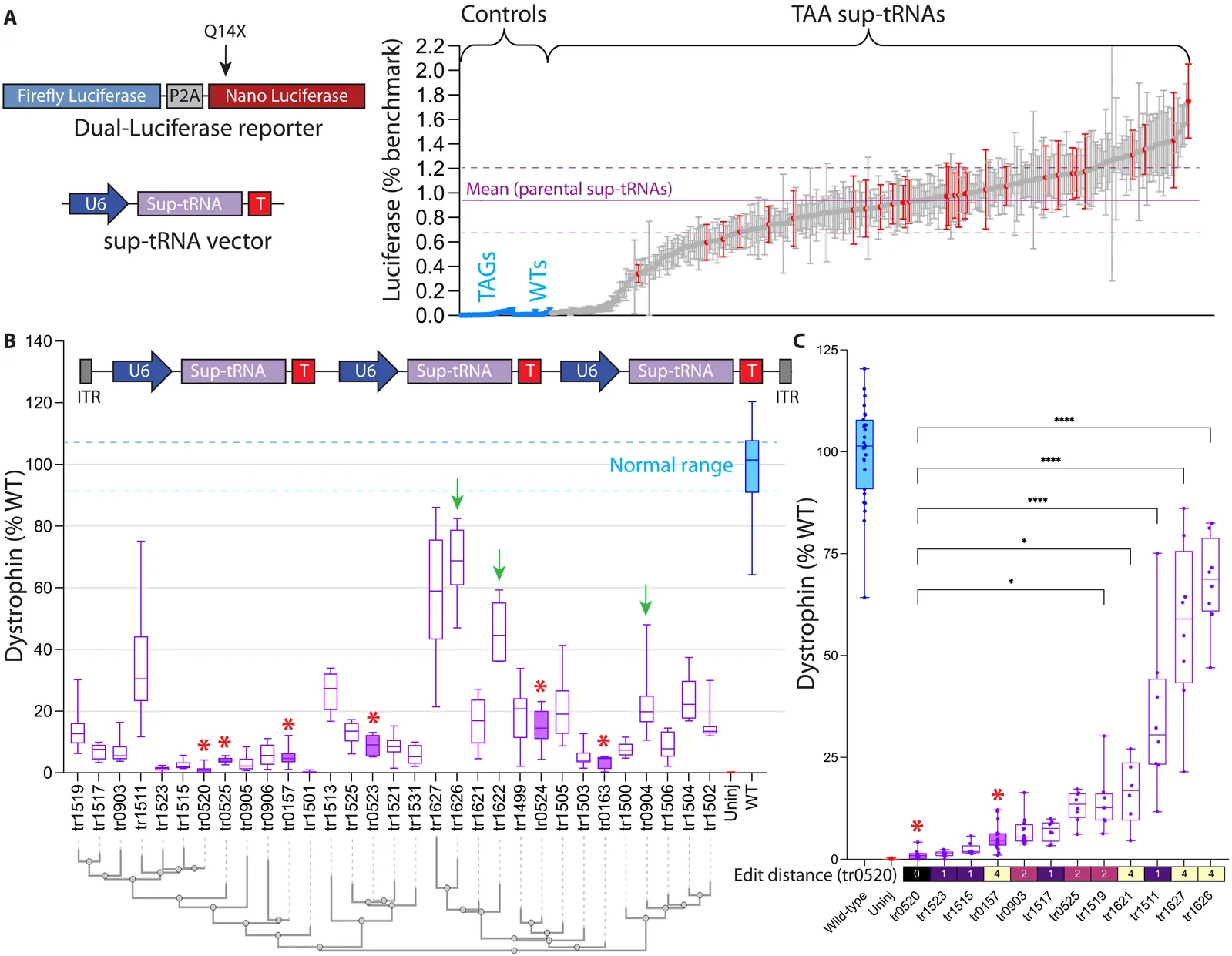
Evolution of parental sup-tRNAs to enhance in vivo activity. (**A**) Diagram of dual luciferase assay used to screen tRNA^Gln^UUA sup-tRNAs in vitro in HEK293 cells and results at 24 hours posttransfection from 309 candidates targeting UAA nonsense mutations (gray) and 44 nontargeting controls (blue). Data points in red were selected for further testing in vivo. (**B**) Schematic diagram of sup-tRNA AAV expression cassette and quantification of dystrophin expression in TA muscles following IM injection of 1 × 10^11^ vg per muscle of AAVs expressing indicated sup-tRNAs. Sup-tRNAs are clustered by sequence similarity. (**C**) Dystrophin rescue in TA muscles for all tRNAs with an edit distance of ≤4 from parental sup-tRNA tr0520. Red asterisks and purple shading, parental sup-tRNAs; green arrows, sup-tRNAs selected for additional testing via systemic dosing; U6, human U6 promoter; T, RNA Pol III termination signal; P2A, P2A self-cleaving peptide; ITR, inverted terminal repeat; TAGs, tRNA^Gln^CUA sup-tRNAs; WTs, wild-type Gln-tRNAs. *P < 0.05, ****P < 0.00005.

We chose 25 engineered tRNA^Gln^UUA suppressors for preliminary in vivo validation in the D2.mdx model via IM delivery. Rather than focusing exclusively on the top hits from in vitro screening, we selected engineered sup-tRNAs spanning a wide range of luciferase activities to better understand the robustness of in vitro versus in vivo correlations (Fig. 1A; data points in red indicate sup-tRNAs that were selected for IM injection). The chosen sup-tRNAs represented a diverse candidate pool as illustrated by sequence similarity clustering and edit distances relative to parental sup-tRNAs (fig. S4). As with parental sup-tRNAs, evolved sup-tRNAs were delivered via MyoAAV2A and expressed from a single-stranded AAV (ssAAV) vector containing three copies of the tRNA, each paired with a U6 promoter. D2.mdx mice were dosed by IM injection, and dystrophin levels were measured at 6 weeks posttreatment. Rescue of full-length dystrophin varied by sup-tRNA (Fig. 1B). Eleven of 25 engineered sup-tRNAs performed better than tr0524—the best parental sup-tRNA. The most efficient of these—tr1626—rescued full-length dystrophin to 67.9 ± 11.6% of levels in wild-type mice. This represents a ∼4.5-fold improvement in in vivo suppression activity relative to tr0524, the best parental sup-tRNA. The most closely related parental sup-tRNA to tr1626 is tr0520 (fig. S4; edit distance = 4). The four nucleotide changes that separate tr0520 from tr1626 were sufficient to produce a >60-fold increase in dystrophin rescue in vivo (1.1% versus 67.9% of normal level). A side-by-side comparison of all sup-tRNAs tested in D2.mdx mice with an edit distance of ≤4 from parental sup-tRNA tr0520 shows that even a small number of edits outside of the anticodon can positively modulate in vivo suppression efficiency by well over an order of magnitude (Fig. 1C). Notably, tr1626 was also the top-performing sup-tRNA identified by in vitro screening (Fig. 1A). In general, the best- and worst-performing sup-tRNAs identified via in vitro screening behaved consistently in vivo with a significant (*P* < 0.0001) coefficient of determination of 0.14 (fig. S5). The observed variation in screening results may be attributable to differences in assay duration (24 hours for HEK cells; 6 weeks for mice) and/or differences in sup-tRNA performance when delivered via transfection versus AAV transduction. Analysis of AAV transduction confirmed that—despite animal-to-animal variability in biodistribution following IM dosing—increased dystrophin rescue by engineered sup-tRNAs relative to AAV-tr0524 was due to properties intrinsic to the newly engineered sequences rather than differences in TA muscle infection (fig. S6).

### Systemic administration of evolved sup-tRNAs is well tolerated and restores dystrophin protein in all impacted tissues

We selected tr0904—an engineered sup-tRNA with mid-range efficiency in IM screening (21.5 ± 8.3% dystrophin rescue)—for in depth characterization following systemic delivery. Two additional high-performing evolved sup-tRNAs (tr1622 and tr1626) were also set aside for systemic dosing following a parallel AAV vector optimization effort described below. To assess the long-term durability of dystrophin rescue, we dosed a cohort of D2.mdx mice with MyoAAV2A expressing sup-tRNA tr0904 (AAV-tr0904) and analyzed TA muscles at 24 weeks posttreatment. No differences were observed in AAV transduction (Fig. 2A), tr0904 levels (Fig. 2B), or rescued dystrophin protein (Fig. 2C) in animals at 24 weeks postinjection relative to animals at 6 weeks postinjection.

**Fig. 2.**
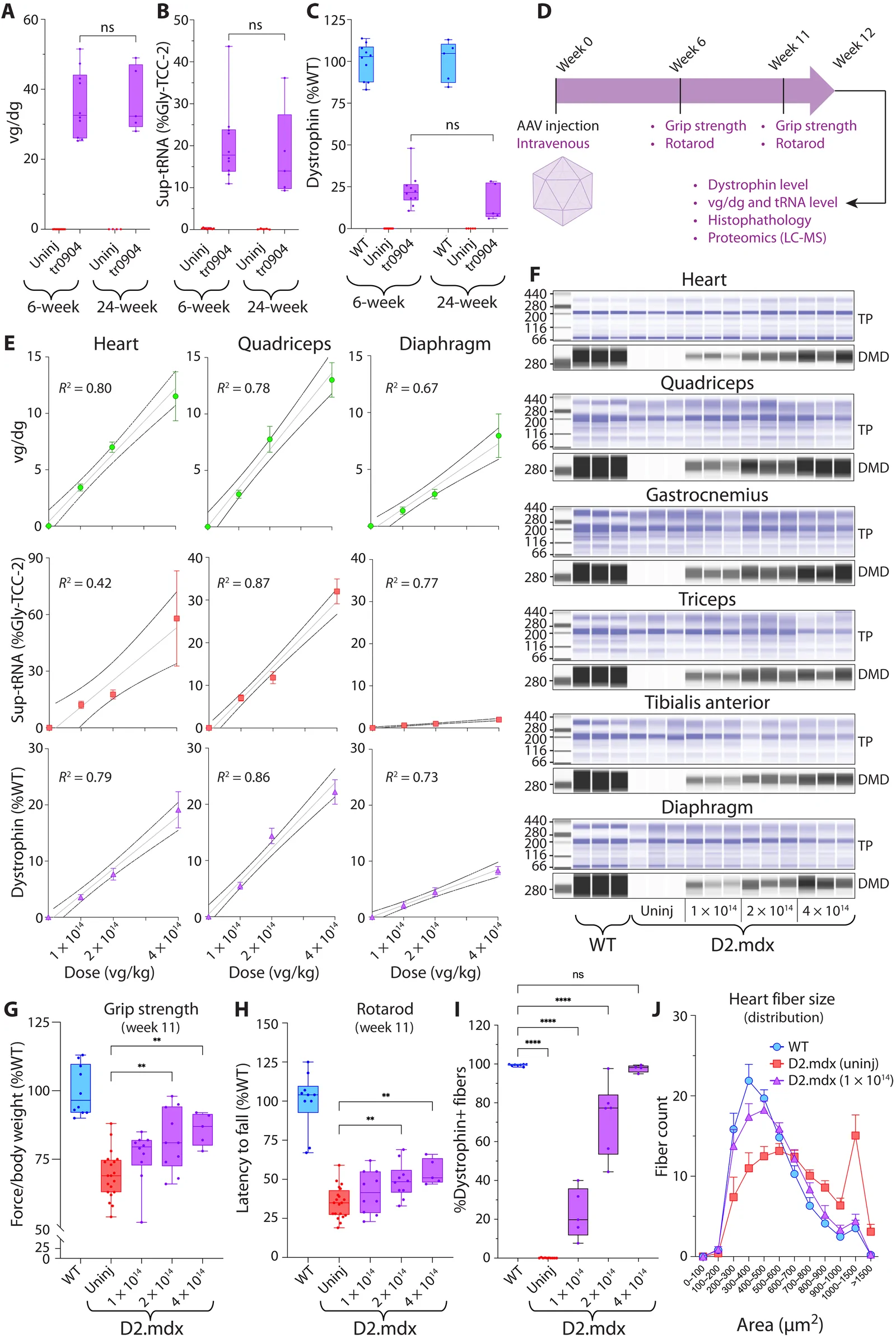
AAV-tr0904 restores full-length dystrophin protein and improves motor function in D2.mdx mice. (**A**) Quantification of AAV transduction, (**B**) sup-tRNA expression, and (**C**) dystrophin expression in TA muscles at 6 weeks and 24 weeks after IM injection of AAV-tr0904. AAV transduction results expressed as vector genomes per diploid genome (vg/dg). Sup-tRNA was measured by RT-ddPCR and normalized to endogenous tRNA-Gly-TCC-2. Full-length dystrophin was measured by capillary Western blot. Plots show dystrophin levels in individual animals normalized to wild-type average. WT, uninjected wild-type controls; Uninj, uninjected D2.mdx controls; tr0904, D2.mdx mice injected IM with AAV-tr0904 at 1 × 10^11^ vg per muscle. (**D**) Experimental design for systemic rescue of dystrophin. AAV-tr0904 was injected IV into 12-week-old D2.mdx mice at 1, 2, or 4 × 10^14^ vg/kg. Motor function was assessed at 6 and 11 weeks postinjection using grip strength and Rotarod assays. Tissues were collected at 12 weeks posttreatment. (**E**) Quantification of AAV transduction, sup-tRNA expression, and dystrophin expression in heart, quadriceps muscle, and diaphragm. Dystrophin levels in individual animals were normalized to wild-type average. Plots show mean, SEM, and 95% confidence intervals for each dose. (**F**) Representative capillary Western blots (three animals per cohort) of total protein (TP; blue) and full-length dystrophin (DMD; black) in indicated tissues from controls and AAV-tr0904–treated D2.mdx mice. Mice were tested for grip strength (**G**) and average latency to fall from a rotating Rotarod (**H**) at 11 weeks posttreatment. (**I**) Quantification of dystrophin positive muscle fibers in heart muscle. (**J**) Muscle fiber size distribution (in square micrometers) in heart muscle from controls and tr0904-treated mice (1 × 10^14^ vg/kg). (G, H, and I) Box plots with individual data points showing the middle line as the median; upper and lower quartiles as box limits; whiskers go down to the smallest value and up to the largest. **P < 0.005 and ****P < 0.00005. ns, not significant.

AAV-tr0904 was injected IV into 12-week-old D2.mdx mice using escalating doses of 1, 2, or 4 × 10^14^ vg/kg. Twelve weeks after treatment, tissue was collected from skeletal and cardiac muscles for in-depth analysis (Fig. 2D). Systemic administration of AAV-tr0904 was well tolerated and resulted in a dose-dependent increase in transduction in skeletal and cardiac muscle (Fig. 2E, top row, and fig. S7A). Expression of sup-tRNA tr0904 was highly correlated with AAV transduction (Fig. 2E, middle row, and fig. S7, B and C). Treated D2.mdx mice showed normal weight gain and no mortality over the course of the 12-week study (fig. S8). No elevation was observed in serum markers of hepatic, muscular, or renal toxicity (fig. S9). Levels of tr0904 did not surpass endogenous tRNA-Gly-TCC-2 (fig. S7B) or endogenous tRNA-Gln-CTG-3-1 (fig. S7C) in the tissues examined. This shows that systemic AAV delivery does not lead to supraphysiological expression of the sup-tRNA, which might result in competition for glutaminyl-tRNA synthetase or other proteins and ribonucleoprotein complexes involved in tRNA biogenesis. AAV-tr0904 treatment produced widespread rescue of full-length dystrophin as measured by Western blot [Fig. 2, E (bottom row) and F, and fig. S10]. Levels of dystrophin were significantly increased relative to untreated D2.mdx controls in heart, gastrocnemius, quadriceps, diaphragm, TA, and triceps, reaching a high of 22 ± 2% of normal levels in quadriceps muscle at 4 × 10^14^ vg/kg. Even at the highest dose, AAV-tr0904 was well tolerated and continued to show dose-dependent improvement.

In contrast to parental sup-tRNAs, AAV-tr0904 achieved significant functional recovery in mutant mice. Muscle strength and motor coordination were assessed using grip strength and Rotarod assays (*18*) at 6 and 11 weeks posttreatment. Untreated D2.mdx mice exhibited profound deficits in all-limb grip strength compared with wild-type controls at both time points. AAV-tr0904 produced dose-dependent improvements at 11 weeks (but not at 6 weeks), reaching 86.0 ± 6% of normal grip strength (Fig. 2G). The Rotarod assay was used to assess motor coordination and balance. Severe motor deficits in D2.mdx mice were substantially improved by AAV-tr0904 treatment, with treated cohorts showing significant dose-dependent improvements in latency to fall at the two highest doses (Fig. 2H). These functional outcomes demonstrate that even a mid-range engineered sup-tRNA—in contrast to all parental sup-tRNAs tested—is capable of restoring full-length dystrophin protein in vivo and translating this molecular correction into meaningful improvements in muscle strength and motor function.

We used a mass spectrometry (MS)–based proteogenomic approach to further assessing the ability of engineered sup-tRNAs to read through nonsense mutations in dystrophic mice. Liquid chromatography–tandem mass spectrometry (LC-MS/MS) global proteomic analysis was performed on quadriceps muscles and analyzed using data-independent acquisition (DIA). This approach quantified 3714 protein groups across all samples, providing comprehensive coverage for subsequent comparative proteomics analysis. Sup-tRNA–mediated readthrough of the dystrophin nonsense mutation was readily apparent in quadriceps muscle (fig. S11A). Consistent with capillary Western blot assays, AAV-tr0904 treatment resulted in a dose-dependent increase in both the total number of tryptic peptides that were detected downstream of the nonsense mutation and in the signal intensity of the rescued peptides (fig. S11, B and C).

### Engineered sup-tRNAs show high selectivity for nonsense mutations over native stop codons

A potential concern with sup-tRNA therapy is whether these molecules might inappropriately read through native stop codons in addition to their intended nonsense mutation targets. To assess whether AAV-tr0904 induces off-target readthrough of native stop codons in vivo at therapeutically efficacious doses, we constructed a customized proteomic database containing sequences resulting from UAA-to-glutamine substitutions at native stop codons. This database included 5356 open reading frames terminating in UAA, with sequences extended until the next in-frame stop codon.

Approximately 26.4% (5356 of 20,253) of protein sequences in the mouse proteome reference database use a native UAA stop codon that could potentially be subject to off-target readthrough by AAV-tr0904. Among the 3714 protein groups detected in quadriceps muscles, off-target LC-MS/MS analysis identified only five 3′ untranslated region (3′UTR) extended peptides mapping to four unique proteins in AAV-tr0904–treated mice (fig. S12 and table S2). No significant change in expression relative to untreated D2.mdx mice was observed in any of the four proteins (fig. S13). Notably, all four proteins with detectable readthrough were among the most highly expressed proteins in muscle tissue, present at levels >2 × 10^3^ above the detection limit established by the least abundant identified proteins (fig. S14). Two separate 3′UTR peptides associated with Titin (Ttn) were detected, one of which (GPFTLDAHS) was also detected in a single untreated D2.mdx mouse. This suggests that the native UAA stop in Ttn may undergo detectable levels of readthrough even in the absence of sup-tRNA treatment (fig. S12 and table S2). The absence of detectable 3′UTR peptides from lower-abundance proteins demonstrates that native stop codon readthrough, when it occurs, affects a minimal fraction of translation events. This was further confirmed by comparing signal intensities of the five detected 3′UTR extended peptides to intensities of the top five tryptic peptides from the coding region of each associated protein (fig. S15, A to D). In all cases, peptides upstream of the native UAA stop codon were 10^2^ to 10^3^ times more intense than the associated readthrough peptides, even at the highest dose of AAV-tr0904 tested. In contrast to the minimal number and intensity of detected 3′UTR extended peptides, AAV-tr0904 treatment substantially increased multiple tryptic peptides from dystrophin in D2.mdx mice (fig. S11). This on-target effect of sup-tRNA treatment was readily apparent in LC-MS/MS analysis despite dystrophin protein being far less abundant overall in quadriceps muscle than the four proteins with detected 3′UTR readthrough (fig. S15, E and F). To further contextualize our observations in sup-tRNA–treated mice, studies estimate that natural readthrough due to ribosomal error occurs at native mammalian stop codons at frequencies of 0.01 to 0.1%, indicating that sup-tRNA treatment does not substantially increase background readthrough above endogenous levels (*20*).

### Engineered sup-tRNAs rescue the DMD molecular pathophysiology

The molecular pathophysiology underlying DMD has been extensively characterized by proteomic profiling, and disease-specific protein signatures are well documented in both patients and animal models (*21*, *22*). We analyzed LC-MS/MS results from quadriceps muscles to determine whether AAV-tr0904 treatment reverses these dystrophic signatures. To quantify disease-associated proteomic changes, we performed *t* tests comparing wild-type mice to all D2.mdx cohorts with the Benjamini-Hochberg procedure for controlling FDR. Untreated D2.mdx mice exhibited 449 differentially expressed proteins (>2-fold change, adjusted *P* < 0.05) relative to wild-type controls. AAV-tr0904 treatment produced dose-dependent normalization, reducing dysregulated proteins to 310 (1 × 10^14^ vg/kg), 222 (2 × 10^14^ vg/kg), and 217 (4 × 10^14^ vg/kg) (fig. S16).

One-way analysis of variance (ANOVA) identified proteins whose expression was significantly altered by sup-tRNA treatment compared to untreated D2.mdx mice (adjusted *P* < 0.05). Heatmap visualization revealed progressive reversion toward wild-type expression profiles, with the most pronounced normalization observed at the two highest doses of AAV-tr0904 (fig. S17). Among the proteins whose expression was normalized by AAV-tr0904 were many with well-established connections to the underlying pathology of DMD (figs. S18 to S21 and supplementary discussion). These proteomic analyses demonstrate that dystrophin rescue via sup-tRNAs substantially reduces DMD-associated molecular pathology, with proteome changes primarily reflecting restoration of normal protein expression patterns rather than off-target changes.

### Engineered sup-tRNAs rescue muscle pathology in dystrophic mice

Tissue sections from AAV-tr0904–treated mice were analyzed by immunohistochemistry to measure the number of dystrophin-positive muscle fibers and the subcellular localization of the rescued protein. The detection of a small number of positive fibers in mutant mice was likely due to the presence of sporadic dystrophin-positive muscle fibers, called revertant fibers, which are observed in otherwise dystrophin-negative backgrounds in patients with DMD and animal models (*23*). In agreement with Western blot and LC-MS/MS results, significantly more dystrophin-positive fibers were detected in cardiac muscle (Fig. 2I and fig. S22A) and TA muscle (fig. S22B) from mice treated with AAV-tr0904. Dystrophin has the critical role of stabilizing the muscle membrane (sarcolemma) during muscle contraction by binding to the intracellular actin network, linking the cytoskeleton to the dystrophin-associated glycoprotein complex. This network, in turn, connects to the basal lamina by interacting with extracellular matrix ligands. The colocalization of dystrophin and laminin proteins in heart and TA muscle confirms that rescued dystrophin is at the membrane of treated muscle fibers, likely allowing for the assembly of the muscle stabilization complexes (*24*, *25*).

Abnormalities in muscle fiber size are a hallmark of dystrophic muscle (*18*, *26*). This pathology manifests as greater cell diameter in the heart, consistent with the cardiomyopathy phenotype reported in D2.mdx mice (*27*). In contrast, most skeletal muscles of D2.mdx mice—including TA—exhibit abnormally small fibers indicative of muscle atrophy. Systemic administration of AAV-tr0904 in *mdx* mice led to normalization of muscle fiber area in both cardiac muscle (Fig. 2J and fig. S22A, right) and TA muscle (fig. S22B, right). We observed nearly complete rescue of cardiac muscle fiber size abnormality even at the lowest dose tested (1 × 10^14^ vg/kg; fig. S23A), while rescue of TA muscle atrophy occurred in a dose-dependent manner (fig. S23B). Partial normalization of muscle fiber size pathology was also observed in several other skeletal muscles examined (fig. S23).

The presence of central nuclei in myofibers is a well-established indicator that myofiber damage and repair have occurred (*28*). We therefore measured the number of myofibers with central nuclei as a surrogate for muscle fiber health. Higher levels of central nuclei were evident in D2.mdx mutant mice, and the number of fibers presenting central nuclei significantly decreased starting at 2 × 10^14^ vg/kg, suggesting improved muscle fiber health after treatment with AAV-tr0904 (fig. S24).

### Optimizing AAV-delivered sup-tRNAs to achieve functional efficacy at reduced dose

Although systemic AAV gene therapies have been used successfully to treat a variety of diseases, fatalities have been observed at high doses. In 2025, two patients with DMD died due to liver failure following treatment with Elevidys (1.33 × 10^14^ vg/kg). At least one other high-dose microdystrophin therapeutic—PF-06939926 (Pfizer; 2 × 10^14^ vg/kg)—was discontinued due to clinical trial participant deaths (*29*, *30*). We therefore explored multiple parallel strategies to further increase expression and activity of engineered sup-tRNAs with the goal of achieving functional efficacy in the range of 10^13^ vg/kg.

Numerous studies have reported that self-complementary AAVs (scAAVs) result in increased expression of transgenes in vivo, including in the context of noncoding RNAs expressed from RNA Pol III promoters in skeletal muscle (*31*). We therefore compared ssAAV vectors containing one, two, or three copies of a top-performing evolved sup-tRNA (tr1622) with a scAAV vector containing only a single copy of the same sup-tRNA. As in our previous studies, all sup-tRNAs were paired with U6 promoters and delivered via IM injection. Significantly higher levels of dystrophin protein were detected in mice treated with the single-copy scAAV relative to the single-copy ssAAV (Fig. 3A; 42.4 ± 16.5% versus 14.2 ± 5.6%, respectively). The increase in efficiency achieved with the scAAV was sufficient for a single copy of the sup-tRNA to rescue as much dystrophin as three copies of the same suppressor on a ssAAV. These data show that high readthrough efficiency can be achieved without the use of multimeric AAV vector designs, which have been shown to lead to truncated vector genomes arising from sequence repeats in previous sup-tRNA publications (*10*). In addition to resulting in reduced expression of sup-tRNAs, truncated genomes can affect production efficiency and result in heterogeneity across vector populations, raising regulatory and safety concerns. We detect two distinct truncated vector genomes—in addition to the expected full-length genome—when running three-copy ssAAV vectors on a denaturing gel but only a single, intact, full-length genome from the equally potent single-copy scAAV (Fig. 3, B and C).

**Fig. 3.**
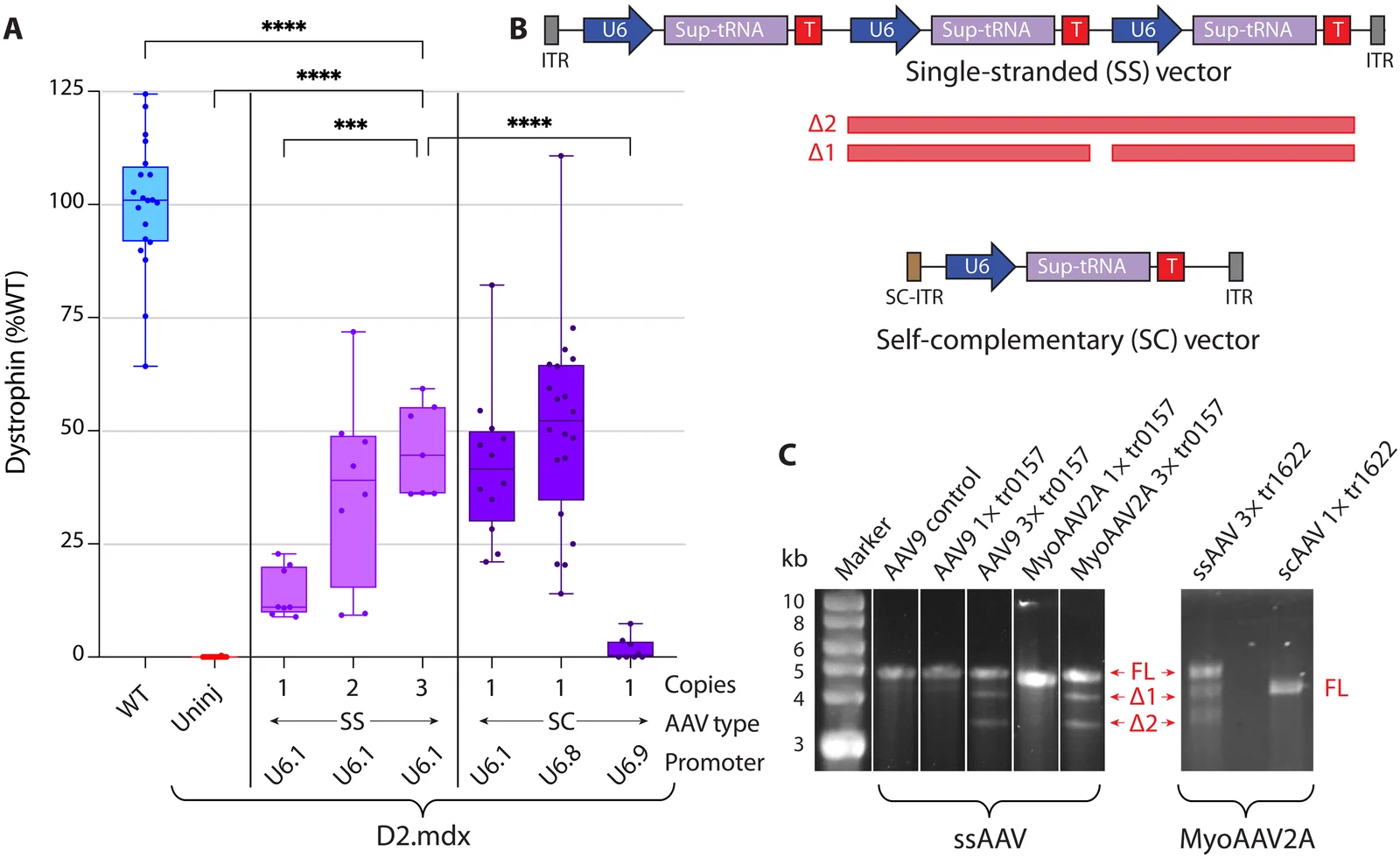
Optimization of sup-tRNA AAV vectors. (**A**) Quantification of dystrophin expression in TA muscles following IM injection of 1 × 10^11^ vg per muscle of ssAAV and scAAV vectors expressing sup-tRNA tr1622 under the control of the indicated promoters. Full-length dystrophin was measured by capillary Western blot 6 weeks after treatment. Plots show dystrophin levels in individual animals normalized to wild-type average. Box plots with individual data points showing middle line as the median; upper and lower quartiles as box limits; whiskers go down to the smallest value and up to the largest. Statistical analysis was performed using one-way ANOVA for the benchmark ssAAV 3-copy U6.1 vector versus all sup-tRNA treatment groups. ***P < 0.0005 and ****P < 0.00005. (**B**) Schematic diagram of sup-tRNA AAV expression cassettes used and AAV genome regions deleted following one- and two-copy truncation events (Δ1 and Δ2, respectively). (**C**) AAV vector genome integrity as determined by alkaline gel electrophoresis. AAV vectors (AAV9 and MyoAAV2A as indicated) contained the indicated sup-tRNAs at the indicated copy number. Sup-tRNAs were paired with upstream U6 promoters. 1×, single copy of the sup-tRNA; 3×, three copies of the sup-tRNA; control, AAV vector without sup-tRNA; FL, full-length AAV vector.

As an additional strategy to increase sup-tRNA expression and enable lower AAV doses, we tested alternatives to the U6 promoter. The most widely used U6 RNA Pol III promoter—and the one used in our prior AAV vector designs—is the proximal promoter of the human U6-1 small nuclear RNA-encoding gene (U6-1). The human genome contains at least nine full-length U6 loci [U6-1 through U6-9 (*32*)]. To our knowledge, none except U6-1 have been characterized for their ability to enhance transcription or functional activity of a sup-tRNA expressed from an AAV vector. We therefore compared otherwise identical scAAV vectors that express sup-tRNA tr1622 under control of either the standard U6-1 promoter or the U6-8 or U6-9 promoters. The U6-8 vector resulted in greater rescue of full-length dystrophin than U6-1, although it did not reach statistical significance (Fig. 3C; 51.1 ± 22.3% versus 42.4 ± 16.5% of wild type). In contrast, the use of the U6-9 promoter substantially reduced readthrough efficiency.

To determine whether the above optimization strategies—when combined with our most efficacious engineered sup-tRNAs—were sufficient to achieve in vivo efficacy at doses of 1 × 10^14^ vg/kg or less, we used MyoAAV2A to deliver scAAV vectors expressing tr1622 and tr1626 under control of the U6-8 promoter (AAV-tr1622 and AAV-tr1626). AAVs were delivered by IV administration into 7-week-old D2.mdx mice at escalating doses of 1 or 3 × 10^13^ or 1 × 10^14^ vg/kg. Note that the highest dose in this experiment corresponds to the lowest systemic dose tested for AAV-tr0904. As with tr0904, both tr1622 and tr1626 were well tolerated with mice showing normal weight gain and no mortality (fig. S25). One factor that can limit therapeutic efficacy of sup-tRNAs—and virtually all other gene therapies—at low doses is diminishing biodistribution, which is an intrinsic property of the AAV capsid. In the case of cell-autonomous proteins such as dystrophin, AAV genomes must be present in targeted tissues at a level of approximately one genome per cell or more. As transduction drops below this critical threshold, an increasingly large percentage of cells will not receive even a single copy of the transgene and consequently fail to show rescue of disease pathology. We therefore assessed biodistribution of MyoAAV2A-delivered vector genomes at 12 weeks postinjection in skeletal muscles of D2.mdx mice. As expected, biodistribution—expressed as vector genomes per diploid genome (vg/dg)—was dose dependent and linear in all tissues examined and did not appear to be influenced by the sup-tRNA payload. In both quadriceps and gastrocnemius muscles, only doses of 1 × 10^14^ vg/kg and above resulted in tissue tropism that exceeded a threshold of 1 vg/dg (Fig. 4, A and B). Biodistribution in these tissues at 3 × 10^13^ vg/kg (the mid-dose used for AAV-tr1622 and AAV-tr1626) was only 0.6 ± 0.2 vg/dg in quadriceps and 0.2 ± 0.1 vg/dg in gastrocnemius.

**Fig. 4.**
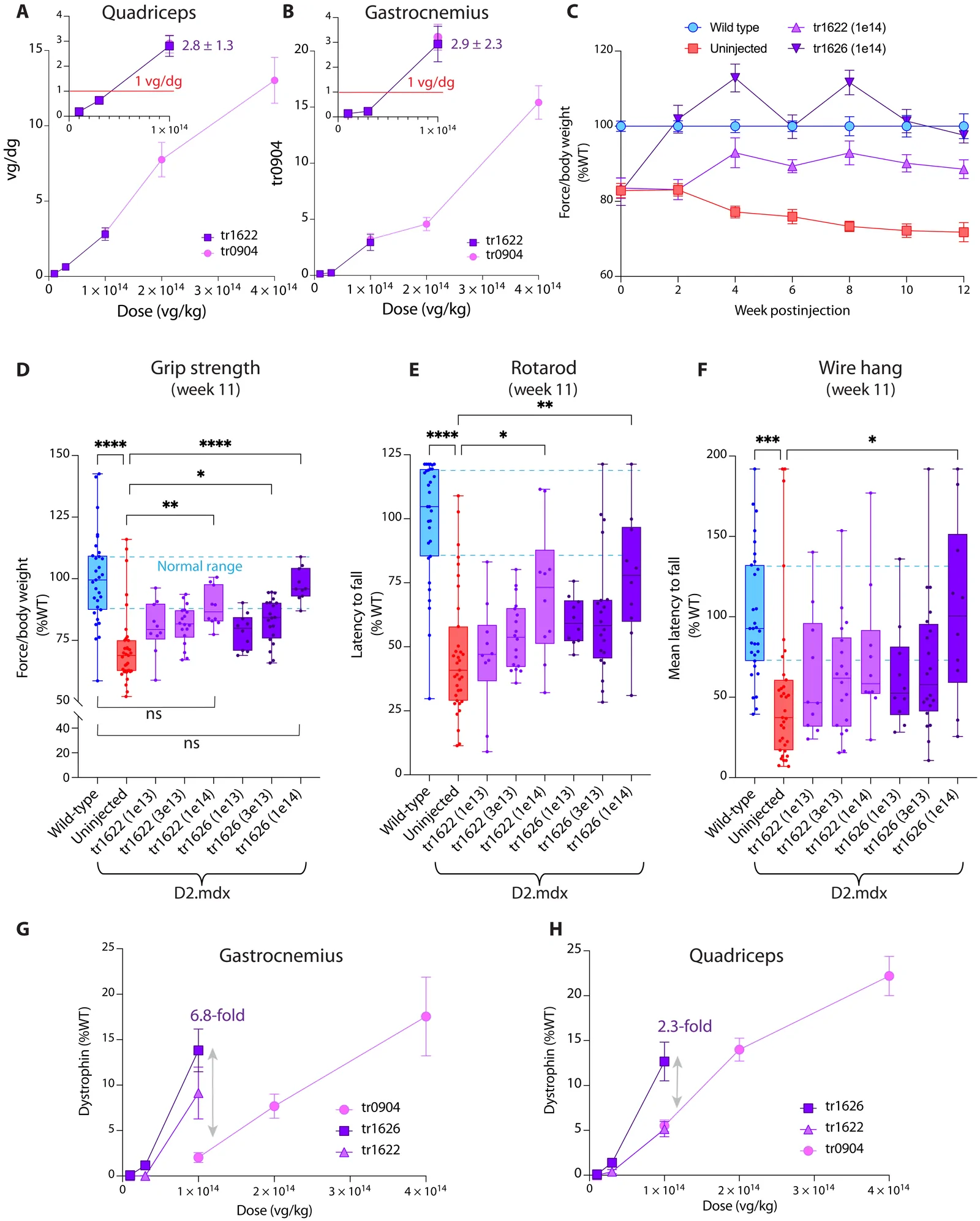
Functional rescue of muscle deficits at low AAV dose ranges. (**A**) Transduction of quadriceps and (**B**) gastrocnemius muscle following systemic AAV delivery at doses ranging from 3 × 10^13^ to 4 × 10^14^ vg/kg. Insets show expanded view of doses at 1 × 10^14^ and below. (**C**) Plots showing mean and SEM for grip strength over time at low in D2.mdx mice treated with AAV-tr1622 and AAV-tr1626 (1 × 10^14^ vg/kg). Mice were injected IV at 7 weeks of age. Grip strength assay was carried out at the time of injection and every 2 weeks thereafter. Plots show grip strength in individual animals normalized to wild-type average for each week tested. Mice were tested for grip strength (**D**), average latency to fall from a rotating Rotarod (**E**), and average latency to fall in a wire hand assay (**F**) at 11 weeks posttreatment. Quantification of dystrophin expression in gastrocnemius (**G**) and quadriceps muscles (**H**) following IV injection of AAV-tr0904, AAV-1622, and AAV-1626 at the indicated doses. Full-length dystrophin was measured by capillary Western blot. Dystrophin levels in individual animals were normalized to wild-type average from the same age. Plots show mean and SEM for each dose.

Muscle strength was assessed using a grip strength assay before the day of injection (week 0) and every 2 weeks thereafter. As expected, grip strength in untreated D2.mdx mice was significantly reduced relative to wild-type controls at all time points and continued to decline throughout the course of the study (Fig. 4C). Treatment at 1 × 10^14^ vg/kg led to significant and sustained dose-dependent improvement beginning at 4 weeks postinjection for trAAV-1622 and as early as 2 weeks postinjection for AAV-tr1626. Rather than merely preventing further functional decline, treatment with engineered sup-tRNAs was restorative, with dosed mice showing recovery in muscle strength deficits already present at the time of dosing. At 12 weeks posttreatment—the planned end of the study—grip strength was statistically indistinguishable from wild-type controls at 1 × 10^14^ vg/kg for both tr1622 and tr1626 (Fig. 4D). Despite low biodistribution at sub-10^14^ vg/kg doses, mice treated with AAV-tr1626 also showed significant improvement in grip strength at 3 × 10^13^ vg/kg. Both of the engineered sup-tRNAs also resulted in significant improvement in Rotarod performance at 1 × 10^14^ vg/kg (Fig. 4E). In addition, AAV-tr1626–treated mice showed a significant improvement in mean latency to fall in a wire hang assay at 1 × 10^14^ vg/kg (Fig. 4F).

AAV-tr1622 and AAV-tr1626 both resulted in dose-dependent rescue of full-length dystrophin protein in muscle tissues (Fig. 4, G and H, and fig. S26). Consistent with functional outcomes, dystrophin levels were significantly higher at 1 × 10^14^ vg/kg than either of the two lower doses tested. This was most likely a consequence of MyoAAV2A transduction dropping below the critical threshold (1 vg/dg) at lower doses, limiting dystrophin rescue to a subset of muscle fibers. AAV-tr01626 in particular was significantly more efficient at restoring full-length dystrophin in skeletal muscle than AAV-tr0904 when compared at the common dose of 1 × 10^14^ vg/kg (2.3-fold better in quadriceps; nearly 7-fold better in gastrocnemius; Fig. 4, G and H). This increase in efficiency came despite tr1626 being delivered using a scAAV vector that contained only a single copy of the sup-tRNA rather than three tandem copies for tr0904. These results show that full functional recovery of grip strength was achieved when dystrophin levels were around 10 to 15% of wild-type in skeletal muscle. This amount of restoration is equal to—or slightly better than—recently reported data from DMD mice treated with the exon 44 skipping antibody–oligonucleotide conjugate AOC 1044 (*33*). Full rescue of grip strength was achieved at very low AAV biodistribution in skeletal muscles (2 to 3 vg/dg). This transduction profile is considerably lower than biodistribution reported in patients treated with microdystrophin gene therapies that use myotropic AAVs: RGX-202 (5.4 to 9.8 vg/dg; REGENEXBIO Inc.; https://regenxbio.gcs-web.com/news-releases/news-release-details/regenxbio-initiates-pivotal-phase-affinity-duchenner-trial-rgx) and SGT-003 (7.6 to 28.6 vg/dg; Solid Biosciences; https://globenewswire.com/news-release/2025/02/18/3027621/0/en/Solid-Biosciences-Reports-Positive-Initial-Clinical-Data-from-Next-Generation-Duchenne-Gene-Therapy-Candidate-SGT-003.html).

## DISCUSSION

This study establishes sup-tRNAs as a therapeutic approach for DMD caused by nonsense mutations, addressing a critical unmet medical need where no current therapy restores full-length dystrophin. Through a combination of in vitro and in vivo screening, we identified highly active, engineered sup-tRNAs—containing sequence changes both within and outside of the anticodon—that significantly outperformed parental sup-tRNAs—which differ from endogenous tRNAs only at the anticodon. Additional work is required to determine why specific changes outside of the anticodon improve sup-tRNA efficiency. To successfully suppress a nonsense mutation, a sup-tRNA must undergo the same tightly regulated posttranscriptional processes that affect endogenous tRNAs. These include 5′ and 3′ end processing, a wide variety of posttranscriptional enzymatic modifications at key positions, folding into a stereotypical three-dimensional L-shaped structure, aminoacylation to form aminoacyl-tRNA, and assembly of a ternary complex for delivery to the ribosomal A site (*34*, *35*). Sequence changes to the body of the sup-tRNA have the potential to modulate any of these processes and affect fundamental properties such as half-life, charging, and tRNA-protein and tRNA-ribosome interactions.

When delivered systemically via a muscle-targeting AAV vector, engineered sup-tRNAs targeting UAA nonsense mutations achieved robust rescue of full-length dystrophin leading to functional recovery in the stringent D2.mdx model within 12 weeks. Comprehensive proteomic analysis revealed that sup-tRNA treatment produced extensive normalization of disease-associated molecular signatures, reversing pathological changes in key dystrophic pathways including the dystrophin-glycoprotein complex, calcium handling machinery, cytoskeletal networks, and tissue repair mechanisms. Off-target analysis demonstrated that sup-tRNAs are capable of high selectivity, with no appreciable increase in the background level of native stop codon readthrough at therapeutically effective doses, indicating that cellular quality control mechanisms successfully discriminate between pathological and physiological termination events. Formal immunogenicity studies evaluating anti-AAV and anti-transgene immune responses are planned as part of the IND-enabling program to further characterize the safety profile of this therapeutic approach.

A key advantage of sup-tRNAs over existing microdystrophin therapies is their potential to restore full-length dystrophin in the ∼15% of patients with DMD and nonsense mutations. UAA stop codons account for ∼27% of nonsense mutations in patients based on a global database of 7149 DMD mutations (*36*). The therapeutic advantage of full-length dystrophin over microdystrophins can perhaps best be illustrated by the relatively modest levels of dystrophin that are restored by Food and Drug Administration–approved exon-skipping therapies such as eteplirsen (EXONDYS 51; Sarepta) and golodirsen (VYONDYS 53; Sarepta). Although exon skipping therapeutics do not restore full-length dystrophin, they produce a nearly full-length protein that is presumably much closer to native dystrophin in terms of functionality, protein-protein interactions, and biomechanical properties than microdystrophins. Functional benefits have been documented in patients with DMD treated with eteplirsen (*37*) and golodirsen (*38*) despite the fact that these therapeutics only result in average dystrophin protein levels of 0.44 to 0.93% and 1.02 ± 1.03% of normal, respectively (www.sareptadmd.com/exondys51/ and www.sareptadmd.com/vyondys53/). AOC 1044 (Del-zota), a second-generation exon skipper that combines a monoclonal antibody against the transferrin receptor and a phosphorodiamidate morpholino oligomer targeting exon 44 for enhanced muscle targeting, restores dystrophin to 6 to 11% of normal in DMD mice (*33*). Our data show that systemically delivered engineered sup-tRNAs substantially outperform eteplirsen and golodirsen—and match or exceed Del-zota—in all muscles examined when delivered systemically at 1 × 10^14^ vg/kg in mice (Fig. 4F). Our approach also differs fundamentally from small-molecule readthrough agents such as ataluren (Translarna), which showed controversial efficacy in clinical trials and whose mechanism of action has been questioned. Unlike ataluren, which relies on promiscuous ribosomal misreading with uncertain amino acid incorporation (*39*), tRNAs provide a direct, programmable mechanism for decoding specific premature stop codons while inserting the intended amino acid.

The implications of sup-tRNA therapeutics extend far beyond DMD, as nonsense mutations represent the molecular basis for a substantial fraction of all human genetic diseases (*6*). Unlike gene-specific approaches that address individual proteins, sup-tRNAs target a fundamental mutation class, offering a unified therapeutic platform for diverse genetic conditions including other neuromuscular disorders, cardiomyopathies, and inherited diseases across multiple organ systems. The demonstrated safety profile and lack of interference with normal cellular processes position this approach as particularly attractive for rare diseases where traditional drug development is challenging and potentially cost prohibitive. Furthermore, the dose-dependent efficacy observed here suggests that therapeutic windows can be optimized to maximize therapeutic benefit while minimizing any potential off-target effects. This demonstrates the potential to further enhance sup-tRNA potency to achieve even higher levels of protein rescue and/or therapeutic efficacy at lower AAV doses.

The fundamental advantage of sup-tRNAs lies in their ability to restore full-length, functional proteins, a transformative capability that distinguishes them from current strategies that produce only truncated or modified dystrophin proteins (*5*). This complete restoration promises more durable and comprehensive therapeutic rescue for patients. Beyond DMD, the broad applicability of sup-tRNAs across the spectrum of genetic diseases caused by nonsense mutations represents a true paradigm shift toward precision mutation-class therapies. With more than 1800 genetic diseases linked to nonsense mutations affecting millions of patients worldwide, sup-tRNAs offer unprecedented potential to transform care for vast populations now facing untreatable genetic disorders. Our ongoing work is extending these engineering approaches to sup-tRNAs targeting UAG and UGA stop codons, which, together with UAA stop codons, account for all nonsense mutations and would expand the addressable DMD patient population.

## MATERIALS AND METHODS

### AAV production

All AAV sup-tRNA expression cassettes were generated by PackGene (Guangzhou, China). MyoAAV2A virus was produced by PackGene via triple transfection of adherent HEK293T cells. Titer of purified virus was determined using droplet digital polymerase chain reaction (ddPCR). AAV virus stocks were provided by the manufacturer in phosphate-buffered saline (PBS) containing 0.001% pluronic F-68 and stored at −70°C.

### Animals

Hemizygous male D2.B10 (DBA/2-congenic) *Dmd^mdx^* mice (also referred to as D2.mdx mice; the Jackson Laboratory stock no. 013141) were used for all experiments. This mouse model of DMD contains an inactivating nonsense mutation in the dystrophin gene. A C-to-T substitution in a CAA codon in exon 23 (ENSMUST00000114000 chrX:g.83803333C>T; c.2983C>T; p.Q995*) results in a termination codon (TAA) in place of a glutamine (Q) codon. The animal work in this study was approved by the Mispro Biotech Services Institutional Animal Care and Use Committee (protocol #2025-TVB-04), and studies were conducted in adherence to Good Laboratory Practice regulations (21 CFR Part 58).

### IM injection of AAVs

Twelve-week-old D2.mdx mice were dosed via IM administration (TA) at 1 × 10^11^ vg per muscle. Injections were performed on both TA muscles of mice under isoflurane anesthesia. Five animals were dosed per experimental condition. Untreated wild-type and D2.mdx hemizygous male mice were used as control groups. Animals were euthanized through CO_2_ exposure at 18 weeks of age (6 weeks posttreatment). Both right TA muscles were cut into small pieces and flash frozen in liquid nitrogen, stored at −70°C, and used for protein, DNA, and RNA analysis.

### IV injection of AAVs

D2.mdx mice were dosed via IV administration into the tail vein at the indicated ages. Untreated wild-type (“D2-WT”) and D2.mdx hemizygous male mice (“D2.mdx”) were used as control groups. Animals were euthanized through CO_2_ exposure at 12 weeks posttreatment. Heart muscle and skeletal muscles (gastrocnemius, quadriceps, diaphragm, TA, and triceps) were collected. Tissues were stored in OCT for immunohistochemistry or sectioned into smaller pieces and flash frozen in liquid nitrogen for protein, DNA, and RNA analysis.

### Grip strength

All-limb grip strength was measured using a grip strength apparatus (Bioseb). Mice were allowed to grip the grid with both forelimbs and hindlimbs. The mouse was pulled back steadily until the mouse lost grip on the grid. The grip strength meter digitally displayed the maximum force applied as the grasp was released. Six grip strength measurements were taken per mouse. Mice were given an interval of 1 min between trials. Average grip strength for each mouse was calculated after excluding the lowest and highest values and normalized to body weight.

### Rotarod

Motor function of mice was assessed on a Rotarod (AccuRotor four-channel, Omnitech Electronics). Mice were placed on the Rotarod (3.0-cm rotating cylinder) rotating at 4 rpm, and the speed of rotation was increased linearly to 40 rpm over the course of 5 min. The time to fall from the rod was measured. Each mouse was subjected to five trials, and the average latency to fall was recorded. Mice were given an interval of 15 min between trials.

### Wire hang

The test was performed using a horizontal taut wire (diameter, 2 to 3 mm; length, 40 to 60 cm) suspended ∼40 to 50 cm above a soft padded surface to prevent injury upon falling. Mice were gently grasped by the tail and lowered onto the wire so that only the forepaws initially grasped the wire (hindlimbs were lowered if necessary to prevent contact). The animal was released once a secure grip was established, and the latency to fall was recorded using a stopwatch. If the animal reached an end of the wire, it was repositioned at the center and timing continued. A maximum cutoff of 120 s was applied per trial; if the animal remained hanging at 120 s, then the trial was terminated and scored as 120 s. Three consecutive trials were performed per animal, with 5- to 10-min rest intervals between trials to minimize fatigue. For each mouse, the mean latency to fall across the three trials was calculated.

### Protein extraction

Heart and skeletal muscles were homogenized in a 10% SDS lysis buffer. Total protein lysates were quantified using a BCA protein assay (Thermo Fisher Scientific, 23225) and normalized to 5 μg/μl for downstream analysis.

### Dystrophin quantification by capillary Western blot

Three micrograms of total protein was used to detect full-length dystrophin by capillary Jess Automated Western blot (ProteinSimple, 004650) based on the manufacturer’s instructions using an anti-dystrophin antibody (1:250 dilution; Abcam, ab154168). Dystrophin signal intensity was normalized to total protein signal intensity. For a precise measure of dystrophin protein expression levels, a standard curve was established using pooled untreated wild-type (D2-WT) and pooled untreated mutant (D2.mdx) lysates at increasing concentrations (1, 5, 10, 25, 50, 75, and 100% wild-type levels, *R*^2^ > 0.98). Resulting dystrophin signals from samples were then quantified to the standard curve.

### Dystrophin quantification by immunohistochemistry

Heart and skeletal muscles were transversally bisected and embedded in OCT compound. Cryo-embedded tissues were sectioned into ∼7- to 10-μm-thick cross sections using a microtome cryostat. Cross sections were permeabilized using 1% Triton X-100, blocked in PBS with 3% bovine serum albumin, and 2% normal goat serum, and stained for dystrophin (Abcam, MA5-32565), laminin-2 (Abcam, L0663), and 4′,6-diamidino-2-phenylindole to determine the number of muscle fibers positive for dystrophin expression. Cross sections were mounted for visualization, and pictures were acquired using a FV3000 Olympus confocal microscope (20× objective). Analysis was done using QuPAth software with a custom analysis pipeline.

### Liquid chromatography–tandem mass spectrometry

Proteomic profiling was performed by Allumiqs (Sherbrooke, QC, Canada). Total protein concentration was confirmed by a microBCA assay. Fifty-five micrograms of protein per sample was used. All samples were aliquoted into a plate and randomized. The aliquoted volumes were brought up to 42 μl in PAC lysis buffer [1% SDS and 50 mM tris (pH 8)] and subject to a Protein Aggregation Capture (PAC) workflow using hydroxyl coated beads. Samples were reduced with 10 mM dithiothreitol for 15 min at 65°C, equilibrated to room temperature for 5 min, and alkylated with 15 mM iodoacetamide for 30 min at room temperature in the dark. Beads were conditioned by combining 45-μl aliquots of slurry with 900 μl of 70% acetonitrile (ACN), mixed for 30 s, and isolated on a magnetic rack; the supernatant was discarded; and a second wash cycle was performed. The conditioned beads were suspended in 450 μl of 70% ACN. Fifty microliters of beads was combined with 50 μl of each alkylated sample. Protein aggregation was induced by addition of 211 μl of neat ACN, mixed on a thermoshaker (700 rpm) for 1 min followed by 15 min without shaking. The bead-bound fraction was isolated on a magnetic rack, and the supernatant was discarded. The bead-bound samples were washed with 900 μl of neat ACN, isolated on the magnetic rack, washed with 900 μl of 70% ethanol, isolated on the magnetic rack, and lastly washed with 900 μl of 70% methanol. The washed bead-bound samples were suspended in 200 μl of PAC digestion buffer [25 mM ammonium bicarbonate (pH 8) and 1:40 trypsin/Lys-C] and digested overnight at 37°C with gentle mixing (900 rpm). The following day, samples were spiked with 20 μl of a master mix of heavy peptides (1 μg/ml). Samples were acidified with 2% formic acid, and the peptides were purified by reversed phase SPE. Peptides were resuspended in 60 μl of H_2_O and 0.1% formic acid. Once resuspended, 4 μl of each sample was pooled to be injected as GPF samples to be used for spectral library generation. Samples and pools were transferred to HPLC vials with insert and injected on a 60-min LC-MS/MS gradient.

Acquisition was performed with an Orbitrap Exploris 480 (Thermo Fisher Scientific) equipped with a FAIMS Pro Duo interface and coupled to a Neo Vanquish (Thermo Fisher scientific) via an EASY-Spray source. Thermo Fisher Scientific Xcalibur 4.6 software was used to control the instrument and acquisitions. The pool for the ion library was acquired in DIA acquisition mode using gas phase fractionation (GPF). The samples were analyzed in DIA acquisition mode. The spray voltage was set to 1.8 kV and heated capillary temperature at 300°C. The FAIMS Pro duo interface gas flow was set at 3.5 liters/min and CV = −45 V at standard resolution. Separation was performed on a reversed phase Aurora Elite 15 cm–by–75 m column (IonOpticks) which was maintained at 40°C. Five microliters of samples was injected into a 25 μl by a 25-μl split-loop design. For the 32-min LC gradient, the mobile phase consisted of the following solvent A [0.1% (v/v) formic acid in water] and solvent B [0.1% (v/v) formic acid in ACN] at a flow rate of 400 nl/min.

GPF acquisitions of pooled library-building samples and study samples were processed with DIA-NN (v. 1.8.1) against a customized proteomic database that included sequences resulting from UAA-to-glutamine substitutions at native stop codons. FDR was set to 0.01; maximum missed cleavages: 1; fragment mass/charge ratio (*m/z*): 100 to 1800; precursor *m/z*, 300 to 1250; mass accuracy: 20 parts per million; MBR; quant strategy: high accuracy; RT-dependent cross-run normalization. After quality validation of the run using the generated DIA-NN stats report (MS1 signal, MS2 signal, and MS1/MS2 signal ratios and number of precursor and identified proteins were checked), the report was processed in Python using an in-house pipeline to explore the protein quantification data. A protein centric approach was used to analyze and filter the raw DIANN output (maximum *q* value for precursors and proteins: 0.01, sum top five precursors, based on median intensity, to generate protein data). Missing values were filtered at a cutoff maximum of 25% and imputed using a K-nearest neighbor approach. Inter–LC-MS run variation was normalized (scaled) using RT-LOESS on log_2_ data before summing to protein level data. Following verification of homoscedasticity and normal distribution within the data, protein quantitation was analyzed using a one-way ANOVA comparison between uninjected D2.mdx and all injected D2.mdx groups with the Benjamini-Hochberg procedure for controlling FDR.

#### Customized databases for identification of stop codon readthrough events

LC-MS data were analyzed using a customized database. The mouse proteome (UPU000000589) was downloaded as a snapshot from UniProt as of 3 October 2022 and supplemented with a list of reference contaminants. It was then extended to cover (i) dystrophin truncation resulting from the D2.mdx nonsense mutation, (ii) dystrophin missense incorporations resulting from readthrough events of the D2.mdx nonsense mutation by potentially mischarged sup-tRNA, and (iii) readthrough events at native UAA stop codons across the proteome. For (i), an additional entry was added to the reference modifying NP_031894.1 to end at position 994, reflecting the Q995X nonsense mutation. For (ii), 19 alternate versions of NP_031894.1 were added to the reference with a Q995N substitution, where N can be any of the other 19 non-Gln amino acids. Last, for (iii), 5356 alternate versions of proteins with native UAA stop codons were generated by substituting a Q at the native stop position and extending the peptide sequence into the 3′UTR until the next in-frame stop codon. These database customizations were executed in a Jupyter Notebook using a Python 3 (https://www.python.org) kernel and pandas [The pandas development team (2024). pandas-dev/pandas: Pandas (v2.2.3). Zenodo. https://doi.org/10.5281/zenodo.13819579]. Additional sequence information relevant to the work was drawn from a combination of the UniProt (https://www.uniprot.org/), Entrez Protein (https://ncbi.nlm.nih.gov/protein), and Entrez Nucleotide (https://ncbi.nlm.nih.gov/nucleotide) databases.

#### Suppressor tRNA quantification by RT-ddPCR

tRNA cDNAs were synthesized using a modified protocol for Quant-M tRNA-Seq (*40*). Briefly, extracted RNAs were deacylated and ligated with adaptors (oT_124 to oT_127 and oT_130) that bind to the 5′ and 3′-CCA overhangs of mature tRNAs. Ligated tRNAs were reverse transcribed into cDNA using RT primer oT_123 and SuperScript IV Reverse Transcriptase (Thermo Fisher Scientific, catalog no. 18091050). RNA was then degraded by NaOH, and the cDNAs were stored at −20°C until further use.

tRNA levels were quantified by using the Nio + System from Stilla Technologies. Forward primers oT_1973 and oT_828 were designed for the sup-tRNA tr0904 and Gly-TCC-2 tRNA, respectively. The reverse primer oT_93 binds to a common sequence of all cDNAs. For each sample, the sup-tRNA levels were normalized to endogenous tRNAs.

#### Viral genome copy analysis

Primers oT_490 and oT_1583 were used to measure viral genome, and primers oT_762 and oT_763 were used to quantify ribonuclease P protein subunit p30 as diploid genome. Five nanograms of DNA was added in each respective ddPCR mix. The Nio + System from Stilla Technologies was used for the ddPCR reaction and quantification. Viral genome copies were normalized to diploid genome.

#### Suppressor tRNA synthesis for dual luciferase screening

Double-stranded DNA fragments encoding a suppressor tRNA with an upstream U6 promoter and a downstream RNA Pol III termination signal were synthesized as gBlocks (Integrated DNA Technologies).

### HEK293 cell culture and transfection

A total of 2.5 × 10^4^ HEK293 cells (AAV-293 cells; Agilent, 240073) were plated per well in a 96-well plate with clear-bottom and opaque sides in cell culture medium containing 90% Dulbecco’s modified Eagle’s medium and 10% fetal bovine serum for 24 hours. For each transfection, one tube containing 0.3 μl of Lipofectamine 3000 (Thermo Fisher Scientific, L3000008) in 5 μl of Opti-MEM was mixed with a second tube containing 10 ng of a plasmid expressing the dual luciferase reporter, 10 ng of a gBlock expressing the suppressor tRNA, and 0.02 μl of P3000 in 5 μl of Opti-MEM. Combined transfection reagents were mixed gently, incubated for ∼10 min at room temperature, added to the cell culture medium, and incubated at 37°C for 24 hours.

### Dual luciferase assay

Dual luciferase assays were performed using the Nano-Glo Dual-Luciferase Reporter Assay System (Promega, N1610). Cell culture medium was removed at 24 hours posttransfection and replaced with 80 μl of fresh medium per well. Eighty microliters of ONE-Glo EX reagent was added per well and incubated for ∼10 min to allow for cell lysis to occur. Firefly luminescence was measured in a luminometer (Thermo Fisher Scientific, Varioskan LUX Multimode Microplate Reader). Eighty microliters of NanoDLR Stop and Glo Reagent was added to each well and incubated for 10 min. NanoLuc luminescence was measured in a luminometer. The ratio of NanoLuc luminescence to Firefly luciferase luminescence was then calculated for each well and normalized to the wild-type reporter.

## Supplementary Materials

### This PDF file includes:

Supplementary Text Figs. S1 to S26 Tables S1 and S2 References
