# Supplementary material for: Engineering suppressor tRNAs for effective treatment of Duchenne muscular dystrophy
Source: Sci Adv. 2026 Sep 9;12(37):eaeg3466. doi: 10.1126/sciadv.aeg3466 (PMC13557089; doi:10.1126/sciadv.aeg3466)
Supplement: Supplementary file 1 [file sciadv.aeg3466_sm.pdf]

Science Advances
AAAS

Supplementary Materials for

Engineering suppressor tRNAs for effective treatment of Duchenne muscular dystrophy

Julien Oury *et al.*

Corresponding author: Peter M. Eimon, peimon@tevard.com; Jeff Coller, jmcoller@jhmi.edu; Harvey Lodish, lodish@wi.mit.edu

Sci. Adv. **12**, eaeg3466 (2026)
DOI: 10.1126/sciadv.aeg3466

This PDF file includes:

Supplementary Text
Figs. S1 to S26
Tables S1 and S2
References

Supplementary Materials

Supplementary Materials and Methods

U6-1, U6-8, and U6-9 promoters

The following sequences were used for AAV vectors containing U6-1 (a.k.a. U6) and U6-8 RNA Pol III promoters:

U6-1

GAGGGCCTATTTCCCATGATTCCTTCATATTTGCATATACGATACAAGGCTGTTAGAGAGATAA
TTAGAATTAATTTGACTGTAAACACAAAGATATTAGTACAAAATACGTGACGTAGAAAGTAATA
ATTTCTTGGGTAGTTTGCAGTTTTAAAATTATGTTTTAAAATGGACTATCATATGCTTACCGTA
ACTTGAAAGTATTTCGATTTCTTGGCTTTATATATCTTGTGGAAAGGACGAAACACC

U6-8

GCTCCGCGGCACGAGAACTCAAAGCCCCGGGGCCTGGGTCCCACGCGGGGTCCCTTACCCAGGG
TGCCCCGGGCGCTCATTTGCATGTCCCACCCAACAGGTAAACCTGACAGGTCATCGCGGCCAGG
TACGACCTGGCGGTCAGAGCACCAAACATACGAGCCTTGTGATGAGTTCCGTTGCATGAAATTC
TCCCAAAGGCTCCAAGATGGACAGGAAAGGGCGCGGTTCGGTCACCGTAAGTAGAATAGGTGAA
AGACTCCCGTGCCTTATAAGGCCTGTGGGTGACTTCTTCTCAAC

U6-9

GCCTGAGGCGTGGGGCCGCCTCCCAAAGACTTCTGGGAGGGCGGTGCGGCTCAGGCTCTGCCCC
GCCTCCGGGGCTATTTGCATACGACCATTTCCAGTAATTCCCAGCAGCCACCGTAGCTATATTT
GGTAGAACAACGAGCACTTTCTCAACTCCAGTCAATAACTACGTTAGTTGCATTACACATTGGG
CTAATATAAATAGAGGTTAAATCTCTAGGTCATTTAAGAGAAGTCGGCCTATGTGTACAGACAT
TTGTTCCAGGGGCTTTAAATAGCTGGTGGTGGAACTCAATATTC

Dual luciferase reporters

Dual luciferase reporters consisted of an N-terminal firefly luciferase fused in-frame to a C-terminal NanoLuc luciferase. The two luciferase reporters were separated by a P2A self-cleaving peptide. Nonsense reporters introduced an inactivating glutamine-to-UAA, UAG, or UGA nonsense mutation at position 586 (Q586*). Nonsense mutation site is underlined in bold in the wild-type dual luciferase reference sequence below.

atggaagatgccaaaaacattaagaagggcccagcgccattctacccactcgaagacgggaccg
ccggcgagcagctgcacaaagccatgaagcgctacgccctggtgcccggcaccatcgcctttac
cgacgcacatatcgaggtggacattacctacgccgagtacttcgagatgagcgttcggctggca
gaagctatgaagcgctatgggctgaatacaaaccatcggatcgtggtgtgcagcgagaatagct
tgcagttcttcatgcccgtgttgggtgccctgttcatcggtgtggctgtggccccagctaacga

catctacaacgagcgcgagctgctgaacagcatgggcatcagccagcccaccgtcgtattcgtg
agcaagaaagggctgcaaaagatcctcaacgtgcaaaagaagctaccgatcatacaaaagatca
tcatcatggatagcaagaccgactaccagggcttccaaagcatgtacaccttcgtgacttccca
tttgccacccggcttcaacgagtacgacttcgtgcccgagagcttcgaccgggacaaaaccatc
gccctgatcatgaacagtagtggcagtaccggattgcccaagggcgtagccctaccgcaccgca
ccgcttgtgtccgattcagtcatgcccgcgaccccatcttcggcaaccagatcatccccgacac
cgctatcctcagcgtggtgccatttcaccacggcttcggcatgttcaccacgctgggctacttg
atctgcggctttcgggtcgtgctcatgtaccgcttcgaggaggagctattcttgcgcagcttgc
aagactataagattcaatctgccctgctggtgcccacactatttagcttcttcgctaagagcac
tctcatcgacaagtacgacctaagcaacttgcacgagatcgccagcggcggggcgccgctcagc
aaggaggtaggtgaggccgtggccaaacgcttccacctaccaggcatccgccagggctacggcc
tgacagaaacaaccagcgccattctgatcacccccgaaggggacgacaagcctggcgcagtagg
caaggtggtgcccttcttcgaggctaaggtggtggacttggacaccggtaagacactgggtgtg
aaccagcgcggcgagctgtgcgtccgtggccccatgatcatgagcggctacgttaacaaccccg
aggctacaaacgctctcatcgacaaggacggctggctgcacagcggcgacatcgcctactggga
cgaggacgagcacttcttcatcgtggaccggctgaagagcctgatcaaatacaagggctaccag
gtagccccagccgaactggagagcatcctgctgcaacaccccaacatcttcgacgccggggtcg
ccggcctgcccgacgacgatgccggcgagctgcccgccgcagtcgtcgtgctggaacacggtaa
aaccatgaccgagaaggagatcgtggactatgtggccagccaggttacaaccgccaagaagctg
cgcggtggtgttgtgttcgtggacgaggtgcctaaaggactgaccggcaagttggacgcccgca
agatccgcgagattctcattaaggccaagaagggcggcaagatcgccgtgggaagcggagctac
taacttcagcctgctgaagcaggctggagacgtggaggagaaccctggacctatggtcttcaca
ctcgaagatttcgttggggactggcga**cag**acagccggctacaacctggaccaagtccttgaac
agggaggtgtgtccagtttgtttcagaatctcggggtgtccgtaactccgatccaaaggattgt
cctgagcggtgaaaatgggctgaagatcgacatccatgtcatcatcccgtatgaaggtctgagc
ggcgaccaaatgggccagatcgaaaaaatttttaaggtggtgtaccctgtggatgatcatcact
ttaaggtgatcctgcactatggcacactggtaatcgacggggttacgccgaacatgatcgacta
tttcggacggccgtatgaaggcatcgccgtgttcgacggcaaaaagatcactgtaacagggacc
ctgtggaacggcaacaaaattatcgacgagcgcctgatcaaccccgacggctccctgctgttcc
gagtaaccatcaacggagtgaccggctggcggctgtgcgaacgcattctggcgaattctcacgg
ctttccgcctgaggttgaagagcaagccgccggtacattgcctatgtcctgcgcacaagaaagc
ggtatggaccggcacccagccgcttgtgcttcagctcgcatcaacgtctaa

Suppressor tRNA synthesis for dual luciferase screening

Double-stranded DNA (dsDNA) fragments encoding a suppressor tRNA with an upstream U6 promoter and a downstream RNA polymerase III (Pol III) termination signal were synthesized as gBlocks (Integrated DNA Technologies). Reference sequence below shows an exemplary dsDNA fragment with U6 promoter (underlined), tr0904 (bold), and PolII terminator (italic).

<u>GAGGGCCTATTTCCCATGATTCCTTCATATTTGCATATACGATACAAGGCTGTTAGAGAGATAA</u>
<u>TTAGAATTAATTTGACTGTAAACACAAAGATATTAGTACAAAATACGTGACGTAGAAAGTAATA</u>

ATTTCTTGGGTAGTTTGCAGTTTTAAAATTATGTTTTAAAATGGACTATCATATGCTTACCGTA ACTTGAAAGTATTTCGATTTCTTGGCTTTATATATCTTGTGGAAAGGACGAAACACC**GGCCCCA TGGTGTAATGGTTAGCACTCTGGTCTTTAAAACCAGCGATCCGAGTTCAAATCTCGGTGGGACC T**GTCC*TTTTTTT*CGTTCTGCGATCCGCTAGACGGCAATTTTTGGAATAAGGCTGTTCCCCGGC GATCCTAGAATAATTGTCGTACATCCGCCCAACCCGT

Supplementary Discussion

AAV-tr0904 results in normalization of proteins and pathways that are dysregulated in DMD patients and D2.mdx mice

Many differentially expressed proteins detected in our analysis are known to be dysregulated in DMD patients and the *mdx* mouse model. Utrophin (Utrn), an autosomal paralog of dystrophin, is upregulated DMD patients and undergoes re-distribution from the neuromuscular junction to the sarcolemma, where it is hypothesized to partially compensate for lack of dystrophin (*41*), (*42*). Utrn was found to be abnormally upregulated in untreated D2.mdx mice and exhibited a significant dose-dependent decrease in response to AAV-tr0904 treatment (fig. S19). Similarly, members of the annexin A protein family (Anxa1, Anxa2, and Anxa5), which are thought to participate in protection and repair of skeletal muscle sarcolemma, were pathologically elevated in dystrophic tissue and underwent a significant reversion to WT levels in suppressor-treated mice (fig. S18) (*43*). The extracellular matrix glycoprotein fibronectin (Fn1) is upregulated during tissue repair and fibrosis - hallmarks of DMD pathology - and has been proposed as a serum biomarker for disease progression in patients (*44*). AAV-tr0904 treatment was found to significantly reduce Fn1 elevation at all doses (fig. S19).

Proteome dysregulation impacting core proteins of the dystrophin-glycoprotein complex (DGC) and components of the wider dystrophin-associated network is well-documented in dystrophic muscle (*45*). AAV-tr0904-treated D2.mdx mice showed normalization of many differentially expressed proteins in this network including cytoskeletal proteins such at Synemin (Synm), Plectin (Plec), members of the tubulin superfamily (Tubb2a, Tubb5, Tubb6, and Tuba1c), and Vimentin (Vim) (fig. S19). Some, but not all, core members of the DGC also exhibited significantly reduced differential expression following sup-tRNA treatment (fig. S20). Sarcospan (Sspn), alpha-1-syntrophin (Snta1), and alpha-sarcoglycan (Sgca) all showed significant dose-dependent reversion toward WT levels. Other members of the DGC trended toward WT levels following treatment without reaching significance.

Growing evidence links abnormal elevation of cytosolic Ca^{2+} levels to muscle pathology and dysfunction in dystrophin-deficient muscle (*46*), (*47*). We detected a number of differentially expressed proteins associated with Ca^{2+} binding and handling in untreated D2.mdx mice that have previously been linked to DMD, including S100A1 proteins, calreticulin, and subunits of voltage-

gated, L-type Ca^{2+} channels such as Cacnb1. All underwent dose-dependent reversion toward WT levels in sup-tRNA-treated cohorts (fig. S21).

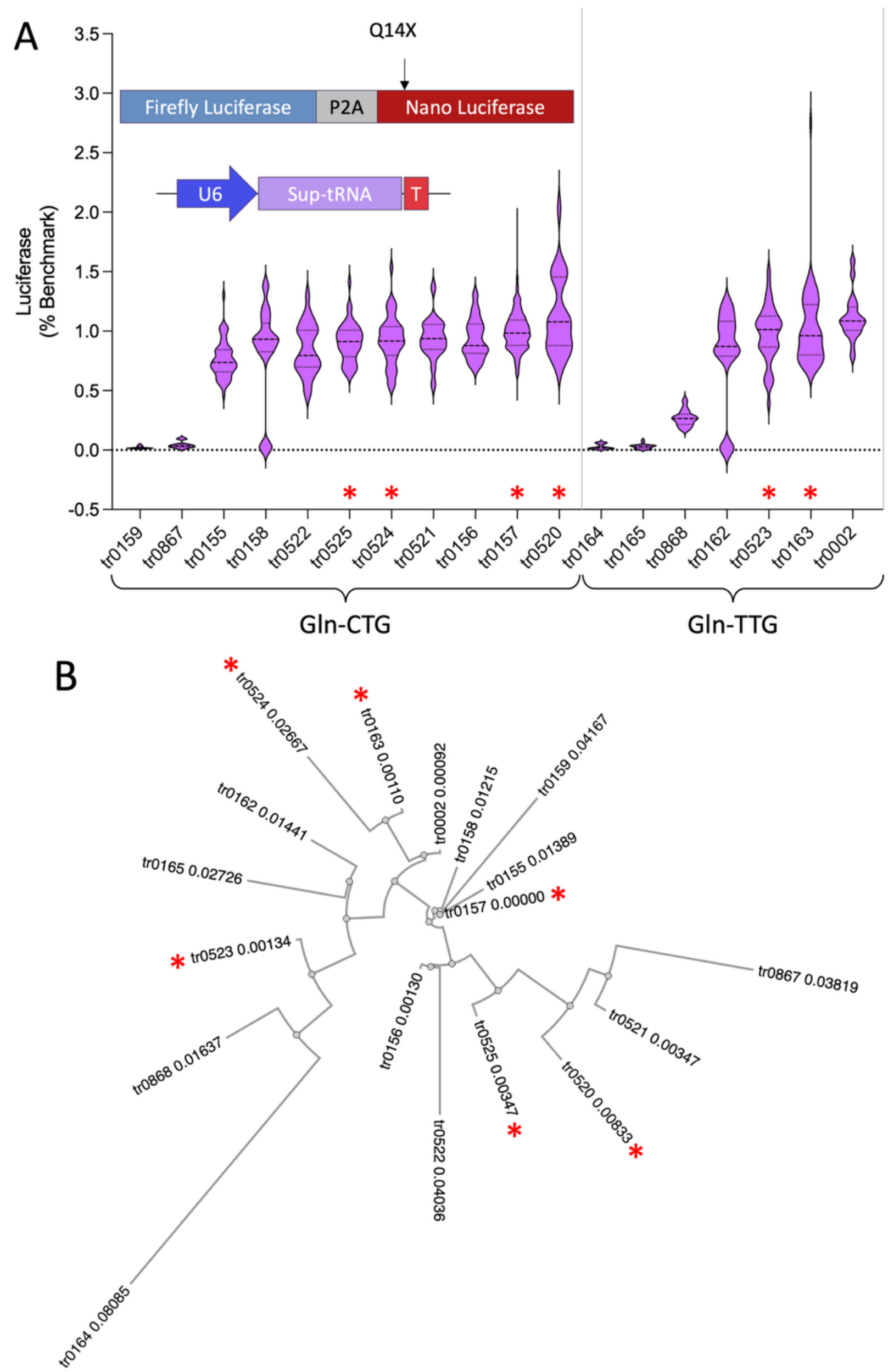

Fig. S1. *In vitro* screening of parental sup-tRNAs derived from endogenous Gln-tRNAs (**A**) Diagram of the dual Luciferase assay used to screen suppressor tRNAs and results from 18 $tRNA^{Gln}UUA$ candidates. Data are normalized to a benchmark $tRNA^{Gln}UUA$ parental sup-tRNA (tr0157) that was included as an internal control on all plates screened. n=6 or more technical replicates per group. U6, human U6 promoter; P2A, P2A self-cleaving peptide; T, RNA polymerase III termination signal. Red asterisks indicate sup-tRNAs selected for follow-on *in vivo* studies (**B**) Phylogenetic tree resulting from multiple sequence alignment via Clustal Omega of all $tRNA^{Gln}UUA$ parental sup-tRNAs tested. Red asterisks indicate sup-tRNAs selected for follow-on *in vivo* studies.

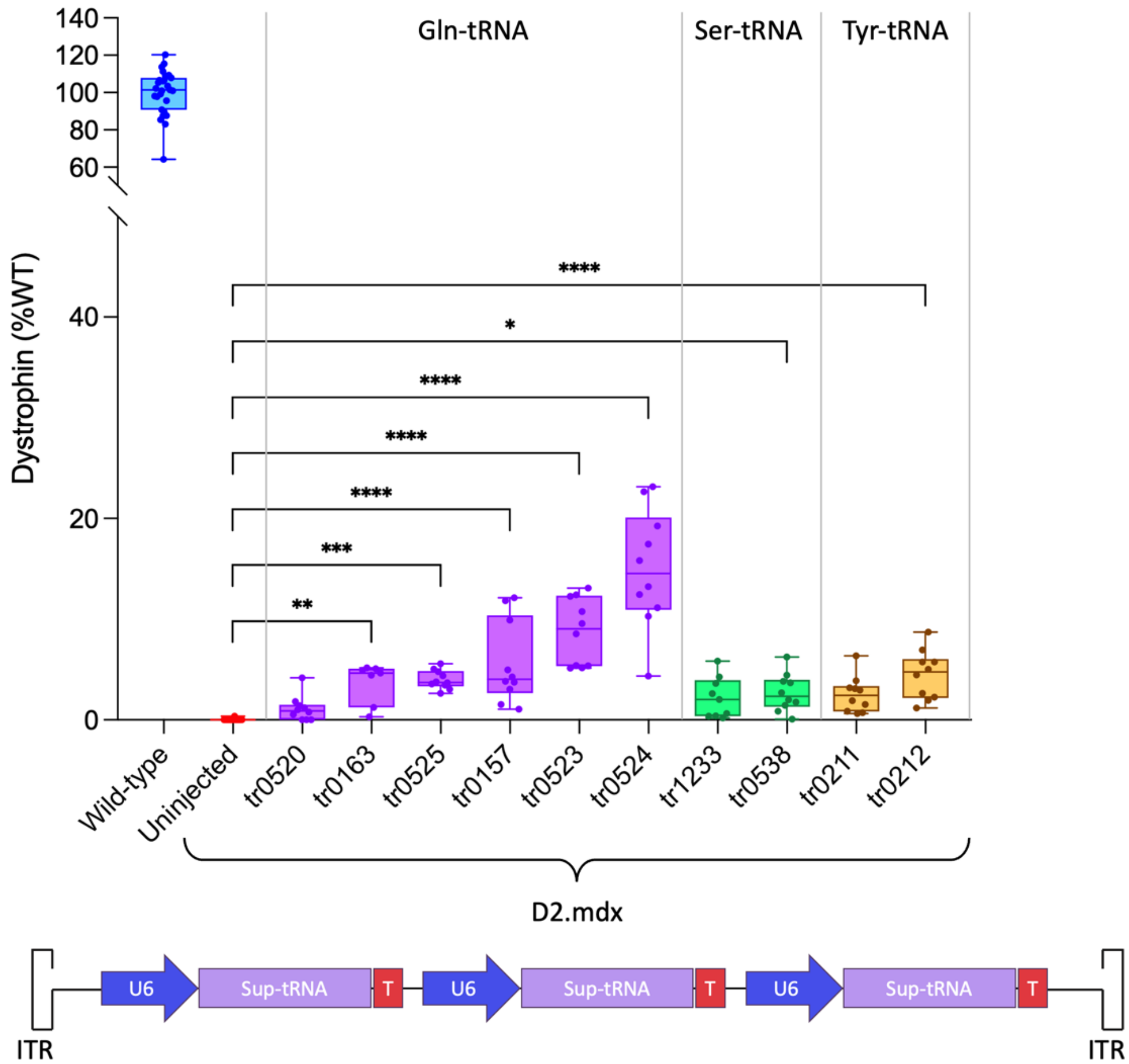

Fig. S2. *In vivo* screening of parental sup-tRNAs derived from endogenous Gln-tRNAs
Schematic diagram of the sup-tRNA AAV expression cassette and quantification of dystrophin expression in TA muscles of D2.mdx mice following intramuscular injection of 1×10^{11} vg per muscle of AAV expressing the indicated parental sup-tRNAs (n=7-10). Full-length dystrophin was measured by capillary western blot 6 weeks after treatment. Plots show dystrophin levels in individual animals normalized to wild-type average. U6, human U6-1 promoter; T, RNA polymerase III termination signal; ITR, inverted terminal repeats. Box plots with individual data points showing middle line as the median; upper and lower quartiles as box limits; whiskers go down to the smallest value and up to the largest. Statistical analysis was performed using one-way ANOVA for uninjected D2.mdx mice versus all sup-tRNA treatment groups. *<0.05, **<0.005, ***<0.0005, ****<0.00005.

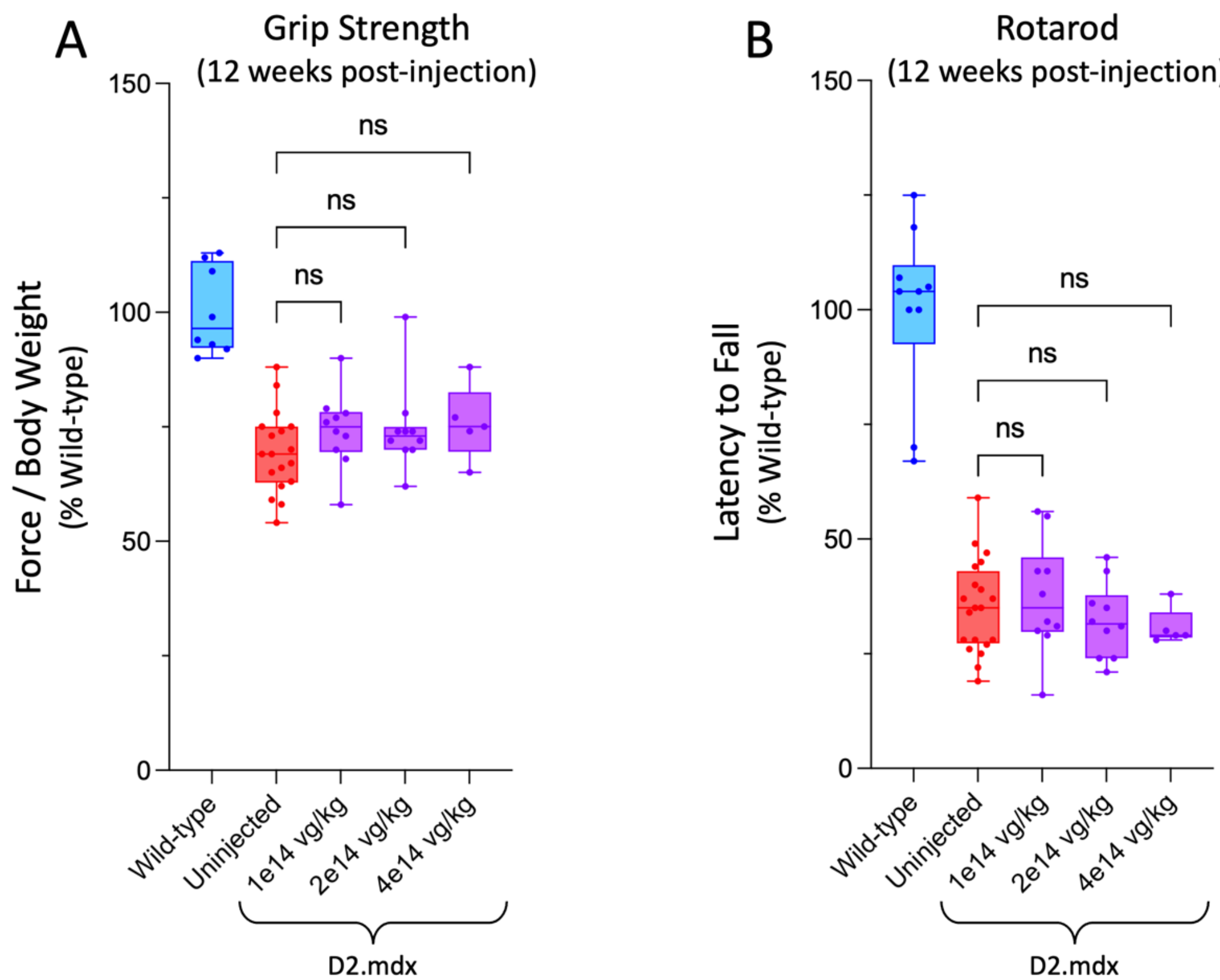

Fig. S3. Motor function is not improved in dystrophic mice following systemic delivery of parental sup-tRNAs. (**A**) Untreated wild-type, untreated D2.mdx, and AAV-tr0524-treated D2.mdx mice were tested for grip strength and (**B**) average latency to fall from a rotating Rotarod at 11 weeks post-treatment. D2.mdx mice were injected intravenously with AAV-tr0524 at 12 weeks of age at 1, 2, or $4×10^{14}$ vg/kg. Rotarod and grip strength assays were carried out at 11 weeks post-injection. Plots show individual data normalized to wild-type average. Box plots with individual data points showing middle line as the median; upper and lower quartiles as box limits; whiskers go down to the smallest value and up to the largest. Plots show individual data. Statistical analysis was performed using one-way ANOVA test for uninjected D2.mdx mice versus all sup-tRNA treatment groups.

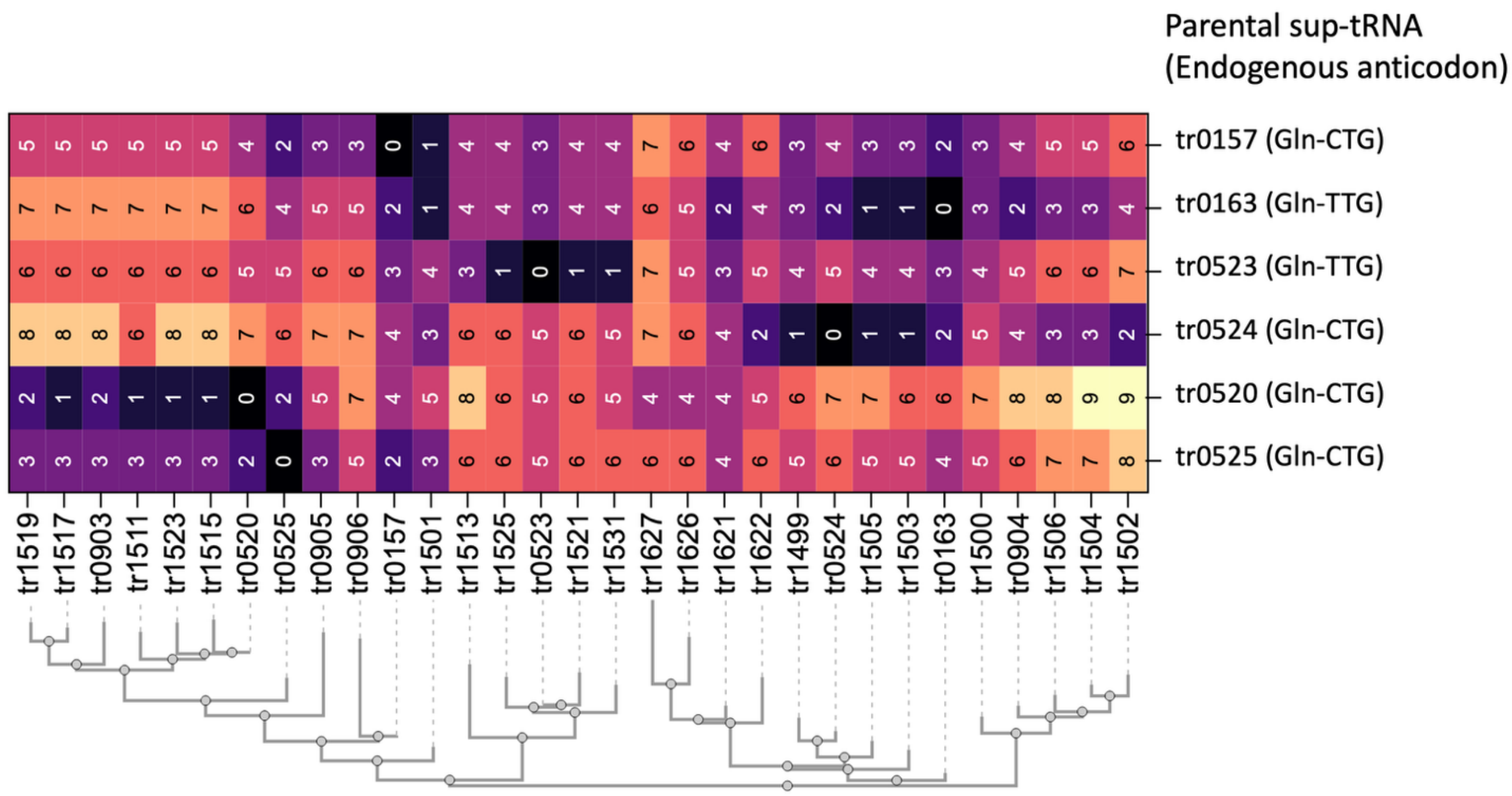

Fig. S4. Sequence similarity between parental and engineered sup-tRNAs. All tRNA^{Gln}UUA sup-tRNAs that were screened *in vivo* in D2.mdx mice are clustered by sequence similarity along the x-axis. The minimum number of nucleotide changes required to convert each sup-tRNA into the six parental sup-tRNAs is indicated in the heat map. The anticodon of the endogenous Gln-tRNA from which each parental sup-tRNA derives is indicated in parentheses.

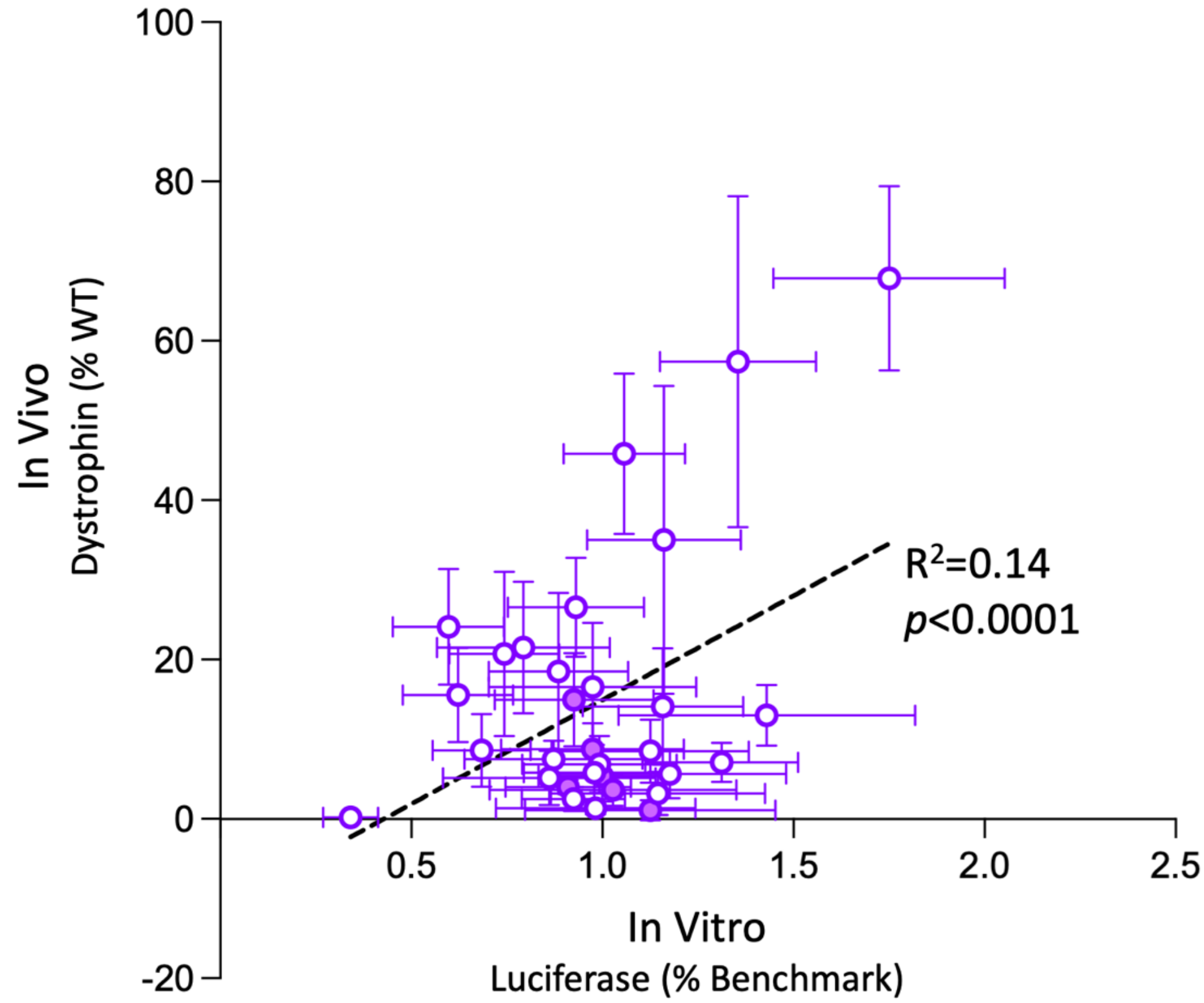

Fig. S5. Correlation between *in vitro* and *in vivo* readthrough efficiency. Results from 31 $tRNA^{Gln}UUA$ candidates that were tested both *in vitro* using a dual Luciferase reporter in HEK293 cells and *in vivo* via intramuscular injection into TA muscles of D2.mdx mice. *In vitro* data are normalized to a benchmark $tRNA^{Gln}UUA$ parental sup-tRNA (tr0157) that was included as an internal control on all plates screened (n=6 or more technical replicates per group). For *in vivo* data, full-length dystrophin was measured by capillary western blot 6 weeks after intramuscular injection of 1×10^{11} vg per muscle of AAV expressing the sup-tRNA. Dystrophin levels in individual animals were normalized to the wild-type average. Error bars indicate standard deviation. Purple shading indicates parental sup-tRNAs.

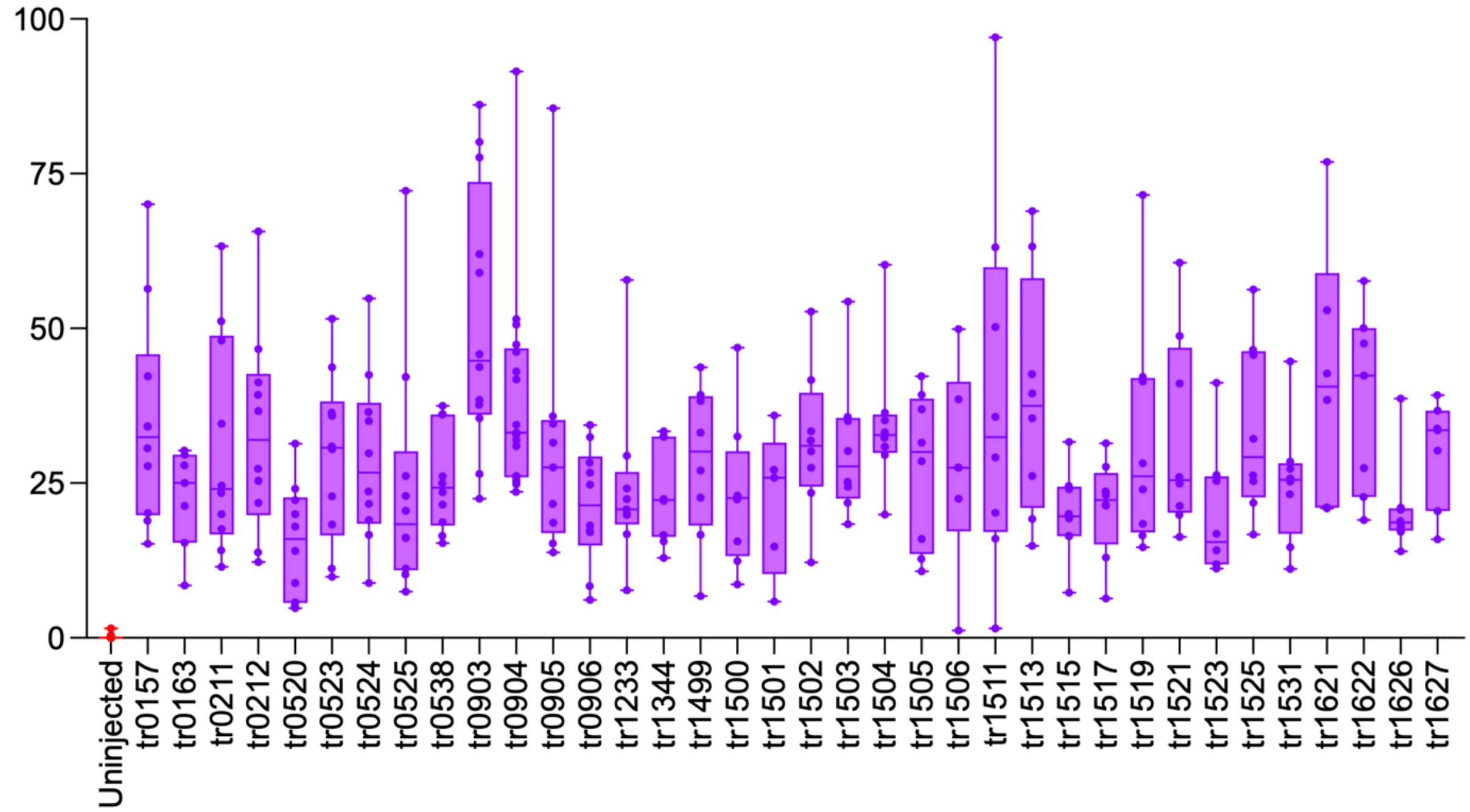

Fig. S6. Biodistribution of sup-tRNA expressing AAVs following IM injection. Efficiency of AAV transduction in TA muscles following intramuscular injection of AAVs expressing the indicated sup-tRNA was calculated by determining viral genome copies relative to diploid genome copies (vg/dg).

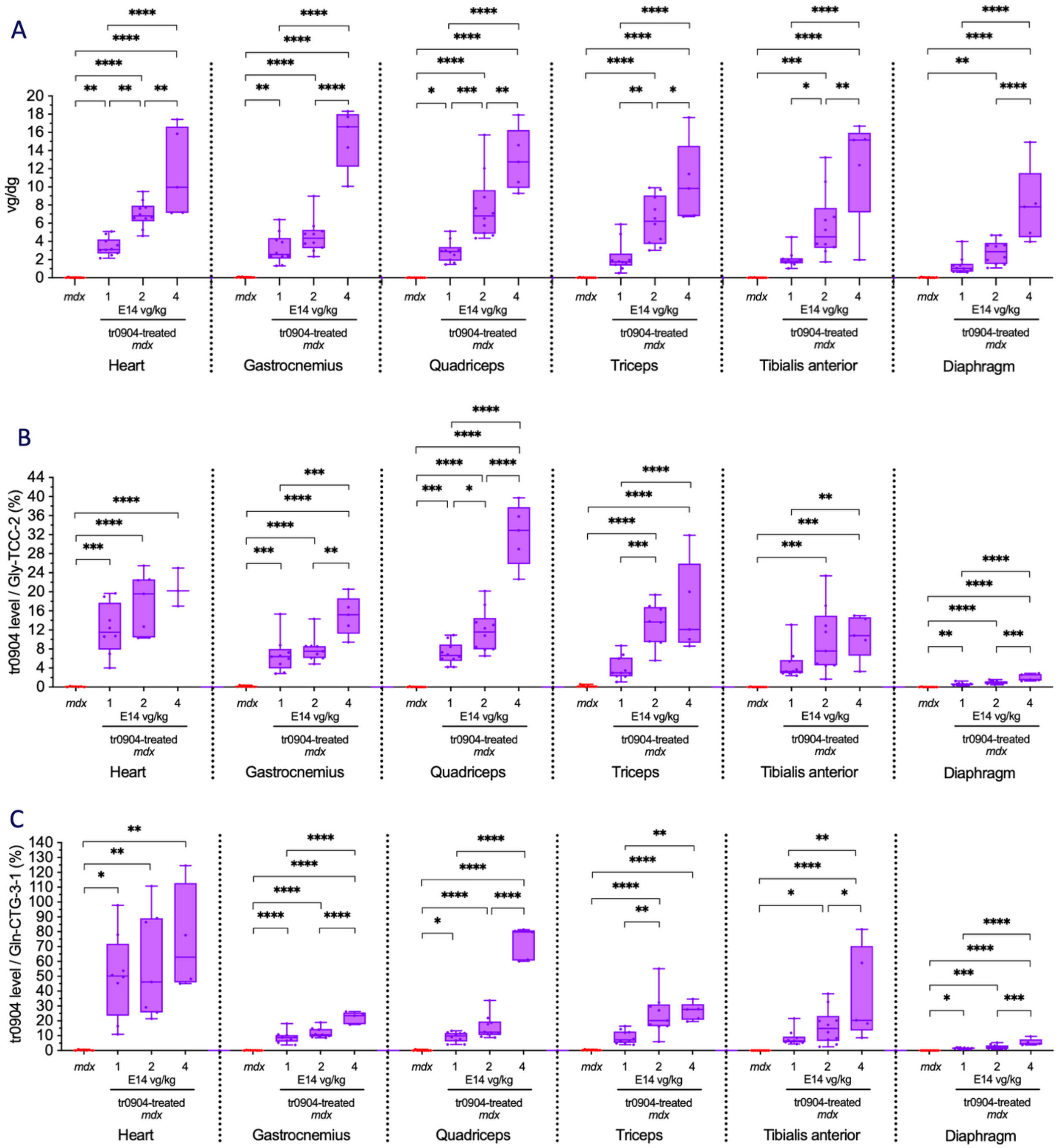

Fig. S7. Quantification of AAV transduction and sup-tRNA expression. (**A**) Viral genome copies normalized to diploid genome copies. (**B, C**) Levels of sup-tRNA in D2.mdx tissues following intravenous injection of AAV-tr0904 at 1, 2, or 4×10[14] vg/kg. tRNA levels were measured by RT-ddPCR 12 weeks after intravenous injection and normalized to (B) the endogenous tRNA-Gly-TCC-2, or (C) the endogenous tRNA-Gln-CTG-3-1. Box plots with individual data points showing middle line as the median; upper and lower quartiles as box

limits; whiskers go down to the smallest value and up to the largest. *<0.05, **<0.005, ***<0.0005, ****<0.00005.

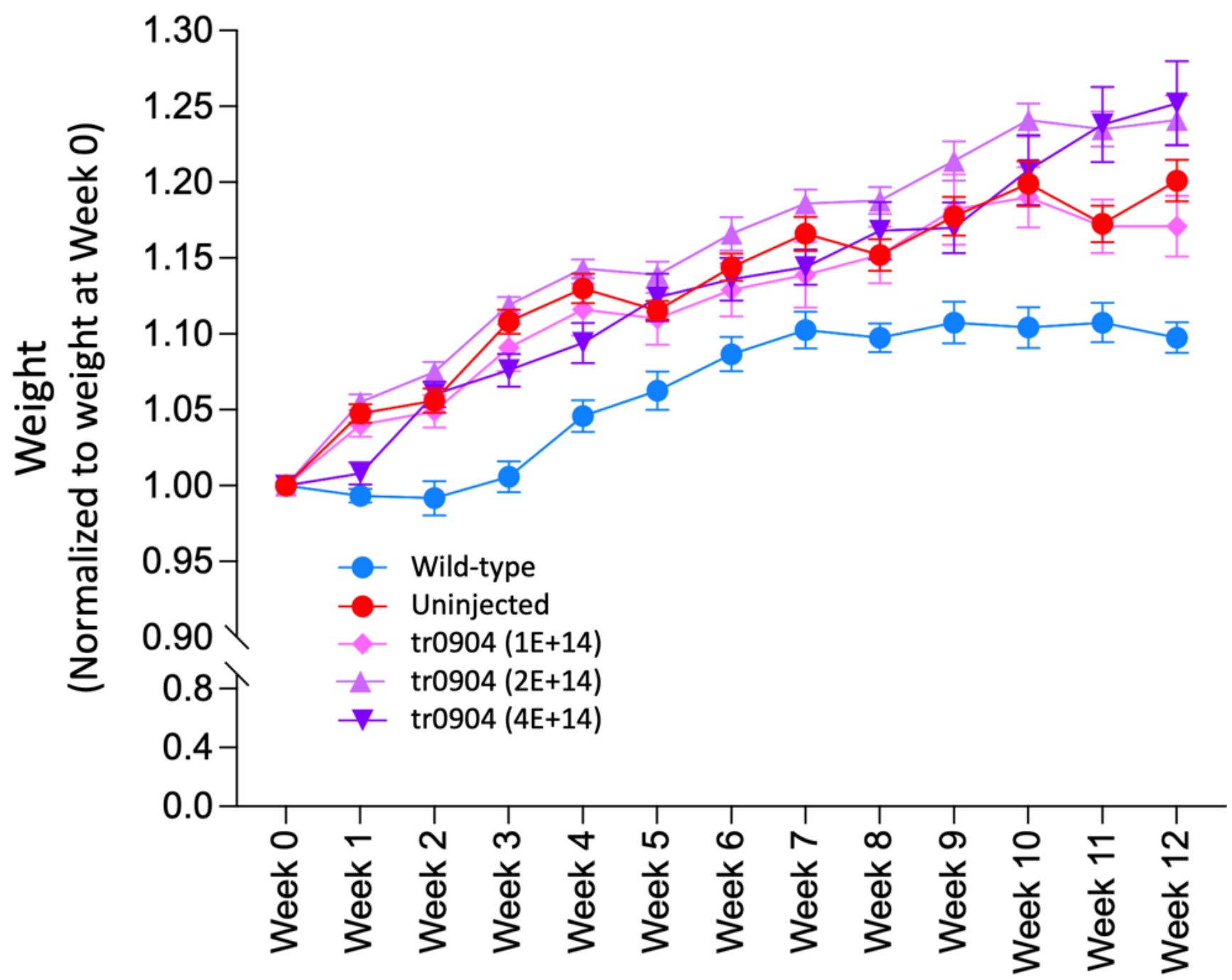

Fig. S8. Treatment with AAV-tr0904 has no impact on mortality or body weight gain in D2.mdx mice. The weight of untreated wild type, untreated D2.mdx, and tr0904-treated D2.mdx mice at 1, 2, or 4×10^{14} vg/kg was recorded weekly over the duration of the study. Plot shows the average weight (± SEM) normalized to weight at study initiation for each group across each timepoint and mean. No animals died over the course of the study.

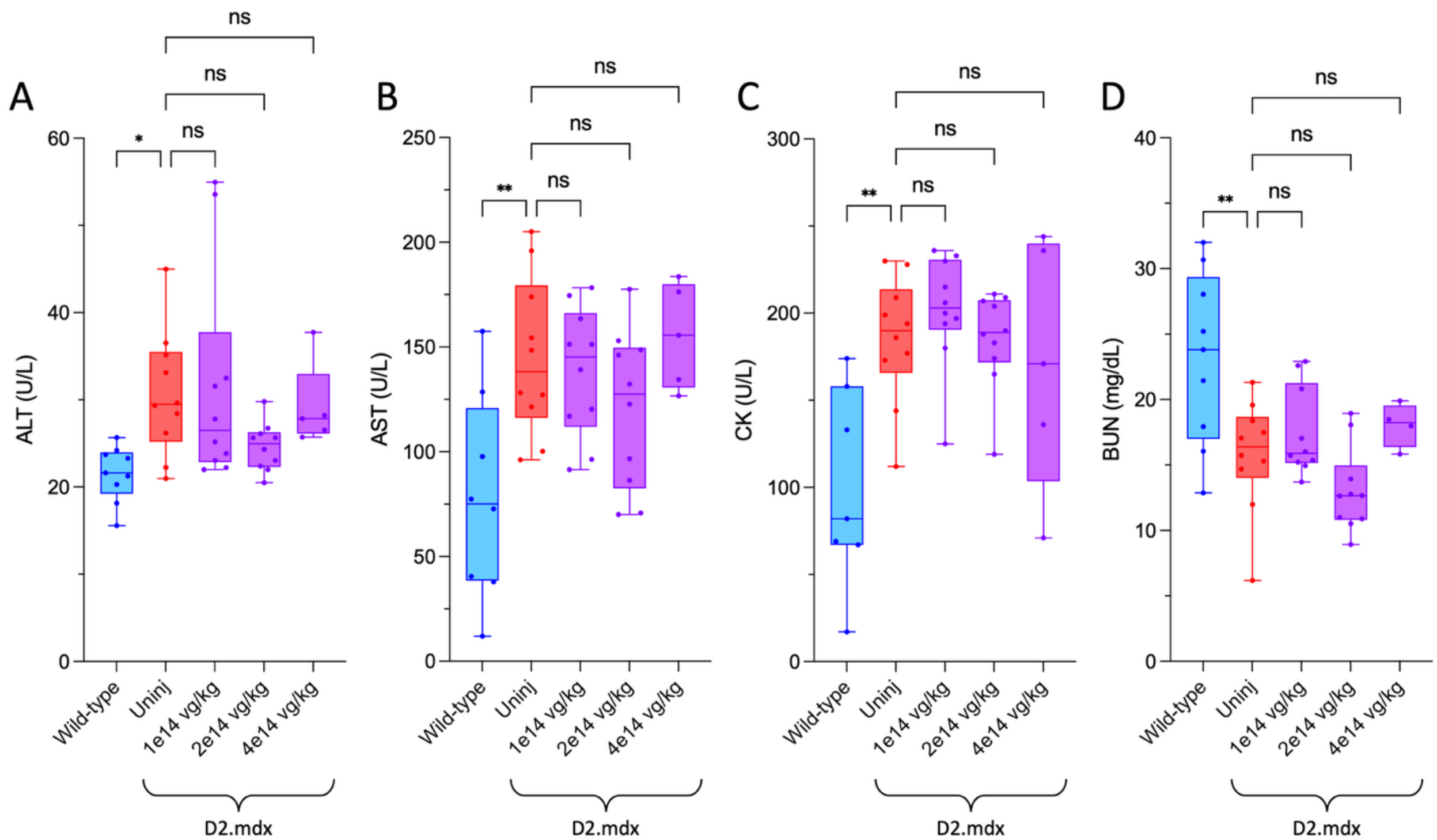

Fig. S9. AAV-tr0904 treatment does not lead to elevation of serum markers of hepatic, muscular, or renal toxicity in D2.mdx mice. (**A-D**) Blood serum was collected at sacrifice (12 weeks after IV injection) and used to determine liver function by measuring alanine aminotransferases (ALT) (A), aspartate aminotransferases (AST) (B), muscle damage by creatine kinase (CK) (C), and renal function by blood urea nitrogen (BUN) (D). Box plots with individual data points showing middle line as the median; upper and lower quartiles as box limits; whiskers go down to the smallest value and up to the largest. Statistical analysis was performed using one-way ANOVA test for uninjected D2.mdx mice versus all groups. *<0.05, **<0.005, ***<0.0005.

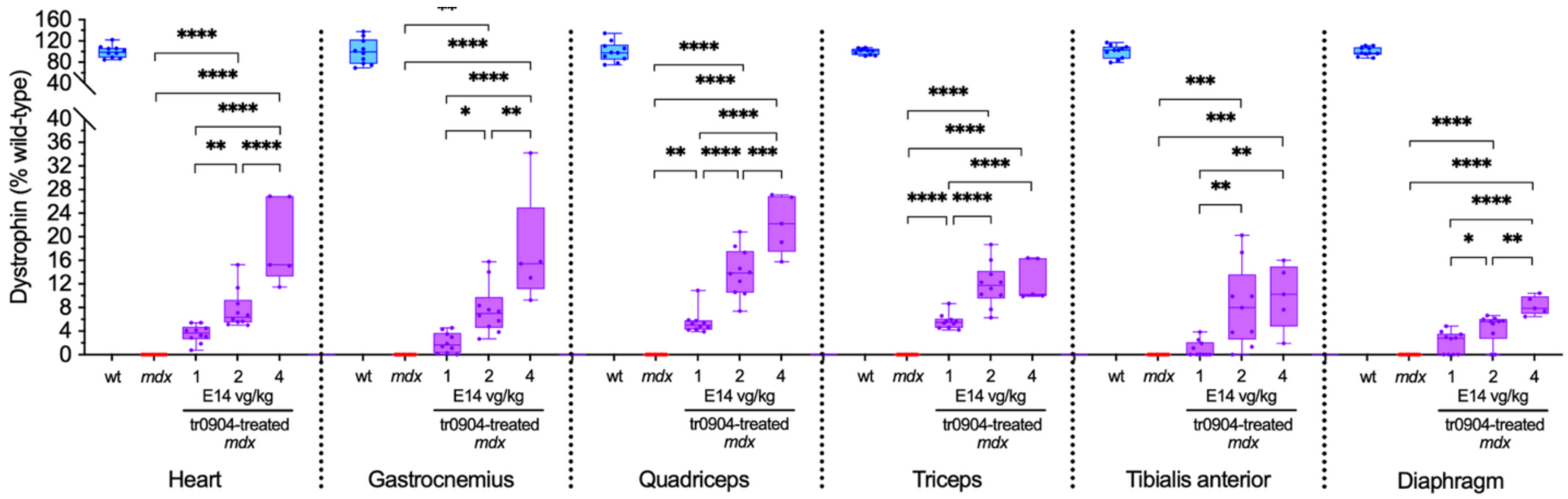

Fig. S10. Quantification of dystrophin rescue by capillary western. Quantification of dystrophin expression in tissues from wild type, D2.mdx, and AAV-tr0904-treated mice at 1, 2, or 4×10^{14} vg/kg (n=5 for 4×10^{14} vg/kg group; n=10 for all other groups). Full-length dystrophin was measured by capillary western blot 12 weeks after intravenous injection. Dystrophin levels were quantified by normalizing dystrophin to total protein signal intensity. Plots show dystrophin levels in individual animals normalized to wild type average. Box plots with individual data points showing middle line as the median; upper and lower quartiles as box limits; whiskers go down to the smallest value and up to the largest. *<0.05, **<0.005, ***<0.0005, ****<0.00005.

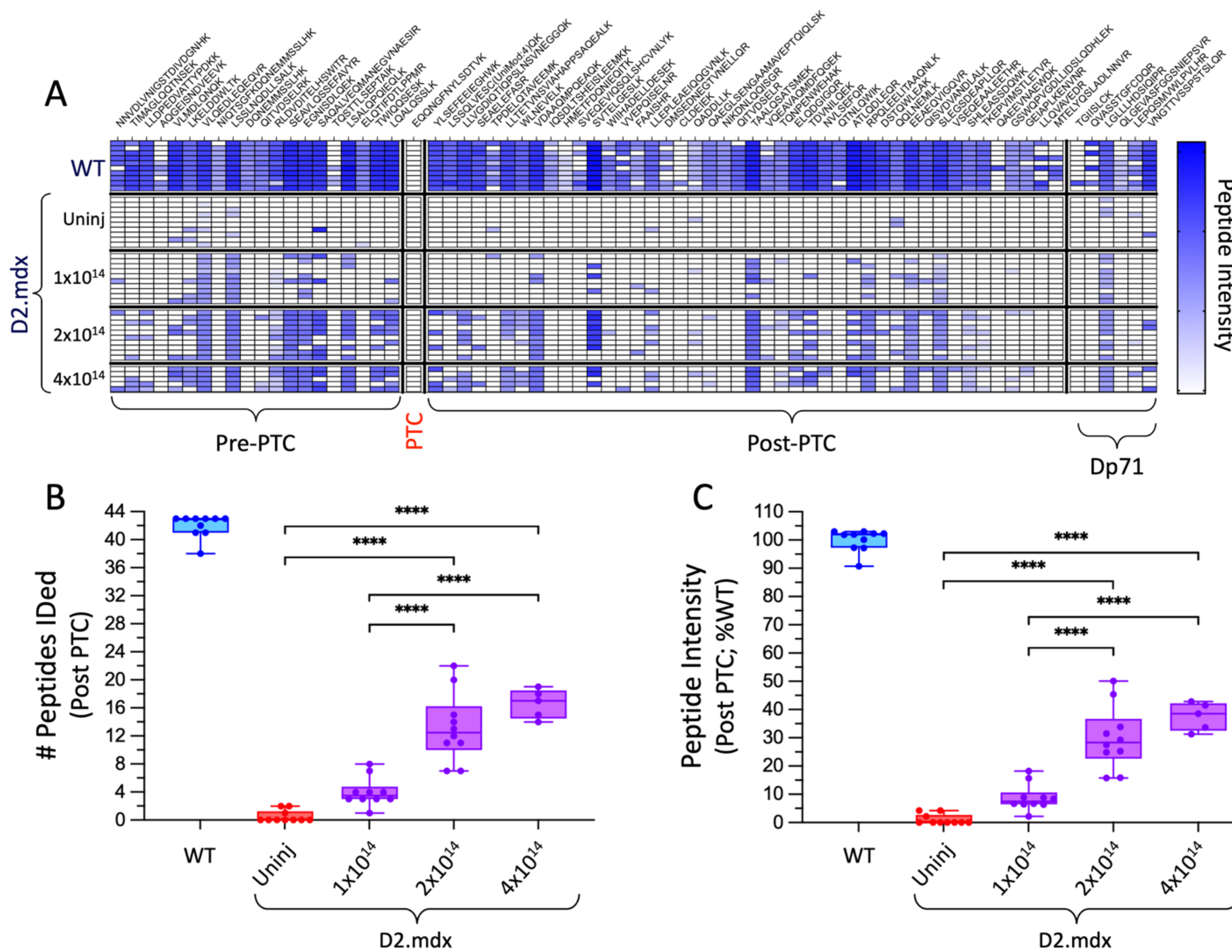

Fig. S11. Quantification of dystrophin rescue by LC-MS/MS. D2.mdx mice were injected intravenously with AAV-tr0904 at 12 weeks of age at 1, 2, or 4×10^{14} vg/kg (n=5 for 4×10^{14} vg/kg group; n=10 for all other groups). Quadriceps muscle was collected 12 weeks later and subjected to quantitative LC-MS/MS. (**A**) Heat map of peptide intensity for all dystrophin peptides quantified in quadriceps muscles. Each row shows data from one animal; each column shows data for the indicated peptide. Peptides are arranged left to right from N-terminus to C-terminus. PTC, peptide containing the premature termination codon; Dp71, peptides common to Dystrophin protein 71 isoform. (**B)** Box plots show the number of peptides (left) and peptide intensity normalized to untreated wild type (right) detected after the premature stop codon (Q995*) by LC-MS/MS. Statistical analysis was performed using one-way ANOVA test for uninjected D2.mdx mice and all sup-tRNA treatment groups. ****<0.00005.

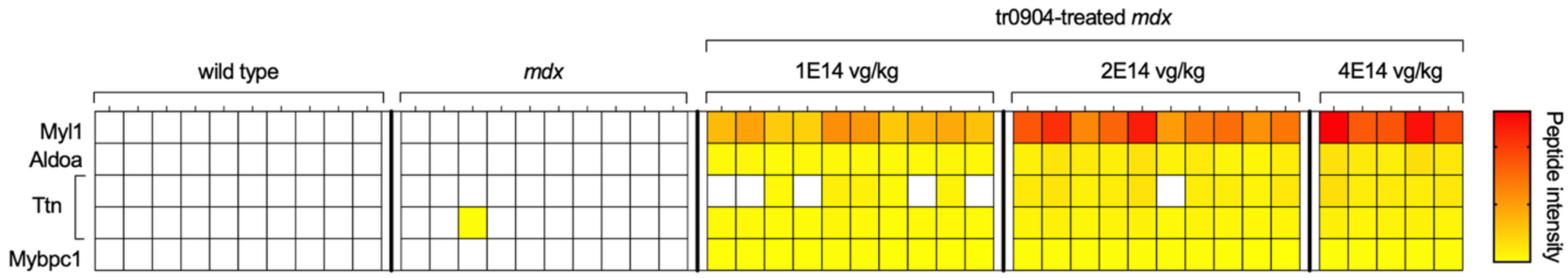

Fig. S12. 3'-UTR extended peptides detected by LC-MS/MS. D2.mdx mice were injected intravenously with AAV-tr0904 at 12 weeks of age at 1, 2, or 4×10^{14} vg/kg (n=5 for 4×10^{14} vg/kg group; n=10 for all other groups). Quadriceps muscle was collected 12 weeks later and subjected to quantitative LC-MS/MS. Heat map of peptide intensity for all 3'-UTR extended peptides detected in quadriceps muscles. Each column shows data from one animal; each row shows data for the indicated peptide. Shading reflects peptide intensity from 1 to the peak value detected (5.21×10^6); white indicates the peptide was not detected in the sample. 3'-UTR extended peptide sequences:

Myl1a, HIMSVQTEFSSTQC(UniMod:4)WGR

Aldoa, YTPSGQSGAAASESLFISNHAYQPELN

Ttn peptide 1, GPFTLDAHS

Ttn peptide 2, SMQEGQGQER

Mybpc1, WAVLLQAPPAR

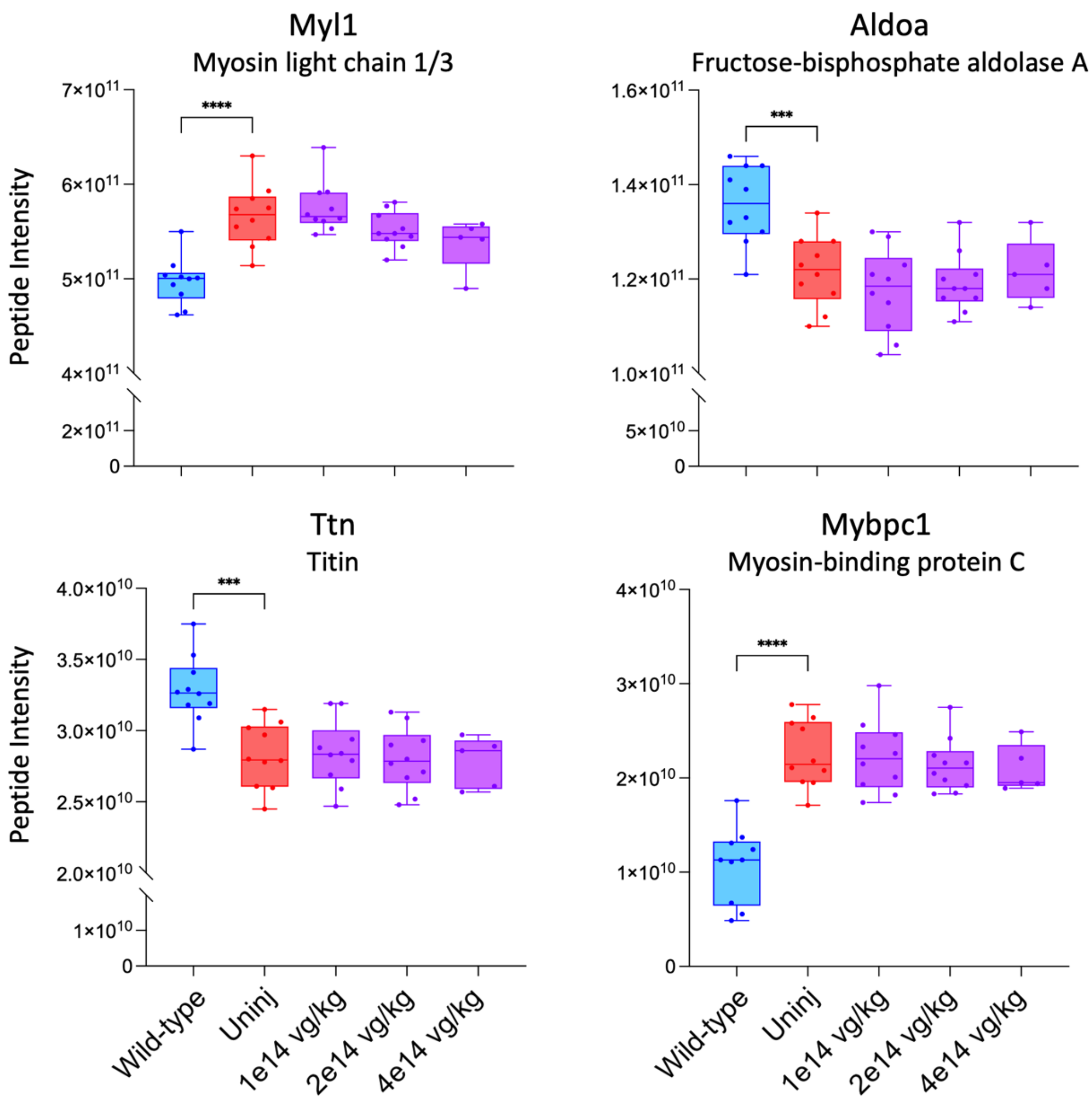

Fig. S13. Expression of proteins with detectable 3'-UTR extended peptides. Box plots showing expression of proteins associated with detectable 3'-UTR extended peptides from quadriceps muscles of 24-week-old D2.mdx mice as determined by quantitative LC-MS/MS. D2.mdx mice were injected intravenously with AAV-tr0904 at the indicated doses at 12 weeks of age. Box plots with individual data points showing middle line as the median; upper and lower quartiles as box limits; whiskers go down to the smallest value and up to the largest. Statistical analysis was performed using one-way ANOVA test for uninjected D2.mdx mice versus all sup-tRNA treatment groups. ***<0.0005, ****<0.00005.

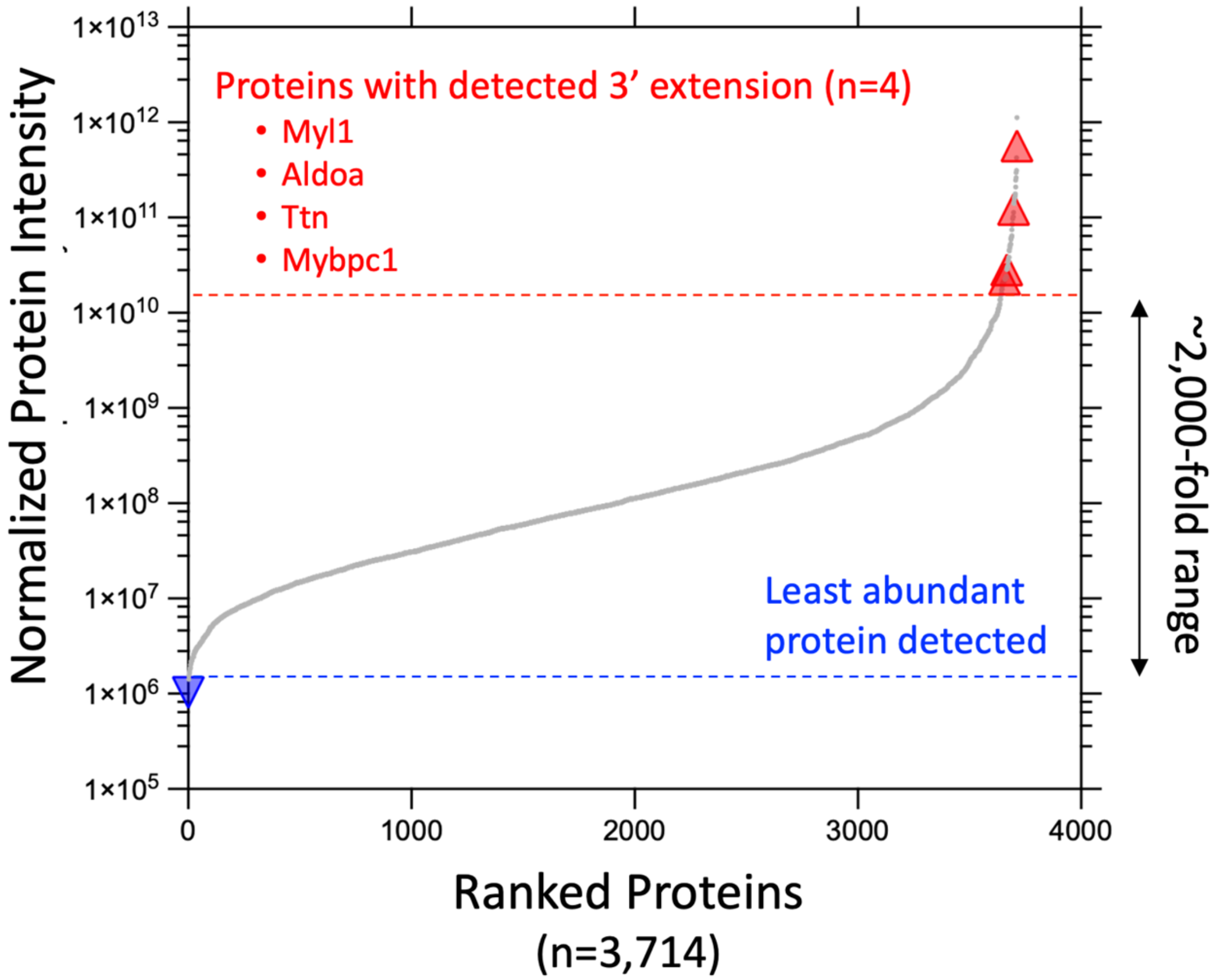

Fig. S14. AAV-tr0904 does not promote native stop codon readthrough at doses sufficient to reverse DMD-associated proteome dysregulation *in vivo*. D2.mdx mice were injected intravenously with AAV-tr0904 at 12 weeks of age at 1, 2, or $4×10^{14}$ vg/kg (n=5 for $4×10^{14}$ vg/kg group; n=10 for all other groups). Quadriceps muscle was collected 12 weeks later and subjected to quantitative LC-MS/MS. Plot of all proteins detected by LC-MS/MS global proteomics analysis in quadriceps muscles of untreated D2.mdx mice. 3'-UTR extension products were only detected from four proteins (red) present at levels more than $2×10^3$ above the lower limit of detection established by the least abundant identified proteins (blue).

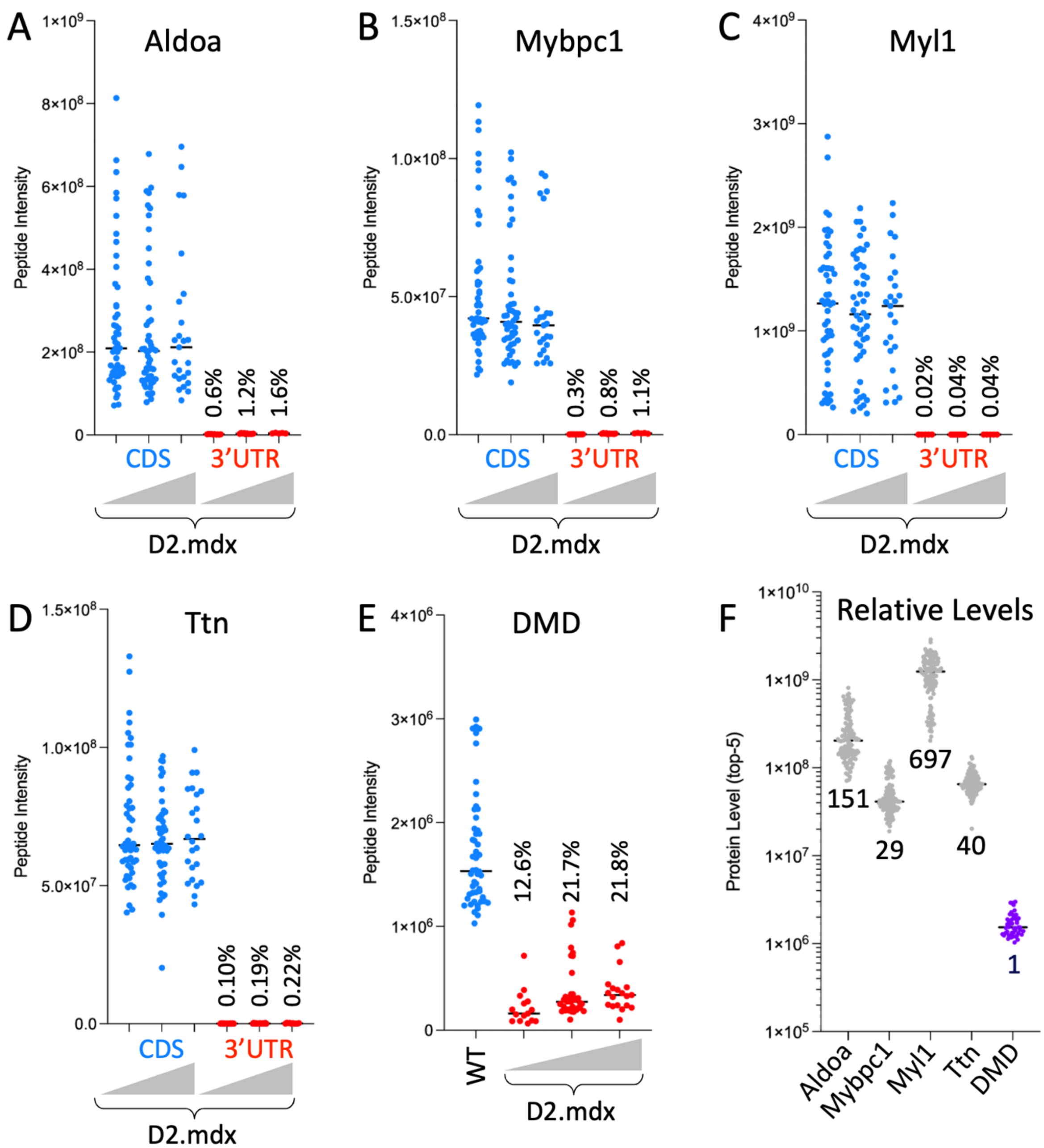

Fig. S15. AAV-tr0904 shows high selectivity for nonsense mutations over native stop codons *in vivo*. D2.mdx mice were injected intravenously with AAV-tr0904 at 12 weeks of age. Quadriceps muscle was collected 12 weeks later and subjected to quantitative LC-MS/MS to detect off-target readthgouh. **(A-D)** Scatter plots of off-target LC-MS/MS analysis from all four proteins with detectable 3'-UTR extended peptides. Plots show raw peptide intensities of detected 3'-UTR extended peptides (3'UTR; red dots) relative to peptide intensities of top-5 detected tryptic peptides from the coding region of each associated protein (CDS; blue dots). All data are from D2.mdx mice injected with increasing doses of AAV-tr0904 (dose range indicated by gray triangles; 1, 2, or 4×10^{14} vg/kg; n=5 for 4×10^{14} vg/kg

group; n=10 for all other groups). **(E)** Scatter plot from the same analysis showing raw peptide intensities of top-5 detected tryptic peptides from the coding region of dystrophin (DMD) in wild-type mice (WT) and D2.mdx mice treated with increasing doses of AAV-tr0904 (dose range indicated by gray triangles). **(F)** Scatter plot from the same analysis showing levels of dystrophin (DMD; purple dots) in quadriceps muscle relative to all four proteins with detectable 3'-UTR extended peptides (gray dots). Data include peptide intensities of top-5 detected tryptic coding region peptides from Aldoa, Mybpc1, Myl1 and Ttn (all cohorts) and from DMD (wild-type cohort only).

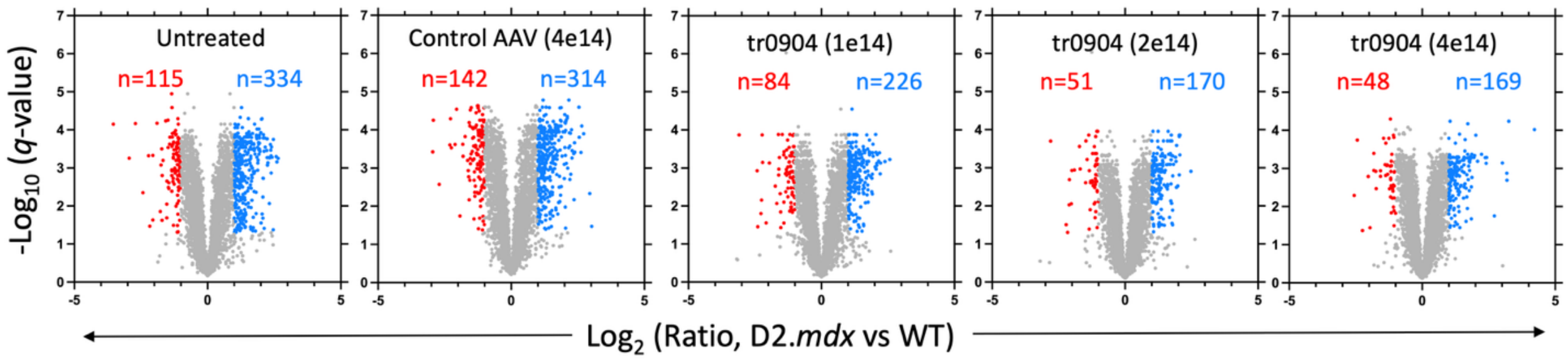

Fig. S16. Analysis of differentially expressed proteins in quadriceps muscles as determined by LC-MS/MS. D2.mdx mice were injected intravenously with AAV-tr0904 at 12 weeks of age at 1, 2, or 4×10^{14} vg/kg (n=5 for 4×10^{14} vg/kg group; n=10 for all other groups). Quadriceps muscle was collected 12 weeks later and subjected to quantitative LC-MS/MS. Cohorts with more than 5 mice were downsampled to n=5 and differential expression relative to wild-type (WT) controls was assessed for each D2.mdx cohort (unpaired t tests, two-stage step-up method of Benjamini, Krieger and Yekutieli, adjusted P<0.05). Volcano plots depict the number of proteins differentially expressed by more than two-fold ($\log_2 = \pm 1$) relative to WT. Gray, proteins having a fold-change less than 2 ($\log_2 = 1$) and/or an adjusted P value greater than 0.05; red, proteins that are downregulated; blue, proteins that are upregulated.

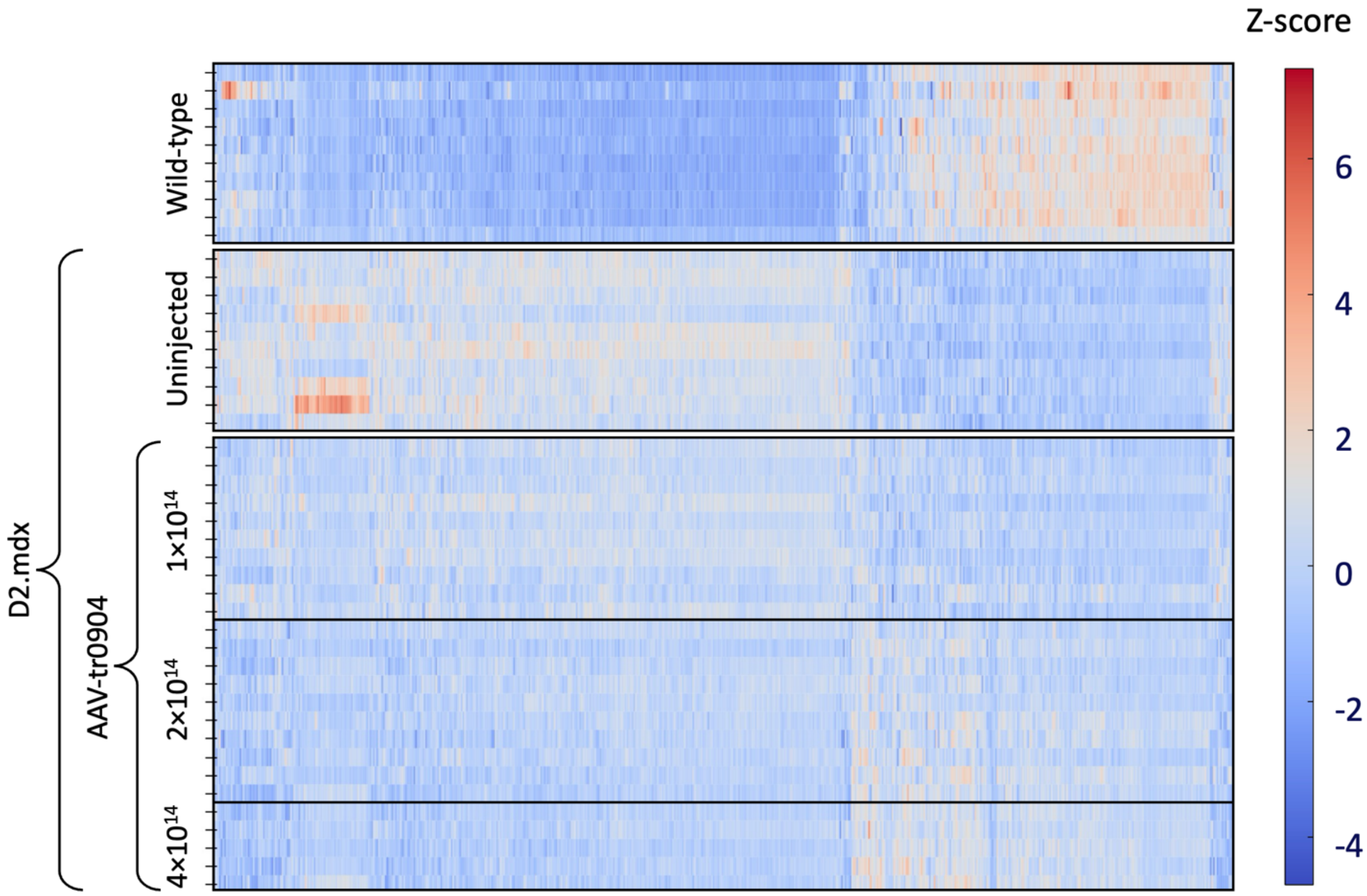

Fig. S17. Analysis of differentially expressed proteins in D2.mdx mice following suppressor tRNA treatment. One-way ANOVA was performed to identify significant differences between untreated D2.mdx mice and cohorts treated with AAV-tr0904 at 1, 2, or 4×10^{14} vg/kg (adjusted *P*<0.05; Benjamini-Hochberg correction). Proteins that were significantly different in at least one inter-cohort comparison (n=603) were visualized by heatmap. Each cell is color-coded, with color intensity reflecting the level of protein abundance (by *z*-score)—Red indicate higher while blue indicate lower abundance.

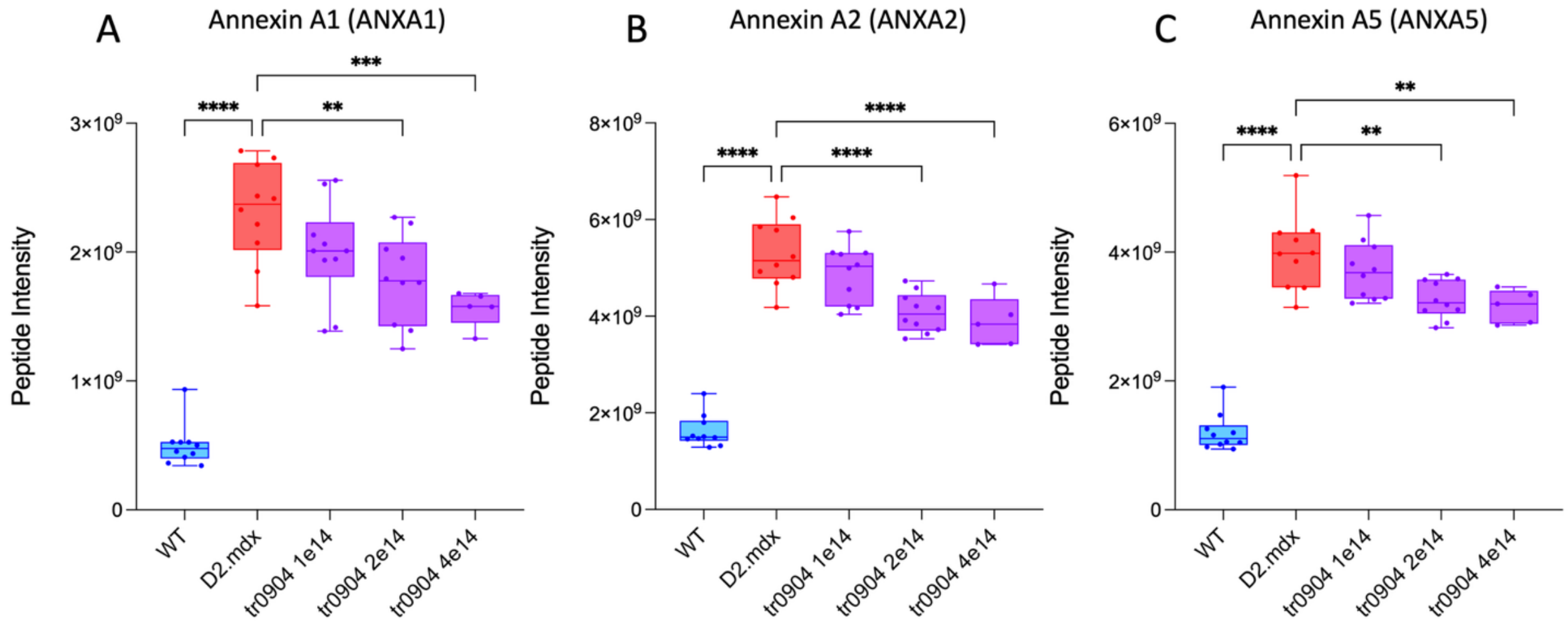

Fig. S18. Restoration of differentially expressed proteins associated with membrane repair (annexin A family). (A-C) Box plots showing changes in annexin A family members from quadriceps muscles of 24-week-old WT and D2.mdx mice was determined by quantitative LC-MS/MS. D2.mdx mice were injected intravenously with AAV-tr0904 (tr0904) at 1, 2, or 4×10^{14} vg/kg (1e14, 2e14, 4e14) at 12 weeks of age. (A) Annexin A1 (Anxa1; UniProt P10107). (B) Annexin A2 (Anxa2; UniProt P07356). (C) Annexin A5 (Anxa5; UniProt P48036). Significance relative to untreated D2.mdx cohort determined by one-way ANOVA (Dunnett adjusted P<0.05). Box plots with individual data points showing middle line as the median; upper and lower quartiles as box limits; whiskers go down to the smallest value and up to the largest. **<0.005, ***<0.0005, ****<0.00005.

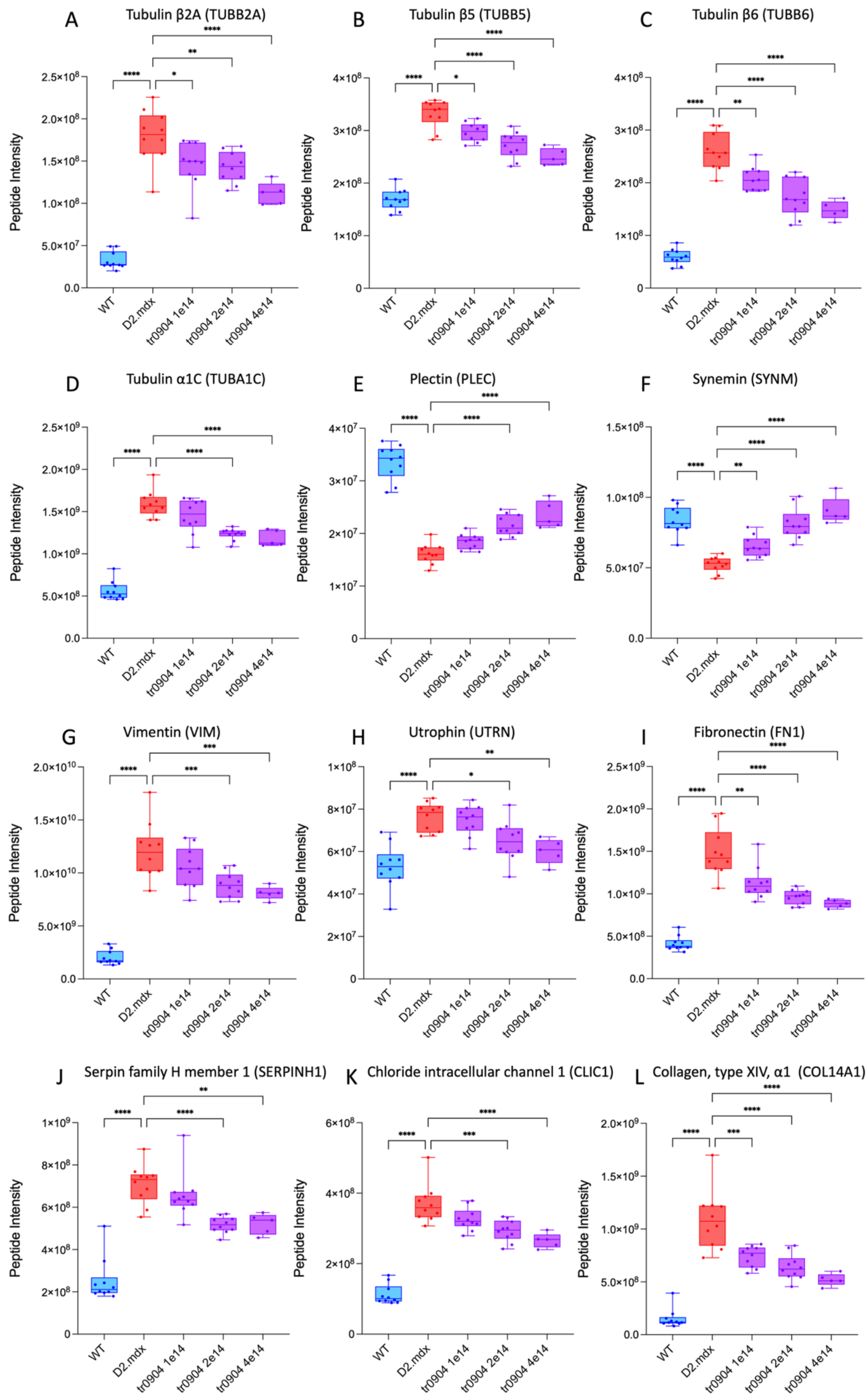
A
Tubulin β2A (TUBB2A)
B
Tubulin β5 (TUBB5)
C
Tubulin β6 (TUBB6)
D
Tubulin α1C (TUBA1C)
E
Plectin (PLEC)
F
Synemin (SYNM)
G
Vimentin (VIM)
H
Utrophin (UTRN)
I
Fibronectin (FN1)
J
Serpin family H member 1 (SERPINH1)
K
Chloride intracellular channel 1 (CLIC1)
L
Collagen, type XIV, α1 (COL14A1)
Peptide Intensity
WT
D2.mdx
tr0904 1e14
tr0904 2e14
tr0904 4e14

Fig. S19. Restoration of differentially expressed proteins associated with the cytoskeleton and dystrophin network. (A-G) Box plots showing changes in proteins associated with the cytoskeleton and dystrophin network from quadriceps muscles of 24-week-old WT and D2.mdx mice as determined by quantitative LC-MS/MS. D2.mdx mice were injected intravenously with AAV-tr0904 (tr0904) at 1, 2, or 4×10^{14} vg/kg (1e14, 2e14, 4e14) at 12 weeks of age. (A-D) members of the tubulin superfamily, including tubulin beta-2A chain (Tubb2a; UniProt Q7TMM9)(A), tubulin beta-5 chain (Tubb5; UniProt P99024)(B), tubulin beta-6 chain (Tubb6; UniProtQ922F4)(C), and tubulin alpha-1C chain (Tuba1c; UniProt P68373)(D). (E) Plectin (Plec; UniProt Q9QXS1). (F) Synemin (Synm; UniProt Q70IV5). (G) Vimentin (Vim; UniProt P20152). (H) Utrophin (Utrn; UniProt E9Q6R7). (I) Fibronectin (Fn1; UniProt A04087WR50). (J) Serpin family H member 1 (Serpinh1; UniProt P19324). (K) Chloride intracellular channel 1 (Clic1; UniProt Q9Z1Q5). (L) Collagen, type XIV, alpha-1 (Col14A1; UniProt Q80X19). Significance relative to untreated D2.mdx cohort determined by one-way ANOVA (Dunnett adjusted P<0.05). Box plots with individual data points showing middle line as the median; upper and lower quartiles as box limits; whiskers go down to the smallest value and up to the largest. *<0.05, **<0.005, ***<0.0005, ****<0.00005.

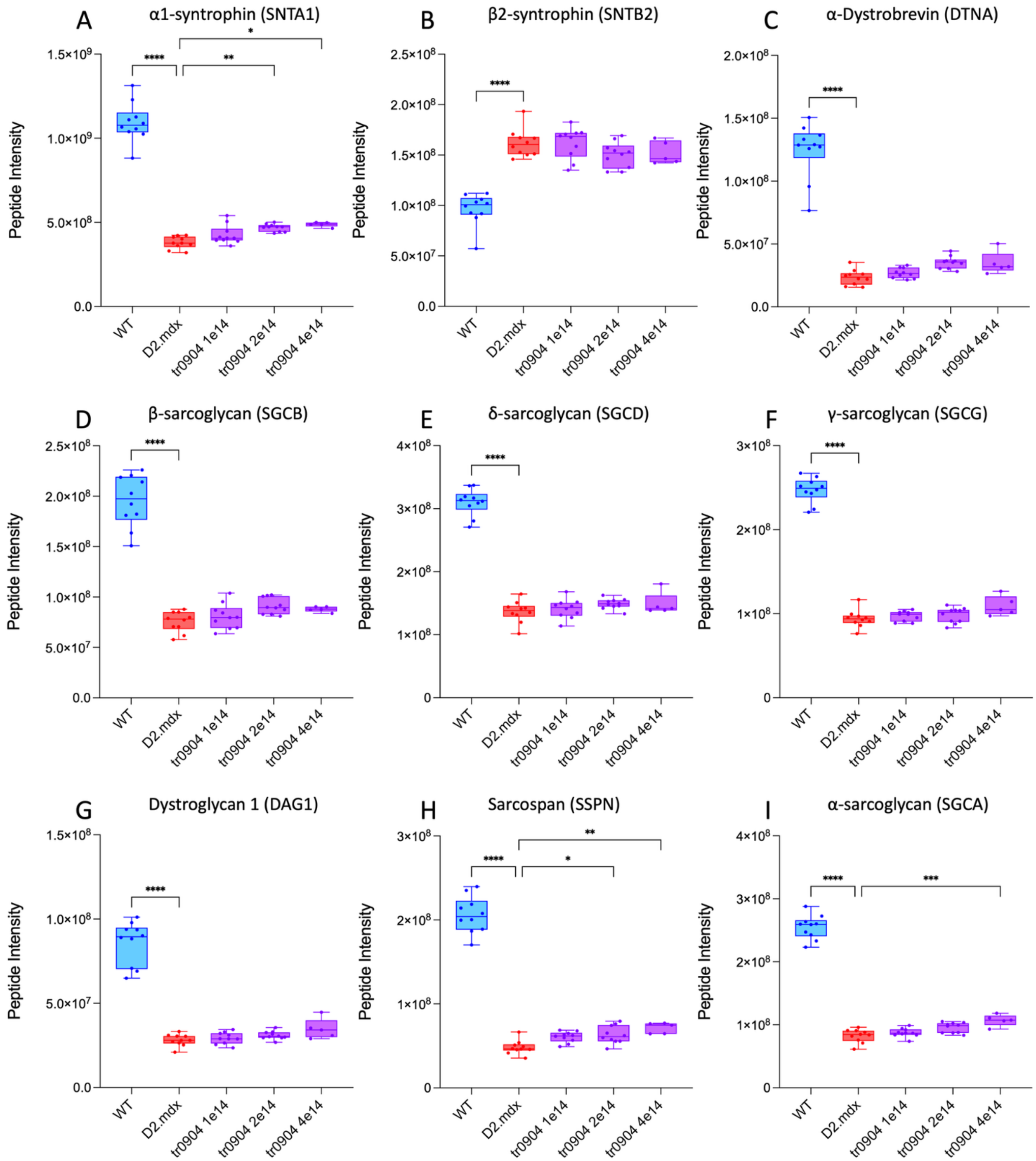

Fig. S20. Restoration of differentially expressed proteins associated with the dystrophin-glycoprotein complex (DGC). (A-I) Box plots showing changes in proteins associated with the dystrophin-glycoprotein complex from quadriceps muscles of 24-week-old WT and D2.mdx mice as determined by quantitative LC-MS/MS. D2.mdx mice were injected intravenously with AAV-tr0904 (tr0904) at 1, 2, or 4×10^{14} vg/kg (1e14, 2e14, 4e14) at 12 weeks of age. **(A)** Alpha-1-syntrophin (Snta1; UniProt Q61234). **(B)** Beta-2-syntrophin

(Sntb2; UniProt Q61235). **(C)** Dystrobrevin alpha (Dtna; UniProt Q9D2N4). **(D)** Beta-sarcoglycan (Sgcb; UniProt P82349). **(E)** Delta-sarcoglycan (Sgcd; UniProt P82347). **(F)** Gamma-sarcoglycan (Sgcg; UniProt P82348). **(G)** Dystroglycan 1 (Dag1; UniProt Q62165). **(H)** Sarcospan (Sspn; UniProt Q62147). **(I)** Alpha-sarcoglycan (Sgca; UniProt P82350). Significance relative to untreated D2.mdx cohort determined by one-way ANOVA (Dunnett adjusted $P<0.05$). Box plots with individual data points showing middle line as the median; upper and lower quartiles as box limits; whiskers go down to the smallest value and up to the largest. *<0.05, **<0.005, ***<0.0005, ****<0.00005.

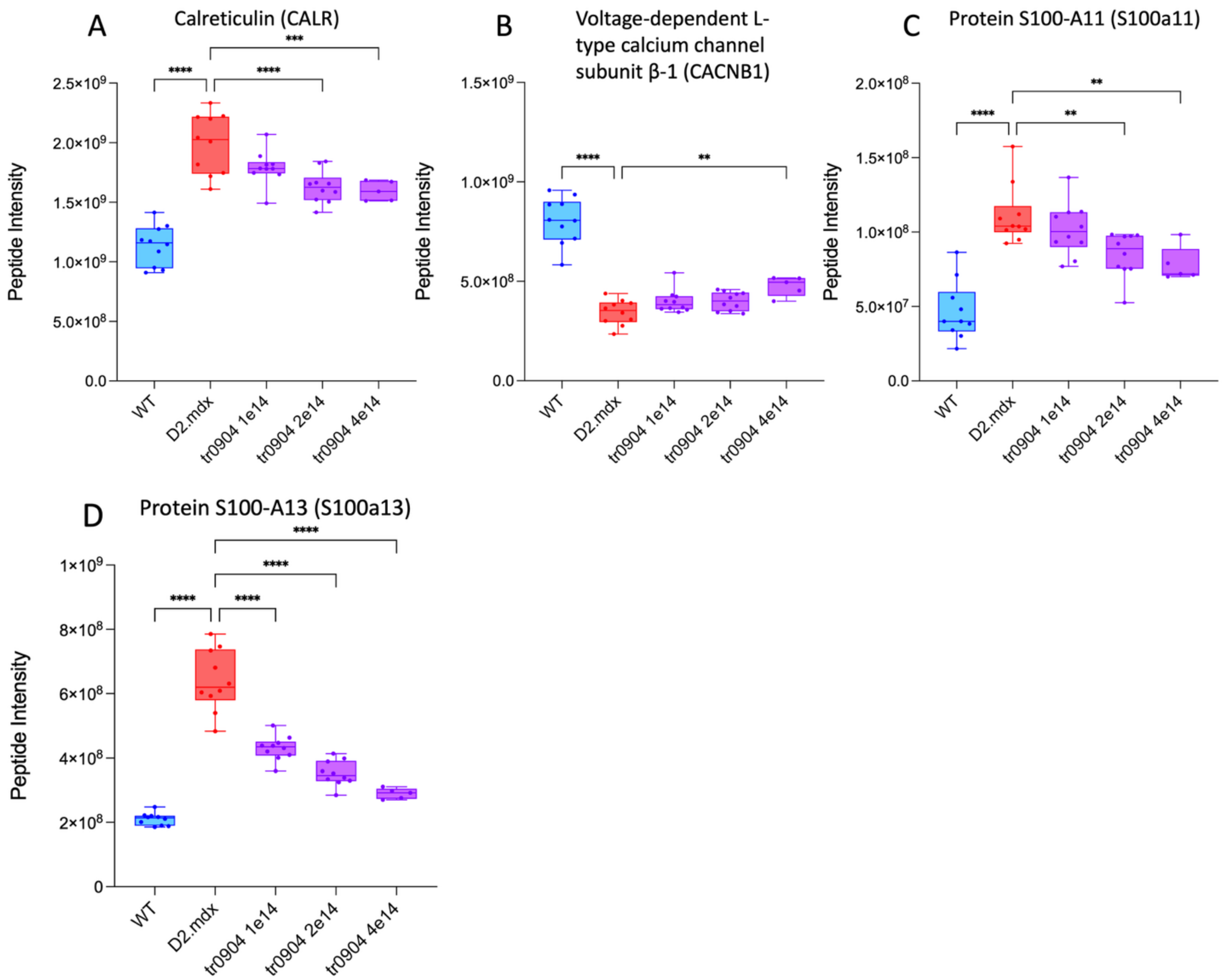

Fig. S21. Restoration of differentially expressed proteins associated with calcium binding and handling. (A-D) Box plots showing changes in proteins associated with calcium binding and handling from quadriceps muscles of 24-week-old WT and D2.mdx mice as determined by quantitative LC-MS/MS. D2.mdx mice were injected intravenously with AAV-tr0904 (tr0904) at 1, 2, or 4×10^{14} vg/kg (1e14, 2e14, 4e14) at 12 weeks of age. **(A)** Calreticulin (Calr; UniProt P14211). **(B)** Voltage-dependent L-type calcium channel subunit beta-1 (Cacnb1; UniProt Q8R3Z5). **(C)** Protein S100-A11 (S100a11; UniProt P50543). **(D)** Protein S100-A13 (S100a13; UniProt P97352). Significance relative to untreated D2.mdx cohort determined by one-way ANOVA (Dunnett adjusted P<0.05). Box plots with individual data points showing middle line as the median; upper and lower quartiles as box limits; whiskers go down to the smallest value and up to the largest. **<0.005, ***<0.0005, ****<0.00005.

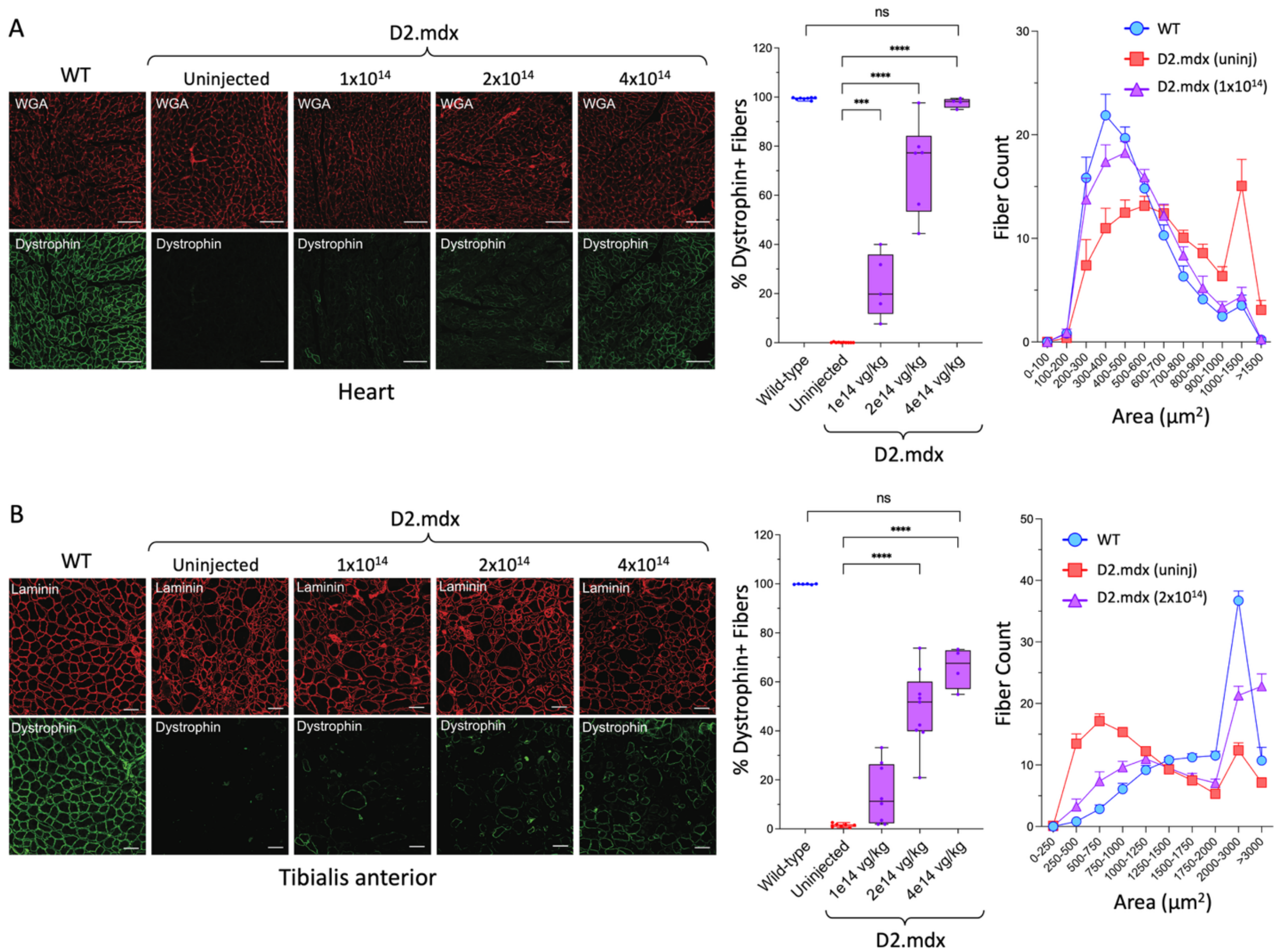

Fig. S22. Pathophysiology and motor function are rescued in dystrophic mice following systemic delivery of sup-tRNAs. (A) Representative immunofluorescence staining of dystrophin (green) and muscle fiber boundaries labeled with wheat germ agglutinin (red, WGA) in heart muscle from wild type, D2.mdx, and tr0904-treated mice (left). Quantification of dystrophin positive muscle fibers in heart muscle (center). Muscle fiber size distribution (mm^2) in heart muscle from wild type, D2.mdx, and tr0904-treated mice (2×10^{14} vg/kg; right). **(B)** Representative immunofluorescence staining of dystrophin (green) and muscle fiber boundaries labeled with laminin (red) in TA muscle from wild type, D2.mdx, and tr0904-treated mice (left). Quantification of dystrophin positive muscle fibers (center). Muscle fiber size distribution (mm^2) in TA muscle from wild type, D2.mdx, and tr0904-treated mice (2×10^{14} vg/kg; right). (A, B) Muscle fiber size plots show mean ± SEM normalized to total numbers of fibers (%). Significance relative to untreated D2.mdx cohort determined by one-way ANOVA (Dunnett adjusted $P<0.05$). Box plots with individual data points showing middle line as the median; upper and lower quartiles as box limits; whiskers go down to the smallest value and up to the largest. ***<0.0005, ****<0.00005.

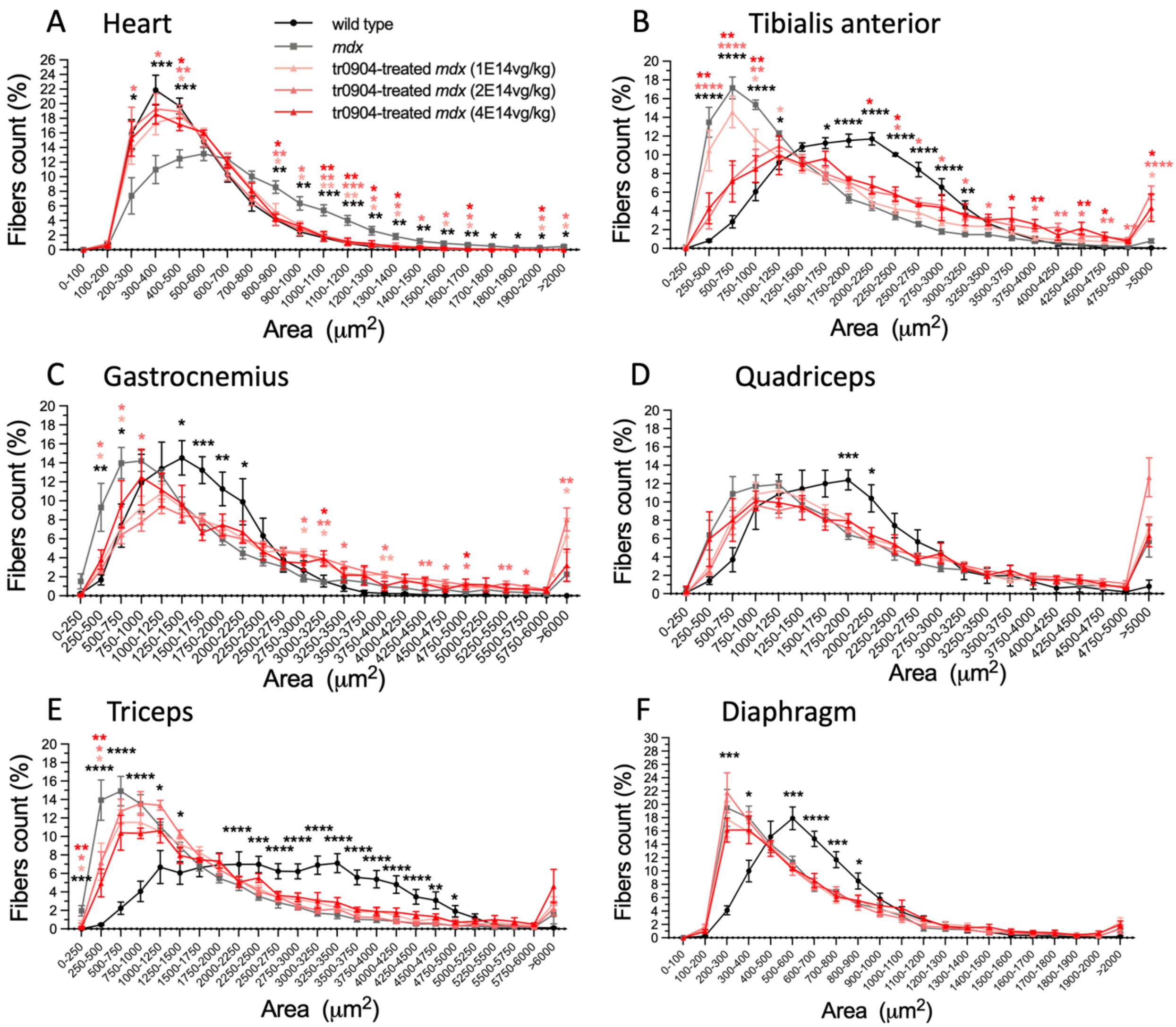

Fig. S23. Analysis of muscle fiber size in skeletal muscle of dystrophic mice following systemic delivery of AAV-tr0904. (A) Heart, **(B)** tibialis anterior, **(C)** gastrocnemius, **(D)** quadriceps, **(E)** triceps, and **(F)** diaphragm muscle fiber size distribution (mm^2) of wild type, D2.mdx (mdx), and tr0904-treated mice at 1, 2, or 4×10^{14} vg/kg (1E14, 2E14, 4E14). Plots show mean ± SEM normalized to total numbers of fibers (%). Statistical analysis was performed using one-way ANOVA test comparing each group to untreated D2.*mdx* group. *<0.05, **<0.005, ***<0.0005, ****<0.00005.

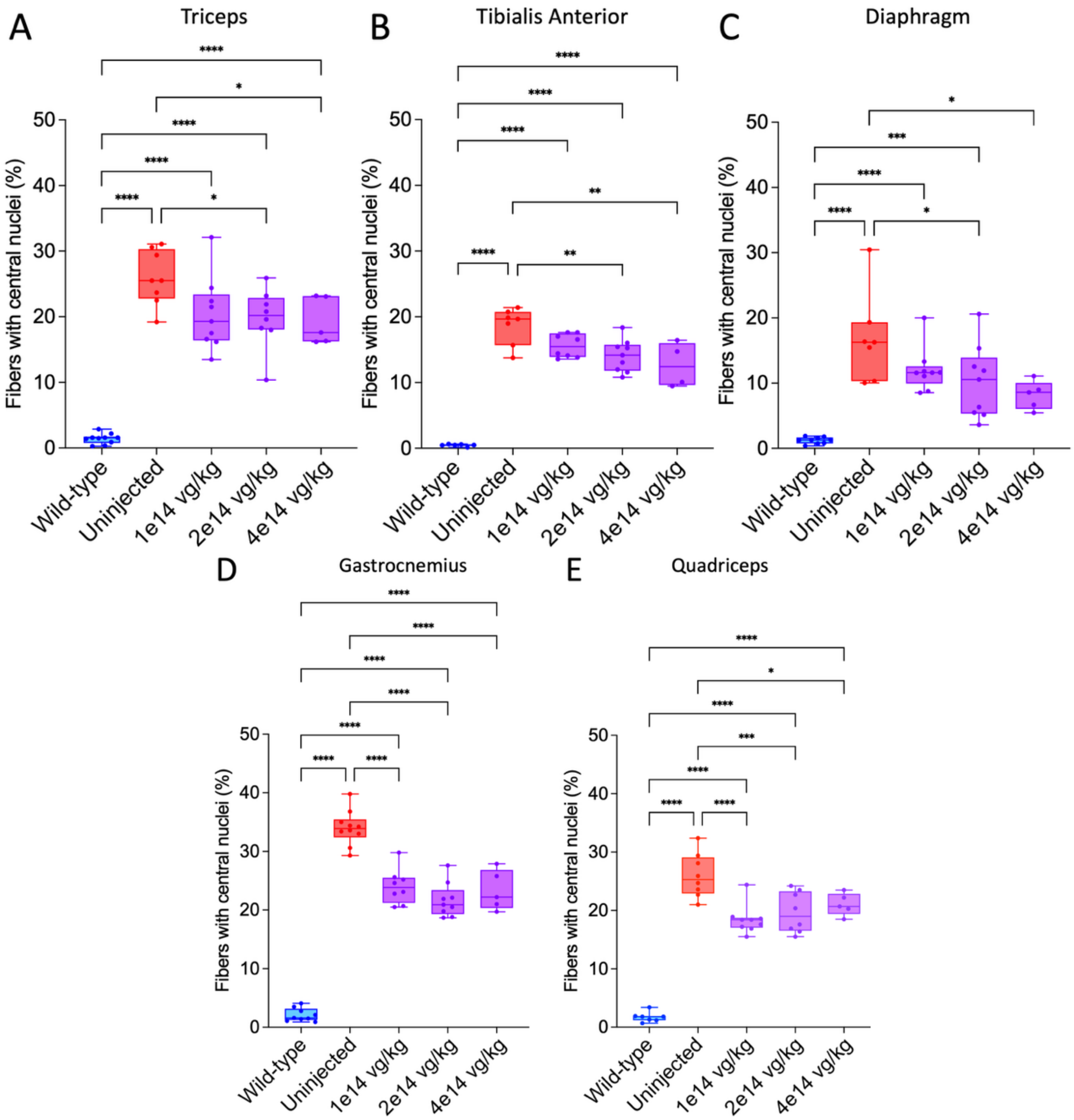

Fig. S24. Analysis of central nuclei in skeletal muscle of dystrophic mice following systemic delivery of AAV-tr0904. Quantification of muscle fibers with central nuclei in **(A)** triceps, **(B)** tibialis anterior, **(C)** diaphragm, **(D)** gastrocnemius, and (**E**) quadriceps muscles. Box plots with individual data points showing middle line as the median; upper and lower quartiles as box limits; whiskers go down to the smallest value and up to the largest. *<0.05, ***<0.0005, ****<0.00005.

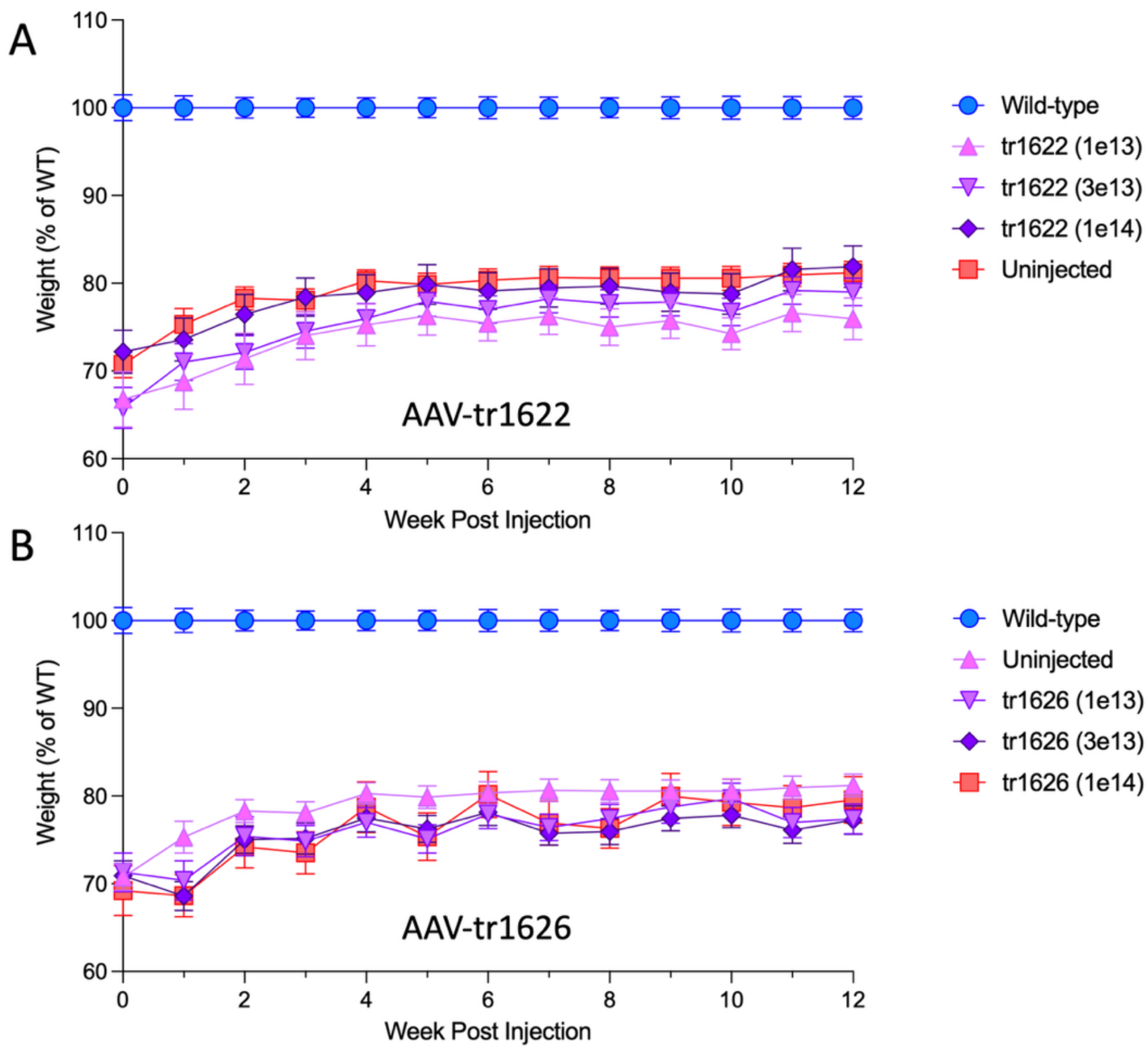

Fig. S25. Treatment with AAV-tr1622 and AAV-tr1626 has no impact on mortality or body weight gain in D2.mdx mice. The weight of untreated wild type, untreated D2.mdx, and tr0904-treated D2.mdx mice at 1×10^{13}, 3×10^{13}, or 1×10^{14} vg/kg was recorded weekly over the duration of the study. Plot shows average weights (± SEM) normalized to wild-type average. No animals died over the course of the study.

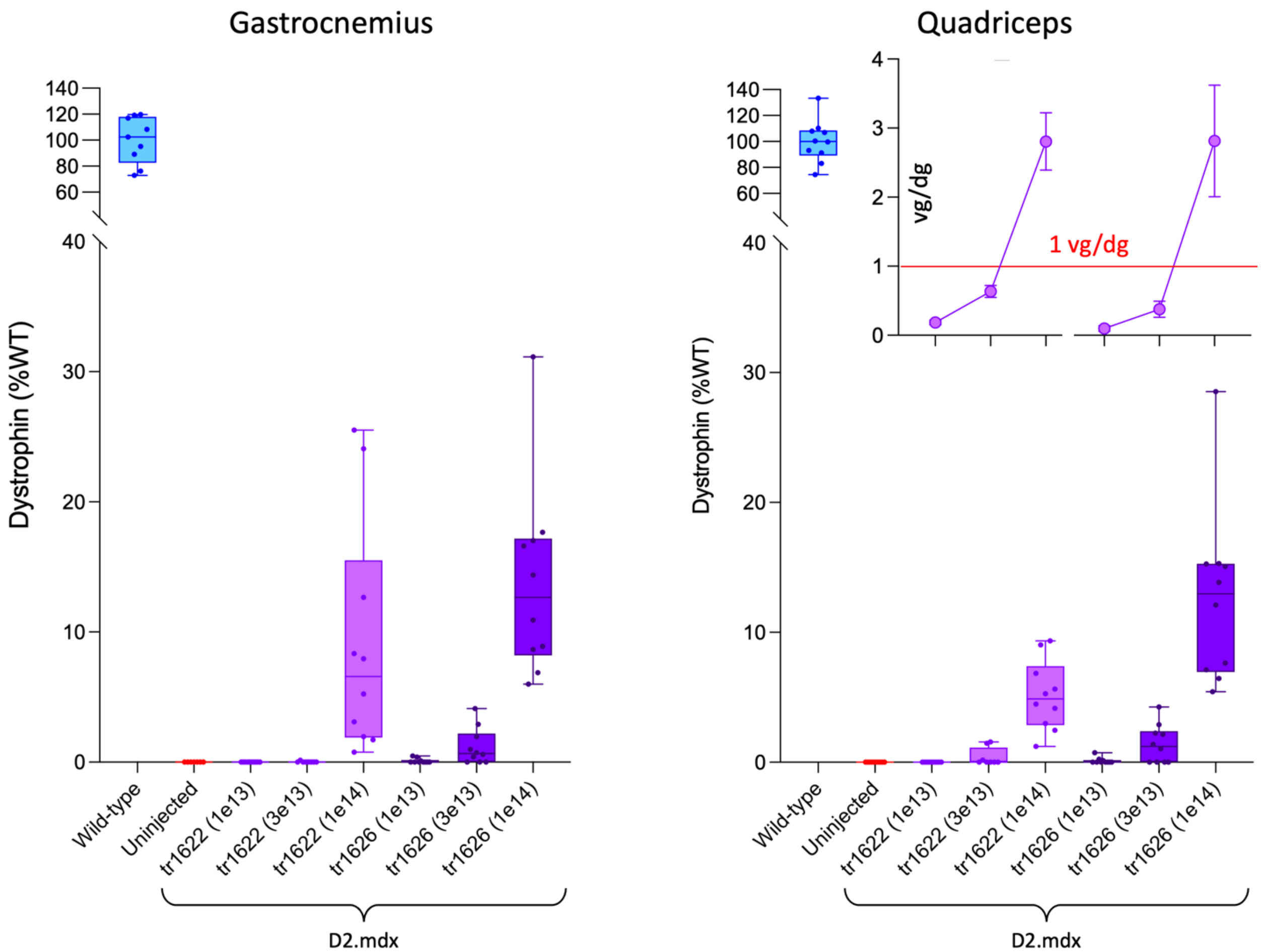

Fig. S26 AAV-tr1622 and AAV-tr1626 treatment results in significant dose-dependent rescue of full-length dystrophin that is limited primarily by AAV biodistribution. Quantification of dystrophin expression in gastrocnemius (**A**) and quadriceps muscles (**B**) following IV injection of AAV-1622 and AAV-1626 at the indicated doses. Inset shows AAV transduction in quadriceps for the indicated vectors at the indicated doses. Full-length dystrophin was measured by capillary western blot. Dystrophin levels in individual animals were normalized to wild type average from the same age.

Table S1. Sequences and ID numbers for all tRNAs screened

ID	Amino Acid	Anticodon	Class	Sequence	Evaluated IM	Evaluated IV
oT_2075	Gln (Q)	TTA		GGCCCCGTGGTGTAATGGTTAGCACTCTGGACTTTAAATCCAGCGATCCGAGTTCAAGTCTCGGTGGAACCT		
tr0159	Gln (Q)	TTA	Parental	GGTTCCATGGTGTAATGGCTAGCACTCTGGACTTTAAATCCAGCGATCCGAGTTCAAATCTCGGTGGGATTT		
tr0907	Gln (Q)	TTA		GGATCCATGGTGTAATGGTTAGCACTCTGGACTTTAAATCCAGCGACCCGAGTTCAAATCTCGGTGGGTTCT		
oT_2589	Gln (Q)	TTA		GGACCCATGGTGTAATGGTTAGCACTCTGGACTTTAAATCCAGCAACCCAAGTTCAAATCTCGGTGGGACCT		
oT_3236	Gln (Q)	TTA		GGACCCATGGTGTAATGGTTAGCACTCTGGACTTTAAATCCAGCAATCCAAGTTCAAATCTCGGTGGGACCC		
tr0165	Gln (Q)	TTA	Parental	GGACCCATGGTGTAATGGTTAGCACTCTGGACTTTAAATCCAGCAATCCAAGTTCAAATCTCGGTGGGACCT		
tr1672	Gln (Q)	TTA		GGTCCCATGGTGTAATGGTTAGCACTCTGGGCTTTAAATCCAGCAATCCGAGTTCGAATCTTGGTGGGATCT		
oT_2022	Gln (Q)	TTA		GGTTCCATGGTGTAATGGTTAGCACTCTGGACTTTAAATCCAGCGATCCGAGTTCAAATCTCGGAGGAACCT		
oT_2585	Gln (Q)	TTA		GGTTCCATGGTGTAATGGCTAGCACTCTGGACTTTAAATCCAGCGACCCGAGTTCAAATCTCGGTGGGATTT		
oT_3430	Gln (Q)	TTA		GGACCCATGGTGTAATGGTTAGCACTCTGGACTTTAACTCCAGCAATCCAAGTTCAAATCTCGGTGGGACCT		
tr1670	Gln (Q)	TTA		GGACCCATGGTGTAATGGTTAGCAGTCTGGACTTTAAATCCAGCAATCCAAGTTCAAATCTCGGTGGGACCT		
oT_2571	Gln (Q)	TTA		GGACCCATGGTGTAATGGTTAGCACTCTGGACTTTAAATCCAGCAACCCAAGTTCGAGTCTCGGTGGGACCT		
oT_2071	Gln (Q)	TTA		GGCCCCATGGTGTGATGGTCAGCACTCTGGACTTTAAATCCAGCGATCCGAGCTCAAATCTCGGTGGGACCT		
oT_2144	Gln (Q)	TTA		GGACCCATGGTGTAATGGTTAGCACTCTGGACTTTAAATCCAGCAATCCAAGTTCGAGTCTCGGTGGGACCT		
oT_3400	Gln (Q)	TTA		GGTTCCATGGTCTAATGGTGAGCACTCTGGACTTTAAATCCAGCGATCCGAGTTCAAATCTCGGTGGGACCT		
oT_3262	Gln (Q)	TTA		GGCTCCATGGTGTAATGGTTAGCACTCTGGACTTTAAATCCAGCGATCCGAGTTCAAATCTCGGTGGGACTT		
tr0867	Gln (Q)	TTA	Parental	GGTTCCATGGTGTAATGGTAAGCACTCTGGACTTTAAATCCAGCCATCTGAGTTCGAGTCTCTGTGGAACCT		
oT_3435	Gln (Q)	TTA		GGCCCCATGGTGTAATGGTTAGCACTCTGGAATTTAAATCCAGCGATCCGAGTTCAAATCTCGGTGGGACCT		
oT_2018	Gln (Q)	TTA		GGTCCCATGGTGTAATGGTTAGCACTCTGGACTTTAAATCCAGCAATCTGAGTTCTAATCTCGGTGGGACCT		
tr1671	Gln (Q)	TTA		GGTTCCATGGTGTAATGGCTAGCACTCTGGACTTTAATTCCAGCGATCCGAGTTCAAATCTCGGTGGGATTT		
oT_2017	Gln (Q)	TTA		CGTCCCATGGTGTAATGGTTAGCACTCTGGGCTTTAAATCCAGCAATCCGAGTTCGAATCTTGGTGGGACCT		
oT_2063	Gln (Q)	TTA		GGCCCCATGGTGTAATGGTTAGCACTCTGGACTTTAAATCCACCGATCCAAGTTCAAATCTTGGTGGGACCT		
oT_3429	Gln (Q)	TTA		GGTCCCATGGTGTAATGGTTAGCACTCTGGGCTTTAAATCCAGCAATCCGAGTTCGAATCTTGGGGGACCT		
oT_3433	Gln (Q)	TTA		GGCCCCATGGTGTAATGGTCAGCACTCTGGACTTTAAGTCCAGCGATCCGAGTTCAAATCTCGGTGGGACCC		
oT_3434	Gln (Q)	TTA		GGACCCATGGTGTAATGGTTAGCACTCTGGACTTTAAACCCAGCAATCCAAGTTCAAATCTCGGTGGGACCT		
oT_2597	Gln (Q)	TTA		GGTCCCATGGTGTAATGGTTAGCACTCTGGGCTTTAAATCCAGCAACCCGAGTTCGAATCTTGGTGGGACCT		
oT_2014	Gln (Q)	TTA		GGTTCCATGGTGTAATGGTGAGCACTCTGGACTTTAAATCCAGCGATCCGAGTTCAAATATCGGTGGGACCT		
oT_2016	Gln (Q)	TTA		GGTTCCATGGTGTAATGGTTAGCACTCTGGACTTTAAATCCAGCGATCCGAGTTCAAATCTCGGTGAAACCT		
oT_2015	Gln (Q)	TTA		GGTTCCCTGGTGTAATGGTTAGCACTCTGGACTTTAAATCCAGCGACCCGAGTTCAAATCTCGGTGGGACCT		
oT_2068	Gln (Q)	TTA		GGCCCCATGGTGTAATGGTTAGCACTCTGGACTTTAAATCCAGCAGTCCGAGTTCAAGTCTCGGTGGGACCT		
oT_3428	Gln (Q)	TTA		GGTTCCATGGTGTAATGGTAAGCACTCTGGACTTTAAATCCAGCGATCCGAGTTCGAGTCTCGGTGGAAACT		
oT_2013	Gln (Q)	TTA		GGTCCCATGGTGTAATGGTTAGCACTCTGGACTTTAAATCCAGCAATCCGAGTTCGAATCTCGGTGGGAGCT		
oT_3432	Gln (Q)	TTA		GGTCCCATGGTGTAATGGTTAGCACTCTGGACTTTAAATGCAGCGATCCGAGTTCAAATCTCGGTGGGACCT		
oT_2012	Gln (Q)	TTA		GGTTCCATGGTGTAATGGTTCGCACTCTGGACTTTAAATCCAGCGACCCGAGTTCAAATCTCGGTGGGACCT		
oT_3295	Gln (Q)	TTA		GGTTCCATGGTGTAATGGTGAGCACTCTGGACTTTAAATCCAGCGATGCGAGTTCAAATCTCGCTGGGACTT		
oT_3219	Gln (Q)	TTA		GGCTCCATGGTGTAATGGTTAGCACTCTGGACTTTAAATCCAGCGATCCGAGTTCAAATCTCGGTGGGACCC		
oT_2615	Gln (Q)	TTA		GGTCCCATGGTGTAGTGGTTAGCACTCTGGGCTTTAAATCCAGCAACCCGAGTTCGAATCTTGGTGGGACCT		
tr0868	Gln (Q)	TTA	Parental	GGTCCCATGGTGTAATGGTTAGCACTCTGGGCTTTAAATCCAGCAATCCGAGTTCGAATCTTGGTGGGACCT		
oT_3297	Gln (Q)	TTA		GGCCCCATGGTGTAATGGTTAACACTCTGGACTTTAAATCCAGCGCTCCGAGTTCAAATCTCGGTGGGACTT		
oT_3242	Gln (Q)	TTA		GGTCCCATGGTGTAATGGTTAGCACTCTGGGCTTTAAATCCAGCAATCCGAGTTCGAATCTTGGTGGGACCC		
oT_3279	Gln (Q)	TTA		GGACCCATGGTGTAATGGTTAGCACTCTGGACTTTAAATCCAGCAATCCAAGTTCAAATCTCGGTGGGACTT		
oT_3296	Gln (Q)	TTA		GGTCCCATGGTGTAATGGTTATCACTCTGGACTTTAAATCCAGAGATCCGAGTTCAAATCTCGGTGGGACTT		
tr1501	Gln (Q)	TTA		GGCTCCATGGTGTAATGGTTAGCACTCTGGACTTTAAATCCAGCGATCCGAGTTCAAATCTCGGTGGGACCT	Yes	
oT_2074	Gln (Q)	TTA		GGCCCCATGGTGTAATGGTCAGCACTCTGGACTTTAAATTCAGCGATCCGAGTTCAAGTCTCGGTGGGACCT		
oT_3298	Gln (Q)	TTA		GGCCCCATGGTGTAATGGTTAGCACCCTGGACTTTAAATCCAGTGATCCGAGTTCAAATCTCGGTGGGACTT		
oT_2524	Gln (Q)	TTA		GGCCCCATGGTGTAATGGTTATCACTCTGGACTTTAAATCCAGCTATCCGAGTTCAAATCTCGGTGGGACCT		
oT_3287	Gln (Q)	TTA		GGTCCCATGGTGTAATGGTTAGCACTCTGGGCTTTAAATCCAGCAATCCGAGTTCGAATCTTGGTGGGACTT		
oT_3277	Gln (Q)	TTA		GGCCCCATGGTGTAATGGTTAGCACTCTGGACTTTAAATCCAGCGATCCGAGTTCAAATCTCGGTGGGACTT		
oT_2149	Gln (Q)	TTA		GGTCCCATGGTGTAATGGTTAGCACTCTGGGCTTTAAATCCAGCAATCCGAGTTCGAGTCTTGGTGGGACCT		
oT_3263	Gln (Q)	TTA		GGCCCCATGGTGTAATGGTCAGCACTCTGGTCTTTAAAACCAGCGATCCGAGTTCAAATCTCGGTGGGACTT		
oT_2554	Gln (Q)	TTA		GGCCCCATGGTGTAATGGTTATCACTCTGGTCTTTAAAACCAGCGACCCGAGTTCAAATCTCGGTGGGACCT		
oT_2537	Gln (Q)	TTA		AGCCCCATGGTGTAATGGTTAGCACTCTGGTCTTTAAAACCAGCGATCCGAGTTCAAATCTCGGTGGGACTT		
oT_3299	Gln (Q)	TTA		GGCCCCATGGTGTAATGGTTAGCACCCTGGACTTTAAATCCAGAGATCCGAGTTCAAATCTCGGTGGGACTT		
oT_2042	Gln (Q)	TTA		GGTTCCATGGTGTAATGGTTAGCACTCTGGTCTTTAAAACCAGCGACCCGAGTTCAAATCTCGGTGGGACCT		
oT_3258	Gln (Q)	TTA		GGTTCCATGGTGTAATGGTAAGCACTCTGGACTTTAAATCCAGCGATCCGAGTTCGAGTCTCGGTGGAGCTT		
oT_2528	Gln (Q)	TTA		GGCCCCATGGTGTAATGGTTATCACTCTGGACTTTAAATCCAGCGACCCGAGTTCAAATCTCGGTGGGACCT		
oT_2550	Gln (Q)	TTA		GGCCCCATGGTGTAATGGTTATCACTCTGGTCTTTAAAACCAGCTATCCGAGTTCAAATCTCGGTGGGACCT		
oT_2562	Gln (Q)	TTA		GGCCCCATGGTGTAATGGTTAGCACACTGGTCTTTAAAACCAGAGATCCGAGTTCAAATCTCGGTGGGACCT		
oT_2552	Gln (Q)	TTA		GGCCCCATGGTGTAATGGTTATCACTCTGGTCTTTAAAACCAGCGTTCCGAGTTCAAATCTCGGTGGGACCT		
oT_3273	Gln (Q)	TTA		GGTTCCATGGTGTAATGGTTAGCACTCTGGACTTTAAATCCAGCGACCCGAGTTCAAATCTCGGTGGGACTT		
oT_2062	Gln (Q)	TTA		GGCCCCATGGTGTAACGGTTAGCACTCTGGACTTTAAATCCGGCGATCTGAGTTCAAATCTCGGTGGGACCT		
oT_3292	Gln (Q)	TTA		GGTTCCATGGTGTAATGGTGAGCACTCTGGACTTTAAATCCAGCGATACGAGTTCGAGTCTCGTTGGAACTT		
oT_2530	Gln (Q)	TTA		GGCCCCATGGTGTAATGGTTAGCACGCTGGACTTTAAATCCAGAGATCCGAGTTCAAATCTCGGTGGGACCT		
oT_3401	Gln (Q)	TTA		GGTTCCATGGTGTAATGGTGAGCACTTTGGACTTTAAATCCAGCGATCCGAGTTCAAATCTCGGTGGGACCT		
oT_2536	Gln (Q)	TTA		GGCCCCATGGTGTAATGGTTAGCACACTGGACTTTAAATCCAGAGATCCGAGTTCAAATCTCGGTGGGACCT		
oT_2594	Gln (Q)	TTA		GGCCCCATGGTGTAATGGTCAGCACTCTGGACTTTAAATCCAGCGACCCGAGTTCAAATCTCGGTGGGACCC		
oT_3424	Gln (Q)	TTA		GGTTCCATGGTGTAAGGGTAAGCACTCTGGACTTTAAATCCAGCGATCCGAGTTCGAGTCTCGGTGGAACCT		
oT_2058	Gln (Q)	TTA		GGTCCCATGGTGTAATGGTTAGCACTCTGGTCTTTAAAACCAGCAATCCGAGTTCGAATCTTGGTGGGACCT		
oT_2511	Gln (Q)	TTA		AGCCCCATGGTGTAATGGTTAGCACTCTGGACTTTAAATCCAGCGATCCGAGTTCAAATCTCGGTGGGACTT		
oT_2526	Gln (Q)	TTA		GGCCCCATGGTGTAATGGTTATCACTCTGGACTTTAAATCCAGCGTTCCGAGTTCAAATCTCGGTGGGACCT		
oT_2543	Gln (Q)	TTA		GGCCCCATGGTGTAATGGTTAGCACTCTGGTCTTTAAAACCAGCGATCCCAGTTCAAATCTGGGTGGGACCT		
oT_2085	Gln (Q)	TTA		GGCCCCATGGTGTAACGGTTAGCACTCTGGTCTTTAAAACCGGCGATCCGAGTTCAAATCTCGGTGGGACCT		
oT_2010	Gln (Q)	TTA		GGTTCTATGGTGTAATGGTTAGCACTCTGGACTTTAAATCCAGCGATCCGAGTTCAAATCTCGGTGGGACCT		
oT_2077	Gln (Q)	TTA		GGCCCCATGGTGTAATGGTAAGCACTCTGGACTTTAAATCCAGCGATCCGAGTTCAAGTCTTGGTGGGACCT		
oT_3405	Gln (Q)	TTA		GGTTCCATGGTGTAAAGGTTAGCACTCTGGACTTTAAATCCAGCGATCCGAGTTCAAATCTCGGTGGAACCT		
tr1504	Gln (Q)	TTA		GGCCCCATGGTGTAATGGTTAGCACTCTGGTCTTTAAAACCAGCGATCCGAGTTCAAATCTCGGTGGGACCC	Yes	
oT_2517	Gln (Q)	TTA		GGCCCCATGGTGTAATGGTTAGCACTCTGGACTTTAAATCCAGCGATCCCAGTTCAAATCTGGGTGGGACCT		
oT_2539	Gln (Q)	TTA		GGCCCCATGGTGTAATGGTTAGCACTCAGGTCTTTAAAACCTGCGATCCGAGTTCAAATCTCGGTGGGACCT		
oT_2505	Gln (Q)	TTA		GGCTCCATGGTGTAATGGTTAGCACTCTGGACTTTAAATCCAGCGATCCGAGTTCGAGTCTCGGTGGGACCT		
oT_3284	Gln (Q)	TTA		GGCCCCATGGTGTAATGGTCAGCACTCTGGACTTTAAATCCAGCGATCCGAGTTCAAATCTCGGTGGGACTC		
oT_2061	Gln (Q)	TTA		GGCCCCATGGTGTAACGGTTAGCACTCTGGACTTTAAATCCGGCGATCCGAGTTCAAATCTCGGTGGGACCT		
oT_3303	Gln (Q)	TTA		GGTTCCATGGTGTAATGGTGAGCACTCTGGTCTTTAAAACCAGCGATCCGAGTTCGAGTCTCGGTGGAACTC		
oT_2555	Gln (Q)	TTA		GGCCCCATGGTGTAATGGTTAGCACGCTGGTCTTTAAAACCAGTGATCCGAGTTCAAATCTCGGTGGGACCT		
tr1502	Gln (Q)	TTA		GGCCCCATGGTGTAATGGTCAGCACTCTGGTCTTTAAAACCAGCGATCCGAGTTCAAATCTCGGTGGGACCC	Yes	

ID	Amino Acid	Anticodon	Class	Sequence	Evaluated IM	Evaluated IV
oT_2045	Gln (Q)	TTA		GGTTCCATGGTGTAATGGTGAGCACTCTGGTCTTTAAAACCAGCGATCCGAGTTCAAATCTCGGTGGGACCT		
oT_2097	Gln (Q)	TTA		GGCCCCATGGTGTAATGGCTAGCACTCTGGTCTTTAAAACCAGCGATCCGAGTTCAAATCTCGGTGGGACCT		
oT_2056	Gln (Q)	TTA		GGTTCCATGGTGTAATGGTTAGCACTCTGGTCTTTAAAACCAGCGATCCGAGTTCAAGTCTCGGTGGAACCT		
oT_2047	Gln (Q)	TTA		GGTCCCATGGTGTAATGGTTAGCACTCTGGTCTTTAAAACCAGCAATCTGAGTTCAAATCTCGGTGGGACCT		
oT_2529	Gln (Q)	TTA		GGCCCCATGGTGTAATGGTTAGCACGCTGGACTTTAAATCCAGTGATCCGAGTTCAAATCTCGGTGGGACCT		
oT_2545	Gln (Q)	TTA		GGCCCCATGGTGTAATGGTTAGCACTCGGGTCTTTAAAACCCGCGATCCGAGTTCAAATCTCGGTGGGACCT		
oT_2094	Gln (Q)	TTA		GGCCCCATGGTGTAATGGTTAGCACTCTGGTCTTTAAAACCAGCGATCTGAGTTCAAATCTCGGTGGGACCT		
tr1506	Gln (Q)	TTA		GGCCCCATGGTGTAATGGTCAGCACTCTGGTCTTTAAAACCAGCGATCCGAGTTCAAATCTCGGTGGGACCT	Yes	
oT_2541	Gln (Q)	TTA		GGCCCCATGGTGTAATGGTTAGCACTCTGGTCTTTAAAACCAGCGATTCGAGTTCAAATCTCGATGGGACCT		
oT_3409	Gln (Q)	TTA		GGTTCCATGGTGTAGTGGTAAGCACTCTGGACTTTAAATCCAGCGATCCGAGTTCGAGTCTCGGTGGAACCT		
oT_2538	Gln (Q)	TTA		GGCCCAATGGTGTAATGGTTAGCACTCTGGTCTTTAAAACCAGCGATCCGAGTTCAAATCTCGGTTGGACCT		
oT_3416	Gln (Q)	TTA		GGTCCCATGGTGTAATGGTTAGCACTCTGGACTTTAAATCCAGCGATCCGAGTTCAAATCTTGGTGGGACCT		
oT_2052	Gln (Q)	TTA		GGTTCCATGGTGTAATGGTAAGCACTCTGGTCTTTAAAACCAGCGATCCGAGTTCGAGTCTCGGTGGAACCT		
oT_2088	Gln (Q)	TTA		GGCCCCATGGTGTAATGGTTAGCACTCTGGTCTTTAAAACCAGCGATCTGAGTTCGAATCTCGGTGGGACCT		
oT_3252	Gln (Q)	TTA		GGTCCCATGGTGTAATGGTTATCACTCTGGACTTTAAATCCAGAGATCCGAGTTCAAATCTCGGTGGGACCC		
oT_2089	Gln (Q)	TTA		GGCCCCATGGTGTAATGGTTAGCACTCTGGTCTTTAAAACCAGTGATCCGAGTTCAAGTCTCGGTGGGACCT		
tr1722	Gln (Q)	TTA		GGCCCCATGGTGTAATGGTCAGCACTCTGGTCTTTAAAACCAGCGATCCGAGTTCGAGTCTCGGTGGGACCC		
oT_2549	Gln (Q)	TTA		GGCCCCATGGTGTAATGGTTATCACTCTGGTCTTTAAAACCAGCGATCCGAGTTCAAATCTCGGTGGGACCT		
oT_2525	Gln (Q)	TTA		GGCCCCATGGTGTAATGGTTATCACTCTGGACTTTAAATCCAGCAATCCGAGTTCAAATCTCGGTGGGACCT		
oT_2556	Gln (Q)	TTA		GGCCCCATGGTGTAATGGTTAGCACGCTGGTCTTTAAAACCAGAGATCCGAGTTCAAATCTCGGTGGGACCT		
oT_3413	Gln (Q)	TTA		GGTTCCATGGTGTAATGGTTAGCACGCTGGACTTTAAATCCAGCGATCCGAGTTCAAATCTCGGTGGAACCT		
tr1505	Gln (Q)	TTA		GGCCCCATGGTGTAATGGTTAGCACTCTGGACTTTAAATCCAGCGATCCGAGTTCAAATCTCGGTGGGACCC	Yes	
oT_2104	Gln (Q)	TTA		GGCCCCATGGTGTAATGGTCAGCACTCTGGTCTTTAAAACCAGCAATCCGAGTTCAAATCTCGGTGGGACCT		
oT_2561	Gln (Q)	TTA		GGCCCCATGGTGTAATGGTTAGCACACTGGTCTTTAAAACCAGCGATCCGAGTTCAAATCTCGGTGGGACCT		
oT_2551	Gln (Q)	TTA		GGCCCCATGGTGTAATGGTTATCACTCTGGTCTTTAAAACCAGCAATCCGAGTTCAAATCTCGGTGGGACCT		
oT_2044	Gln (Q)	TTA		GGTTCCATGGTGTAATGGTTAGCACTCTGGTCTTTAAAACCAGCGATCCGAGTTCAAATCTCGGTGGGACCT		
oT_2054	Gln (Q)	TTA		GGTCCCATGGTGTAATGGTTAGCACTCTGGTCTTTAAAACCAGCAATCCGAGTTCGAATCTCGGTGGGACCT		
oT_3259	Gln (Q)	TTA		GGTTCCATGGTGTAATGGTTAGCACTCTGGACTTTAAATCCAGCGATACGAGTTCAAATCTCGTTGGAACTT		
oT_2479	Gln (Q)	TTA		GGCCCCATGGTGTAATGGTTAGCACTCTGGTCTTTAAAACCAGCGATCCGAGTTCGAGTCTCGGTGGGACCT		
oT_2547	Gln (Q)	TTA		GGCCCCATGGTGTAGTGGTTAGCACTCTGGTCTTTAAAACCAGCGAGCCGAGTTCAAATCTCGGTGGGACCT		
tr0155	Gln (Q)	TTA	Parental	GGTTCCATGGTGTAATGGTTAGCACTCTGGACTTTAAATCCAGCGACCCGAGTTCAAATCTCGGTGGGACCT		
oT_2540	Gln (Q)	TTA		GGCCCCTTGGTGTAATGGTTAGCACTCTGGTCTTTAAAACCAGCGATCCGAGTTCAAATCTCGGAGGGACCT		
oT_2523	Gln (Q)	TTA		GGCCCCATGGTGTAATGGTTATCACTCTGGACTTTAAATCCAGCGATCCGAGTTCAAATCTCGGTGGGACCT		
tr0904	Gln (Q)	TTA		GGCCCCATGGTGTAATGGTTAGCACTCTGGTCTTTAAAACCAGCGATCCGAGTTCAAATCTCGGTGGGACCT	Yes	
oT_3407	Gln (Q)	TTA		GGTTCCATGGTGTAATGGTGAGCACTCTGGACTTTAAATCCAGCTATCCGAGTTCAAATCTCGGTGGGACCT		
oT_3266	Gln (Q)	TTA		GGTTCCATGGTGTAATGGTAAGCACTCTGGACTTTAAATCCAGCGACCCGAGTTCGAGTCTCGGTGGAACTT		
oT_2521	Gln (Q)	TTA		GGCCCCATGGTGTAGTGGTTAGCACTCTGGACTTTAAATCCAGCGAGCCGAGTTCAAATCTCGGTGGGACCT		
oT_2009	Gln (Q)	TTA		GGCCCCATGGTGTAATGGTTAGCACTCTGGACTTTAAATCCAGCGATCCGAGTTCAAATCTCGGTGGGACCG		
oT_2559	Gln (Q)	TTA		GGCCCCATGGTGTAATGGTTAGCACACTGGTCTTTAAAACCAGTGATCCGAGTTCAAATCTCGGTGGGACCT		
oT_3412	Gln (Q)	TTA		GGTTCCGTGGTGTAATGGTTAGCACTCTGGACTTTAAATCCAGCGATCCGAGTTCAAATCTCGGTGGGACCT		
oT_2584	Gln (Q)	TTA		GGTTCCATGGTGTAATGGTGAGCACTCTGGACTTTAAATCCAGCGACCCGAGTTCAAATCTCGGTGGGACCT		
tr0162	Gln (Q)	TTA	Parental	GGTCCCATGGTGTAATGGTTAGCACTCTGGACTTTAAATCCAGCAATCTGAGTTCAAATCTCGGTGGGACCT		
oT_2051	Gln (Q)	TTA		GGTTCCATGGTGTAATGGTGAGCACTCTGGTCTTTAAAACCAGCGATCCGAGTTCGAGTCTCGGTGGAACCT		
oT_3419	Gln (Q)	TTA		GGTCCCATGGTGTAATGGTTAGCACTCTGGACTTTAAATCCATCAATCCGAGTTCGAATCTCGGTGGGACCT		
oT_3293	Gln (Q)	TTA		GGTCCCATGGTGTAATGGTTAGCACTCTGGGCTTTAAACCCAGCGATCCGAGTTCAAATCTCGGTGGGACTT		
oT_2519	Gln (Q)	TTA		GGCCCCATGGTGTAATGGTTAGCACTCGGGACTTTAAATCCCGCGATCCGAGTTCAAATCTCGGTGGGACCT		
oT_2145	Gln (Q)	TTA		GGTTCCATGGTGTAATGGTTAGCACTCTGGACTTTAAATCCGGTAATCCGAGTTCGAGTCTCGGTGGAACCT		
oT_2073	Gln (Q)	TTA		GGCCCCATGGTGTAATGGCTAGCACTCTGGACTTTAAATCCAGCGATCCGAGTTCAAATCTCGGTGGGACCT		
oT_2560	Gln (Q)	TTA		GGCCCCATGGTGTAATGGTTAGCACACTGGTCTTTAAAACCAGGGATCCGAGTTCAAATCTCGGTGGGACCT		
tr0158	Gln (Q)	TTA	Parental	GGTTCCATGGTGTAATGGTGAGCACTCTGGACTTTAAATCCAGCGATCCGAGTTCAAATCTCGGTGGGACCT		
oT_2102	Gln (Q)	TTA		GGCCCCATGGTGTAATGGTGAGCACTCTGGTCTTTAAAACCAGCGATCCGAGTTCAAGTCTCGGTGGGACCT		
oT_3268	Gln (Q)	TTA		GGTTCCATGGTGTAATGGTGAGCACTCTGGACTTTAAATCCAGTGATCCGAGTTCGAGTCTCGGTGGAACTT		
oT_2103	Gln (Q)	TTA		GGCCCCATGGTGTAATGGTCAGCACTCTGGTCTTTAAAACCAGCGATCCGAGTTCAAGTCTCGGTGGGACCT		
tr0522	Gln (Q)	TTA	Parental	GGTTCCATGGTGTAATGGTTAGCACTCTGGACTTTAAATCCGGTAATCCGAGTTCAAATCTCGGTGGAACCT		
oT_2587	Gln (Q)	TTA		GGCCCCATGGTGTAATGGTTAGCACTCTGGACTTTAAATCCAGCGACCCGAGTTCAAATCTCGGTGGGACCT		
oT_2065	Gln (Q)	TTA		GGCCCCATGGTGTAATGGTTAGCACTCTGGACTTTAAATCCAGTGATCCGAGTTCAAGTCTCGGTGGGACCT		
oT_3276	Gln (Q)	TTA		GGTTCCATGGTGTAATGGTGAGCACTCTGGACTTTAAATCCAGCGATCCGAGTTCAAATCTCGGTGGGACTT		
oT_3403	Gln (Q)	TTA		GGTTCCATGGTGTAATGGTTAGCACTCTGGACTTTAAATCCAGCGATCCGAGTTCAACTCTCGGTGGGACCT		
oT_2515	Gln (Q)	TTA		GGCCCCATGGTGTAATGGTTAGCACTCTGGACTTTAAATCCAGCGATTCGAGTTCAAATCTCGATGGGACCT		
oT_2604	Gln (Q)	TTA		GGTCCCATGGTGTAGTGGTTAGCACTCTGGACTTTAAATCCAGCAACCTGAGTTCAAATCTCGGTGGGACCT		
oT_3229	Gln (Q)	TTA		GGTTCCATGGTGTAATGGTTAGCACTCTGGACTTTAAATCCAGCGACCCGAGTTCAAATCTCGGTGGGACCC		
oT_2613	Gln (Q)	TTA		GGTTCCATGGTGTAGTGGTTAGCACTCTGGACTTTAAATCCAGCGACCCGAGTTCAAGTCTCGGTGGAACCT		
tr1503	Gln (Q)	TTA		GGCCCCATGGTGTAATGGTCAGCACTCTGGACTTTAAATCCAGCGATCCGAGTTCAAATCTCGGTGGGACCT	Yes	
oT_3414	Gln (Q)	TTA		GGTCCCATGGTGTAGTGGTTAGCACTCTGGACTTTAAATCCAGCGATCCGAGTTCAAATCTCGGTGGGACCT		
oT_3417	Gln (Q)	TTA		GGTTCTATGGTGTAATGGTTAGCACTCTGGACTTTAAATCCAGCGATCCGAGTTCAAATCTCGGTGGAACCT		
oT_2093	Gln (Q)	TTA		GGCCCCATGGTGTAATGGTTAGCACTCTGGTCTTTAAAACCAGTGATCCGAGTTCAAATCTCGGTGGGACCT		
oT_3420	Gln (Q)	TTA		GGTTCCATGGTGTAATGGTAAGCACTCCGGACTTTAAATCCAGCGATCCGAGTTCGAGTCTCGGTGGAACCT		
oT_3411	Gln (Q)	TTA		GGTCCCATGGTGTAATGGTTAGCACACTGGACTTTAAATCCAGCAATCCGAGTTCGAATCTCGGTGGGACCT		
tr1500	Gln (Q)	TTA		GGTCCCATGGTGTAATGGTTAGCACTCTGGTCTTTAAAACCAGCGATCCGAGTTCAAATCTCGGTGGGACCT	Yes	
oT_2586	Gln (Q)	TTA		GGTCCCATGGTGTAATGGTTAGCACTCTGGACTTTAAATCCAGCAACCTGAGTTCAAATCTCGGTGGGACCT		
oT_2043	Gln (Q)	TTA		GGTTCCATGGTGTAATGGTTAGCACTCTGGTCTTTAAAACCAGCGATCCGAGTTCAAATCTCGGTGGAACCT		
oT_2542	Gln (Q)	TTA		GGCCCCATGGTGTAATGGTTAGCACTCCGGTCTTTAAAACCGGCGATCCGAGTTCAAATCTCGGTGGGACCT		
oT_3291	Gln (Q)	TTA		GGTCCCTTGGTGTAATGGTTAGCACTCTGGACTTTAAATCCAGCAATCCGAGTTCGAATCTCGGAGGGACTT		
oT_2544	Gln (Q)	TTA		GGCGCCATGGTGTAATGGTTAGCACTCTGGTCTTTAAAACCAGCGATCCGAGTTCAAATCTCGGTGGCACCT		
oT_2139	Gln (Q)	TTA		GGTTCCATGGTGTAATGGTTAGCACTCTGGACTTTAAATCCAGCGATCCGAGTTCGAGTCTCGGTGGAACCT		
tr1499	Gln (Q)	TTA		GGTCCCATGGTGTAATGGTCAGCACTCTGGACTTTAAATCCAGCGATCCGAGTTCAAATCTCGGTGGGACCC	Yes	
oT_2140	Gln (Q)	TTA		GGTTCCATGGTGTAATGGTGAGCACTCTGGACTTTAAATCCAGCGATCCGAGTTCGAGTCTCGGTGGGACCT		
oT_3260	Gln (Q)	TTA		GGTTCCGTGGTGTAATGGTTAGCACTCTGGACTTTAAATCCAGCGACCCGAGTTCAAATCTCGGCGGGACTT		
oT_2609	Gln (Q)	TTA		GGTTCCATGGTGTAGTGGTAAGCACTCTGGACTTTAAATCCAGCGACCCGAGTTCGAGTCTCGGTGGAACCT		
oT_2566	Gln (Q)	TTA		GGTTCCATGGTGTAATGGTGAGCACTCTGGACTTTAAATCCAGCGACCCGAGTTCGAGTCTCGGTGGGACCT		
oT_3265	Gln (Q)	TTA		GGTTCCATGGTGTAATGGTGAGCACTCTGGACTTTAAATCCAGCGACCCGAGTTCGAGTCTCGGTGGAACTT		
tr0525	Gln (Q)	TTA	Parental	GGTTCCATGGTGTAATGGTTAGCACTCTGGACTTTAAATCCAGCGATCCGAGTTCAAGTCTCGGTGGAACCT	Yes	
oT_3406	Gln (Q)	TTA		GGTTCCATGGTGTAATGGTTAGCACTCTGGACTTTAAATCCAGCGATCCGAGTTCGAATCTCGGTGGAACCT		
oT_2091	Gln (Q)	TTA		GGCCCCATGGTGTAATGGTTAGCACTCTGGTCTTTAAAACCAGCGATCCGAGTTCAAGTCTCGGTGGGACCT		

ID	Amino Acid	Anticodon	Class	Sequence	Evaluated IM	Evaluated IV
oT_3410	Gln (Q)	TTA		GGCCCCATGGTGTAATGGTTAGCACCCTGGACTTTAAATCCAGCGATCCGAGTTCAAATCTCGGTGGGACCT		
oT_2512	Gln (Q)	TTA		GGCCCAATGGTGTAATGGTTAGCACTCTGGACTTTAAATCCAGCGATCCGAGTTCAAATCTCGGTTGGACCT		
tr1515	Gln (Q)	TTA		GGTTCCATGGTGTAATGGTGAGCACTCTGGACTTTAAATCCAGCGATCCGAGTTCGAGCCTCGGTGGAACCT	Yes	
tr0524	Gln (Q)	TTA	Parental	GGCCCCATGGTGTAATGGTCAGCACTCTGGACTTTAAATCCAGCGATCCGAGTTCAAATCTCGGTGGGACCC	Yes	Yes
oT_3208	Gln (Q)	TTA		GGTCCCATGGTGTAATGGTAAGCACTCTGGTCTTTAAAACCAGCAATCCGAGTTCGAATCTCGGTGGGACCT		
tr1513	Gln (Q)	TTA		GGTCCCATGGTGTAATGGTTAGCACTCTGGACTTTAAATCCAGCAATCTGAGTTCAAATCTCGGTGGGACTT	Yes	
tr0521	Gln (Q)	TTA	Parental	GGTTCCATGGTGTAATGGTAAGCACTCTGGACTTTAAATCCAGCGATCCGAGTTCGAGTCTCGGTGGAACCT		
oT_2067	Gln (Q)	TTA		GGCCCCATGGTGTAATGGTTAGCACTCTGGACTTTAAATCCAGCGATCCGAGTTCAAGTCTCGGTGGGACCT		
oT_2008	Gln (Q)	TTA		GGTTCCATGGTGTAATGGTTAGCACTCTGGACTTTAAATCCAGCGATCCGAGTTCGAATCTCGGTGGGACCT		
tr0156	Gln (Q)	TTA	Parental	GGTTCCATGGTGTAATGGTTAGCACTCTGGACTTTAAATCCAGCGATCCGAGTTCAAATCTCGGTGGAACCT		
oT_2513	Gln (Q)	TTA		GGCCCCATGGTGTAATGGTTAGCACTCAGGACTTTAAATCCTGCGATCCGAGTTCAAATCTCGGTGGGACCT		
oT_2520	Gln (Q)	TTA		GGCCCCATGGTGTAATGGTTAGCACTCTGGGCTTTAAACCCAGCGATCCGAGTTCAAATCTCGGTGGGACCT		
oT_3210	Gln (Q)	TTA		GGTTCCATGGTGTAATGGTGAGCACTCTGGTCTTTAAAACCAGCGACCCGAGTTCGAGTCTCGGTGGAACCT		
oT_2558	Gln (Q)	TTA		GGCCCCATGGTGTAATGGTTAGCACCCTGGTCTTTAAAACCAGAGATCCGAGTTCAAATCTCGGTGGGACCT		
oT_3264	Gln (Q)	TTA		GGTTCCATGGTGTAATGGTGAGCACTCTGGACTTTAAATCCAGCGATCCGAGTTCGAGCCTCGGTGGAACTT		
oT_3218	Gln (Q)	TTA		GGTCCCATGGTGTAATGGTTAGCACTCTGGTCTTTAAAACCAGCGATCCGAGTTCAAATCTCGGTGGGACCC		
oT_3209	Gln (Q)	TTA		GGTTCCATGGTGTAATGGTGAGCACTCTGGTCTTTAAAACCAGCGATCCGAGTTCGAGCCTCGGTGGAACCT		
oT_2548	Gln (Q)	TTA		GGCCCCATGGTGTAGTGGTTAGCACTCTGGTCTTTAAAACCAGCGACCCGAGTTCAAATCTCGGTGGGACCT		
oT_2142	Gln (Q)	TTA		GGTCCCATGGTGTAATGGTTAGCACTCTGGACTTTAAATCCAGCAATCTGAGTTCGAGTCTCGGTGGGACCT		
tr1792	Gln (Q)	TTA		GGTTCCATGGTGTAATGGTGAGCACTCTGGACTTTAAATCCAGCGATCCGAGTTCGAGTCTCGGTGGAACTT		
oT_2533	Gln (Q)	TTA		GGCCCCATGGTGTAATGGTTAGCACACTGGACTTTAAATCCAGTGATCCGAGTTCAAATCTCGGTGGGACCT		
oT_3251	Gln (Q)	TTA		GGTTCCATGGTGTAATGGTGAGCACTCTGGACTTTAAATCCAGCGATGCGAGTTCAAATCTCGCTGGGACCC		
oT_2137	Gln (Q)	TTA		GGTCCCATGGTGTAATGGTTAGCACTCTGGACTTTAAATCCAGCGATCCGAGTTCGAGTCTCGGTGGGACCT		
tr1621	Gln (Q)	TTA		GGCCCCATGGTGTAATGGTTAGCACTCTGGACTTTAAATCCAGCGATCCGAGTTCGAGTCTCGGTGGGACCT	Yes	
tr0523	Gln (Q)	TTA	Parental	GGTCCCATGGTGTAATGGTTAGCACTCTGGACTTTAAATCCAGCAATCCGAGTTCGAATCTCGGTGGGACCT	Yes	
tr1787	Gln (Q)	TTA		GGCCCCATGGTGTAATGGTTAGCACTCTGGACTTTAAATCCAGCGATCCGAGTTCGAGTCTCGGTGGGACCC		
oT_2612	Gln (Q)	TTA		GGCCCCATGGTGTAGTGGTCAGCACTCTGGACTTTAAATCCAGCGACCCGAGTTCAAATCTCGGTGGGACCC		
tr0906	Gln (Q)	TTA		GGTTCCGTGGTGTAATGGTTAGCACTCTGGACTTTAAATCCAGCGACCCGAGTTCAAATCTCGGCGGGACCT	Yes	
oT_2553	Gln (Q)	TTA		GGCCCCATGGTGTAATGGTTAACACTCTGGTCTTTAAAACCAGCGCTCCGAGTTCAAATCTCGGTGGGACCT		
tr1523	Gln (Q)	TTA		GGTTCCATGGTGTAATGGTGAGCACTCTGGACTTTAAATCCAGTGATCCGAGTTCGAGTCTCGGTGGAACCT	Yes	
oT_2146	Gln (Q)	TTA		GGTCCCATGGTGTAATGGTTAGCACTCTGGACTTTAAATCCAGCAATCCGAGTTCGAGTCTCGGTGGGACCT		
oT_3220	Gln (Q)	TTA		GGTTCCATGGTGTAATGGTGAGCACTCTGGACTTTAAATCCAGCGATCCGAGTTCGAGCCTCGGTGGAACCC		
tr0903	Gln (Q)	TTA		GGTTCCATGGTGTAATGGTAAGCACTCTGGACTTTAAATCCAGCGATCCGAGTTCGAGTCTCGGTGGAGCCT	Yes	
tr0157	Gln (Q)	TTA	Parental	GGTTCCATGGTGTAATGGTTAGCACTCTGGACTTTAAATCCAGCGATCCGAGTTCAAATCTCGGTGGGACCT	Yes	
oT_2080	Gln (Q)	TTA		GGCCCCATGGTGTAATGGTCAGCACTCTGGACTTTAAATCCAGCAATCCGAGTTCAAATCTCGGTGGGACCT		
oT_3202	Gln (Q)	TTA		GGTTCCATGGTGTAATGGTTAGCACTCTGGACTTTAAATCCAGCGATACGAGTTCGAGTCTCGTTGGAACCT		
oT_2534	Gln (Q)	TTA		GGCCCCATGGTGTAATGGTTAGCACACTGGACTTTAAATCCAGGGATCCGAGTTCAAATCTCGGTGGGACCT		
oT_2602	Gln (Q)	TTA		GGTTCCATGGTGTAGTGGTGAGCACTCTGGACTTTAAATCCAGCGACCCGAGTTCAAATCTCGGTGGGACCT		
oT_2078	Gln (Q)	TTA		GGCCCCATGGTGTAATGGTGAGCACTCTGGACTTTAAATCCAGCGATCCGAGTTCAAGTCTCGGTGGGACCT		
oT_2611	Gln (Q)	TTA		GGTCCCATGGTGTAGTGGTTAGCACTCTGGACTTTAAATCCAGCAACCCGAGTTCGAATCTCGGTGGGACCT		
oT_2581	Gln (Q)	TTA		GGTCCCATGGTGTAATGGTTAGCACTCTGGACTTTAAATCCAGCGACCCGAGTTCAAATCTCGGTGGGACCT		
oT_3261	Gln (Q)	TTA		GGTCCCATGGTGTAATGGTTAGCACTCTGGTCTTTAAAACCAGCGATCCGAGTTCAAATCTCGGTGGGACTT		
tr0163	Gln (Q)	TTA	Parental	GGCCCCATGGTGTAATGGTTAGCACTCTGGACTTTAAATCCAGCGATCCGAGTTCAAATCTCGGTGGGACCT	Yes	
tr1725	Gln (Q)	TTA		GGTTCCATGGTGTAATGGTGAGCACTCTGGTCTTTAAAACCAGCGATCCGAGTTCGAGTCTCGGTGGAACCC		
oT_2138	Gln (Q)	TTA		GGTTCCATGGTGTAATGGTTAGCACTCTGGACTTTAAATCCAGCGACCCGAGTTCGAGTCTCGGTGGGACCT		
oT_2608	Gln (Q)	TTA		GGTTCCATGGTGTAGTGGTGAGCACTCTGGACTTTAAATCCAGCGACCCGAGTTCGAGTCTCGGTGGAACCT		
oT_2535	Gln (Q)	TTA		GGCCCCATGGTGTAATGGTTAGCACACTGGACTTTAAATCCAGCGATCCGAGTTCAAATCTCGGTGGGACCT		
oT_2064	Gln (Q)	TTA		GGCCCCATGGTGTAATGGTTAGCACTCTGGACTTTAAATCCAGCGATCTGAGTTCGAATCTCGGTGGGACCT		
tr1791	Gln (Q)	TTA		GGTTCCATGGTGTAATGGTGAGCACTCTGGACTTTAAATCCAGTGATCCGAGTTCGAGTCTCGGTGGAACCC		
oT_3271	Gln (Q)	TTA		GGCCCCATGGTGTAATGGTTAGCACTCTGGACTTTAAATCCAGCGATCCGAGTTCGAGTCTCGGTGGGACTT		
oT_2599	Gln (Q)	TTA		GGTTCCATGGTGTAGTGGTTAGCACTCTGGACTTTAAATCCAGCGACCCGAGTTCAAATCTCGGTGGGACCT		
tr1811	Gln (Q)	TTA		GGTTCCGTGGTGTAATGGTTAGCACTCTGGACTTTAAATCCAGCGACCCGAGTTCAAATCTCGGCGGGACCC		
tr1622	Gln (Q)	TTA		GGCCCCATGGTGTAATGGTCAGCACTCTGGACTTTAAATCCAGCGATCCGAGTTCGAGTCTCGGTGGGACCC	Yes	Yes
oT_2582	Gln (Q)	TTA		GGTTCCATGGTGTAATGGTTAGCACTCTGGACTTTAAATCCAGCGACCCGAGTTCAAATCTCGGTGGAACCT		
oT_3415	Gln (Q)	TTA		GGTTCTATGGTGTAATGGTAAGCACTCTGGACTTTAAATCCAGCGATCCGAGTTCGAGTCTCGGTGGAACCT		
oT_2514	Gln (Q)	TTA		GGCCCCTTGGTGTAATGGTTAGCACTCTGGACTTTAAATCCAGCGATCCGAGTTCAAATCTCGGAGGGACCT		
tr1726	Gln (Q)	TTA		GGTCCCATGGTGTAATGGTTAGCACTCTGGACTTTAAATCCAGCAATCTGAGTTCAAATCTCGGTGGGACTC		
oT_2564	Gln (Q)	TTA		GGTTCCATGGTGTAATGGTTAGCACTCTGGACTTTAAATCCAGCGACCCGAGTTCGAGTCTCGGTGGAACCT		
oT_2600	Gln (Q)	TTA		GGTTCCATGGTGTAGTGGTTAGCACTCTGGACTTTAAATCCAGCGACCCGAGTTCAAATCTCGGTGGAACCT		
oT_3213	Gln (Q)	TTA		GGTTCCATGGTGTAATGGTGAGCACTCTGGTCTTTAAAACCAGTGATCCGAGTTCGAGTCTCGGTGGAACCT		
oT_3285	Gln (Q)	TTA		GGTTCCATGGTGTAATGGTTAGCACTCTGGACTTTAAATCCAGCGATCCGAGTTCAAGTCTCGGTGGAACTT		
oT_2557	Gln (Q)	TTA		GGCCCCATGGTGTAATGGTTAGCACCCTGGTCTTTAAAACCAGTGATCCGAGTTCAAATCTCGGTGGGACCT		
oT_2522	Gln (Q)	TTA		GGCCCCATGGTGTAGTGGTTAGCACTCTGGACTTTAAATCCAGCGACCCGAGTTCAAATCTCGGTGGGACCT		
oT_2070	Gln (Q)	TTA		GGCCCCATGGTGTAATGGTTAGCACTCTGGACTTTAAATCCAGCGATCTGAGTTCAAATCTCGGTGGGACCT		
oT_3211	Gln (Q)	TTA		GGTTCCATGGTGTAATGGTAAGCACTCTGGTCTTTAAAACCAGCGACCCGAGTTCGAGTCTCGGTGGAACCT		
oT_2507	Gln (Q)	TTA		GGCCCCATGGTGTAATGGTCAGCACTCTGGACTTTAAATCCAGCGATCCGAGTTCGAGTCTCGGTGGGACCT		
tr1805	Gln (Q)	TTA		GGTTCCATGGTGTAATGGTAAGCACTCTGGACTTTAAATCCAGCGATCCGAGTTCGAGTCTCGGTGGAGCCC		
tr0002	Gln (Q)	TTA	Yes	GGTCCCATGGTGTAATGGTTAGCACTCTGGACTTTAAATCCAGCGATCCGAGTTCAAATCTCGGTGGGACCT		
oT_2516	Gln (Q)	TTA		GGCCCCATGGTGTAATGGTTAGCACTCCGGACTTTAAATCCGGCGATCCGAGTTCAAATCTCGGTGGGACCT		
oT_3198	Gln (Q)	TTA		GGTCCCATGGTGTAATGGTTAGCACTCTGGACTTTAAATCCAGTAATCCGAGTTCGAGTCTCGGTGGGACCT		
tr0520	Gln (Q)	TTA	Yes	GGTTCCATGGTGTAATGGTGAGCACTCTGGACTTTAAATCCAGCGATCCGAGTTCGAGTCTCGGTGGAACCT	Yes	
tr1521	Gln (Q)	TTA		GGTCCCATGGTGTAATGGTTAGCACTCTGGACTTTAAATCCAGTAATCCGAGTTCGAATCTCGGTGGGACCT	Yes	
oT_3422	Gln (Q)	TTA		GGTCCCATGGTGTAATGGTTAGCACTCTGGACTTTAAATCCAGTAATCTGAGTTCAAATCTCGGTGGGACCT		
oT_2518	Gln (Q)	TTA		GGCGCCATGGTGTAATGGTTAGCACTCTGGACTTTAAATCCAGCGATCCGAGTTCAAATCTCGGTGGCACCT		
oT_3421	Gln (Q)	TTA		GGTTCCATGGTGTAATGGTAAGCACTCTGGACTTTAAATCCAGCGATTCGAGTTCGAGTCTCGGTGGAACCT		
tr1806	Gln (Q)	TTA		GGTTCCATGGTGTAATGGTAAGCACTCTGGACTTTAAATCCAGCGACCCGAGTTCGAGTCTCGGTGGAACCC		
tr1799	Gln (Q)	TTA		GGTCCCATGGTGTAATGGTAAGCACTCTGGACTTTAAATCCAGCAATCCGAGTTCGAATCTCGGTGGGACCC		
tr0905	Gln (Q)	TTA		GGTTCCATGGTGTAATGGTTAGCACTCTGGACTTTAAATCCAGCGATACGAGTTCAAATCTCGTTGGAACCT	Yes	
tr1809	Gln (Q)	TTA		GGTTCCATGGTGTAATGGTTAGCACTCTGGACTTTAAATCCAGCGATCCGAGTTCAAATCTCGGTGGGACCC		
oT_3200	Gln (Q)	TTA		GGTCCCATGGTGTAATGGTAAGCACTCTGGACTTTAAATCCAGCAATCCGAGTTCGAGTCTCGGTGGGACCT		
tr1820	Gln (Q)	TTA		GGTCCCATGGTGTAGTGGTTAGCACTCTGGACTTTAAATCCAGCGACCCGAGTTCAAATCTCGGTGGGACCT		
tr1812	Gln (Q)	TTA		GGTTCCATGGTGTAATGGTTAGCACTCTGGACTTTAAATCCAGCGATCCGAGTTCAAATCTCGGTGGAACCC		
oT_3216	Gln (Q)	TTA		GGTTCCATGGTGTAATGGTTAGCACTCTGGACTTTAAATCCAGCGATACGAGTTCAAATCTCGTTGGAACCC		
tr1789	Gln (Q)	TTA		GGCCCCATGGTGTAATGGTTAGCACCCTGGACTTTAAATCCAGTGATCCGAGTTCAAATCTCGGTGGGACCC		

ID	Amino Acid	Anticodon	Class	Sequence	Evaluated IM	Evaluated IV
tr1519	Gln (Q)	TTA		GGTTCCATGGTGTAATGGTAAGCACTCTGGACTTTAAATCCAGCGACCCGAGTTCGAGTCTCGGTGGAACCT	Yes	
tr1790	Gln (Q)	TTA		GGCCCCATGGTGTAATGGTTAGCACCCTGGACTTTAAATCCAGAGATCCGAGTTCAAATCTCGGTGGGACCC		
tr1511	Gln (Q)	TTA		GGTTCCATGGTGTAATGGTGAGCACTCTGGACTTTAAATCCAGCGATCCGAGTTCGAGTCTCGGTGGAACCC	Yes	
oT_2079	Gln (Q)	TTA		GGCCCCATGGTGTAATGGTCAGCACTCTGGACTTTAAATCCAGCGATCCGAGTTCAAGTCTCGGTGGGACCT		
tr1813	Gln (Q)	TTA		GGTTCCATGGTGTAATGGTTAGCACTCTGGACTTTAAATCCAGCGATCCGAGTTCAAATCTCGGTGGAACTT		
tr1807	Gln (Q)	TTA		AGTTCCATGGTGTAATGGTAAGCACTCTGGACTTTAAATCCAGCGATCCGAGTTCGAGTCTCGGTGGAACTC		
tr1531	Gln (Q)	TTA		GGTCCCATGGTGTAATGGTAAGCACTCTGGACTTTAAATCCAGCAATCCGAGTTCGAATCTCGGTGGGACCT	Yes	
tr1814	Gln (Q)	TTA		GGTTCCATGGTGTAATGGTGAGCACTCTGGACTTTAAATCCAGCGATCCGAGTTCAAATCTCGGTGGGACCC		
oT_2069	Gln (Q)	TTA		GGCCCCATGGTGTAATGGTTAGCACTCTGGACTTTAAATCCAGTGATCCGAGTTCAAATCTCGGTGGGACCT		
tr1785	Gln (Q)	TTA		GGTCCCATGGTGTAATGGTTAGCACTCTGGACTTTAAATCCAGCAATCTGAGTTCAAATCTCGGTGGGACCC		
oT_3399	Gln (Q)	TTA		GGTCCCATGGTGTAATGGTTAGCACTCTGGACTTTAAATCCAGCGATCCGAGTTCAAACCTCGGTGGGACCT		
tr1796	Gln (Q)	TTA		GGTCCCATGGTGTAATGGTTAGCACTCTGGACTTTAAATCCAGCAACCCGAGTTCGAGTCTCGGTGGGACCT		
tr1788	Gln (Q)	TTA		GGCCCCATGGTGTAATGGTTAACACTCTGGACTTTAAATCCAGCGCTCCGAGTTCAAATCTCGGTGGGACCC		
tr1712	Gln (Q)	TTA		GGCCCCATGGTGTAATGGTTAGCACCCTGGACTTTAAATCCAGAGATCCGAGTTCAAATCTCGGTGGGACCT		
oT_2569	Gln (Q)	TTA		GGCCCCATGGTGTAATGGTTAGCACTCTGGACTTTAAATCCAGCGACCCGAGTTCGAGTCTCGGTGGGACCT		
tr1710	Gln (Q)	TTA		GGCCCCATGGTGTAATGGTTAACACTCTGGACTTTAAATCCAGCGCTCCGAGTTCAAATCTCGGTGGGACCT		
oT_2568	Gln (Q)	TTA		GGTCCCATGGTGTAATGGTTAGCACTCTGGACTTTAAATCCAGCAACCTGAGTTCGAGTCTCGGTGGGACCT		
tr1711	Gln (Q)	TTA		GGCCCCATGGTGTAATGGTTAGCACCCTGGACTTTAAATCCAGTGATCCGAGTTCAAATCTCGGTGGGACCT		
tr1817	Gln (Q)	TTA		GGTCCCATGGTGTAATGGTTAGCACTCTGGACTTTAAATCCTGCGATCCGAGTTCAAATCTCGGTGGGACTT		
oT_2504	Gln (Q)	TTA		GGTCCCATGGTGTAATGGTTAGCACTCTGGTCTTTAAAACCAGCGATCCGAGTTCGAGTCTCGGTGGGACCT		
tr1819	Gln (Q)	TTA		GGTTCCATGGTGTAATGGTTAGCACTCTGGACTTTAAATCCAGCGACCCGAGTTCAAGTCTCGGTGGAACCT		
tr1723	Gln (Q)	TTA		GGTCCCATGGTGTAATGGTCAGCACTCTGGACTTTAAATCCAGCGATCCGAGTTCGAGTCTCGGTGGGACCC		
tr1804	Gln (Q)	TTA		GGTCCCATGGTGTAATGGTTAGCACTCTGGACTTTAAATCCAGCCATCCGAGTTCGAATCTCGGTGGGACTT		
tr1808	Gln (Q)	TTA		GGTTCCATGGTGTAATGGTAAGCACTCTGGACTTTAAATCCAGCGATCCGAGTTCGAGTCTCGGTGGAACTT		
oT_3425	Gln (Q)	TTA		GGTCCCCTGGTGTAATGGTTAGCACTCTGGACTTTAAATCCAGCGATCCGAGTTCAAATCTCGGTGGGACCT		
tr1802	Gln (Q)	TTA		GGTCCCATGGTGTAATGGTAAGCACTCTGGACTTTAAATCCAGCAATCCGAGTTCGAATCTCGGTGGGACTT		
oT_2563	Gln (Q)	TTA		GGTCCCATGGTGTAATGGTTAGCACTCTGGACTTTAAATCCAGCGACCCGAGTTCGAGTCTCGGTGGGACCT		
tr1797	Gln (Q)	TTA		GGTCCCATGGTGTAATGGTTAGCACTCTGGTCTTTAAAACCAGTAATCCGAGTTCGAATCTCGGTGGGACCT		
tr1777	Gln (Q)	TTA		GGTCCCATGGTGTAATGGTTAGCACTCTGGACTTTAAATCCAGCAATCCGAGTTCGAATCTCGGTGGGACCC		
tr1517	Gln (Q)	TTA		GGTTCCATGGTGTAATGGTGAGCACTCTGGACTTTAAATCCAGCGACCCGAGTTCGAGTCTCGGTGGAACCT	Yes	
tr1795	Gln (Q)	TTA		GGTTCCATGGTGTAATGGTTAGCACTCTGGACTTTAAATCCAGCGATCCGAGTTCAAGTCTCGGTGGAACCC		
tr1801	Gln (Q)	TTA		GGTCCCATGGTGTAATGGTTAGCACTCTGGACTTTAAATCCAGCAACCCGAGTTCGAATCTCGGTGGGACTT		
oT_2576	Gln (Q)	TTA		GGCCCCATGGTGTAATGGTCAGCACTCTGGACTTTAAATCCAGCGACCCGAGTTCGAGTCTCGGTGGGACCC		
tr1778	Gln (Q)	TTA		GGTTCCATGGTGTAATGGTGAGCACTCTGGACTTTAAATCCAGCGACCCGAGTTCGAGTCTCGGTGGAACCC		
tr1702	Gln (Q)	TTA		GGTCCCATGGTGTAATGGTTAGCACTCTGGACTTTAAATCCAGCCATCCGAGTTCGAATCTCGGTGGGACCT		
tr1627	Gln (Q)	TTA		GGCGCCATGGTGTAATGGTGAGCACTCTGGACTTTAAATCCAGCGATCCGAGTTCGAGTCTCGGTGGCGCCT	Yes	
tr1794	Gln (Q)	TTA		GGTCCCATGGTGTAATGGTCAGCACTCTGGACTTTAAATCCAGCGATCCGAGTTCGAGTCTCGGTGGGACTC		
tr1773	Gln (Q)	TTA		GGTTCCATGGTGTAATGGTAAGCACTCTGGACTTTAAATCCAGCGATCCGAGTTCGAGTCTCGGTGGAACCC		
tr1798	Gln (Q)	TTA		GGTCCCATGGTGTAATGGTTAGCACTCTGGTCTTTAAAACCAGCAACCCGAGTTCGAATCTCGGTGGGACCT		
tr1774	Gln (Q)	TTA		GGTCCCATGGTGTAATGGTTAGCACTCTGGACTTTAAATCCAGTAATCCGAGTTCGAATCTCGGTGGGACCC		
tr1783	Gln (Q)	TTA		GGTCCCTTGGTGTAATGGTTAGCACTCTGGACTTTAAATCCAGCAATCCGAGTTCGAATCTCGGAGGGACCC		
tr1775	Gln (Q)	TTA		GGTCCCATGGTGTAATGGTTAGCACTCTGGACTTTAAATCCAGCAATCTGAGTTCGAGTCTCGGTGGGACTC		
tr1701	Gln (Q)	TTA		GGTCCCATGGTGTAATGGTTAGCACTCTGGACTTTAAATCCTGCGATCCGAGTTCAAATCTCGGTGGGACCT		
tr1800	Gln (Q)	TTA		GGTCCCATGGTGTAATGGTTAGCACTCTGGACTTTAAATCCAGCCATCCGAGTTCGAATCTCGGTGGGACCC		
tr1771	Gln (Q)	TTA		GGTCCCATGGTGTAATGGTTAGCACTCTGGACTTTAAATCCAGCAACCCGAGTTCGAATCTCGGTGGGACCC		
tr1816	Gln (Q)	TTA		GGTCCCATGGTGTAATGGTTAGCACTCTGGGCTTTAAACCCAGCGATCCGAGTTCAAATCTCGGTGGGACCC		
tr1815	Gln (Q)	TTA		GGTCCCATGGTGTAATGGTTAGCACTCTGGACTTTAAATCCTGCGATCCGAGTTCAAATCTCGGTGGGACCC		
tr1727	Gln (Q)	TTA		GGTCCCATGGTGTAATGGTTAGCACTCTGGACTTTAAATCCAGCAATCTGAGTTCGAGTCTCGGTGGGACTT		
tr1782	Gln (Q)	TTA		GGTTCCATGGTGTAATGGTGAGCACTCTGGACTTTAAATCCAGCGATACGAGTTCGAGTCTCGTTGGAACCC		
tr1525	Gln (Q)	TTA		GGTCCCATGGTGTAATGGTTAGCACTCTGGACTTTAAATCCAGCAACCCGAGTTCGAATCTCGGTGGGACCT	Yes	
tr1803	Gln (Q)	TTA		GGTCCCATGGTGTAATGGTTAGCACTCTGGACTTTAAATCCAGCAATCCGAGTTCGAATCTCGGTGGGACTT		
tr1772	Gln (Q)	TTA		GGTCCCATGGTGTAATGGTTAGCACTCTGGACTTTAAATCCAGCGATCCGAGTTCAAATCTCGGTGGGACCC		
tr1779	Gln (Q)	TTA		GGTCCCATGGTGTAATGGTTAGCACTCTGGACTTTAAATCCAGTAATCCGAGTTCGAATCTCGGTGGGACTT		
tr1776	Gln (Q)	TTA		GGTCCCATGGTGTAATGGTTAGCACTCTGGACTTTAAATCCAGCGATCCGAGTTCAAATCTCGGTGGGACTT		
tr1781	Gln (Q)	TTA		GGTCCCATGGTGTAATGGTTAGCACTCTGGACTTTAAATCCAGCGACCCGAGTTCGAATCTCGGTGGGACCC		
tr1780	Gln (Q)	TTA		GGTCCCATGGTGTAATGGTTAGCACTCTGGACTTTAAATCCAGCGACCCGAGTTCGAATCTCGGTGGGACTT		
tr1626	Gln (Q)	TTA		GGACCCATGGTGTAATGGTGAGCACTCTGGACTTTAAATCCAGCGATCCGAGTTCGAGTCTCGGTGGGTCCT	Yes	Yes
tr0164	Gln (Q)	TTA	Parental	GGTCTCATGGTGTAATGGTTAGCACACTGGACTTTAAGTCCAGCAATCCGAGTTCGAGTCTTGGTGAGACCA		
tr1233	Ser (S)	TTA	Parental	GTAGTCGTGGCCGAGTGGTTAAGGCGATGGACTTTAAATCCATTGGGGTCTCCCCGCGCAGGTTCGAATCCTGCCGACTACG	Yes	
tr0538	Ser (S)	TTA	Parental	GTAGTCGTGGCCGAGTGGTtAAGGCGATGGACTttaAATCCATTGGGGTTTCCCCGCGCAGGTTCGAATCCTGCCGACTACG	Yes	
tr0211	Tyr (Y)	TTA	Parental	CCTTCGATAGCTCAGCTGGTAGAGCGGAGGACTttaGATCCTTAGGtCGCTGGTTCGATTCCGGCTCGAAGGA	Yes	
tr0212	Tyr (Y)	TTA	Parental	CCTTCGATAGCTCAGTTGGTAGAGCGGAGGACTttaGATCCTTAGGtCGCTGGTTCGAATCCGGCTCGAAGGA	Yes	

Controls

ID	Amino Acid	Anticodon	Class	Sequence	Evaluated IM	Evaluated IV
tr0874	Gln (Q)	CTA	Control	GGTCCCATGGTGTAATGGTTAGCACTCTGGGCTCTAAATCCAGCAATCCGAGTTCGAATCTTGGTGGGACCT		
tr0869	Gln (Q)	CTA	Control	GGTTCCATGGTGTAATGGTTAGCACTCTGGACTCTAAATCCAGCGATCCGAGTTCAAGTCTCGGTGGAACCT		
tr0190	Gln (Q)	CTA	Control	GGTTCCATGGTGTAATGGTTAGCACTCTGGACTCTAAATCCAGCGATCCGAGTTCAAATCTCGGTGGAACCT		
tr0870	Gln (Q)	CTA	Control	GGTTCCATGGTGTAATGGTGAGCACTCTGGACTCTAAATCCAGCGATCCGAGTTCGAGTCTCGGTGGAACCT		
tr0873	Gln (Q)	CTA	Control	GGTTCCATGGTGTAATGGTAAGCACTCTGGACTCTAAATCCAGCCATCTGAGTTCGAGTCTCTGTGGAACCT		
tr0191	Gln (Q)	CTA	Control	GGTTCCATGGTGTAATGGTTAGCACTCTGGACTCTAAATCCAGCGATCCGAGTTCAAATCTCGGTGGGACCT		
tr0196	Gln (Q)	CTA	Control	GGTCCCATGGTGTAATGGTTAGCACTCTGGACTCTAAATCCAGCGATCCGAGTTCAAATCTCGGTGGGACCT		
tr0606	Gln (Q)	CTA	Control	GGTCCCATGGTGTAATGGTTAGCACTCTGGACTCTAAATCCAGCAATCCGAGTTCGAATCTCGGTGGGACCT		
tr0198	Gln (Q)	CTA	Control	GGCCCCATGGTGTAATGGTTAGCACTCTGGACTCTAAATCCAGCGATCCGAGTTCAAATCTCGGTGGGACCT		
tr0199	Gln (Q)	CTA	Control	GGTCTCATGGTGTAATGGTTAGCACACTGGACTCTAAGTCCAGCAATCCGAGTTCGAGTCTTGGTGAGACCA		
tr0189	Gln (Q)	CTA	Control	GGTTCCATGGTGTAATGGTTAGCACTCTGGACTCTAAATCCAGCGACCCGAGTTCAAATCTCGGTGGGACCT		
tr0216	Gln (Q)	CTA	Control	GGCCCCATGGTGTAATGGTCAGCACTCTGGACTCTAAATCCAGCGATCCGAGTTCAAATCTCGGTGGGACCC		
tr0871	Gln (Q)	CTA	Control	GGTTCCATGGTGTAATGGTAAGCACTCTGGACTCTAAATCCAGCGATCCGAGTTCGAGTCTCGGTGGAACCT		
tr0193	Gln (Q)	CTA	Control	GGTTCCATGGTGTAATGGCTAGCACTCTGGACTCTAAATCCAGCGATCCGAGTTCAAATCTCGGTGGGATTT		
tr0197	Gln (Q)	CTA	Control	GGTCCCATGGTGTAATGGTTAGCACTCTGGACTCTAAATCCAGCAATCTGAGTTCAAATCTCGGTGGGACCT		
tr0872	Gln (Q)	CTA	Control	GGTTCCATGGTGTAATGGTTAGCACTCTGGACTCTAAATCCGGTAATCCGAGTTCAAATCTCGGTGGAACCT		
tr0200	Gln (Q)	CTA	Control	GGACCCATGGTGTAATGGTTAGCACTCTGGACTCTAAATCCAGCAATCCAAGTTCAAATCTCGGTGGGACCT		
tr0192	Gln (Q)	CTA	Control	GGTTCCATGGTGTAATGGTGAGCACTCTGGACTCTAAATCCAGCGATCCGAGTTCAAATCTCGGTGGGACCT		
tr1731	Gln (Q)	CTA	Control	GGTCCCATGGTGTAATGGTTATCACTCTGGACTCTAAATCCAGAGATCCGAGTTCAAATCTCGGTGGGACCT		

ID	Amino Acid	Anticodon	Class	Sequence	Evaluated IM	Evaluated IV
tr1717	Gln (Q)	CTA	Control	GGTTCCATGGTGTAATGGTGAGCACTCTGGACTCTAAATCCAGCGATACGAGTTCGAGTCTCGTTGGAACCT		
tr1721	Gln (Q)	CTA	Control	GGTTCCATGGTGTAATGGTGAGCACTCTGGACTCTAAATCCAGCGATGCGAGTTCAAATCTCGCTGGGACCT		
tr1716	Gln (Q)	CTA	Control	GGTCCCTTGGTGTAATGGTTAGCACTCTGGACTCTAAATCCAGCAATCCGAGTTCGAATCTCGGAGGGACCT		
tr1720	Gln (Q)	CTA	Control	GGTCCCATGGTGTAATGGTTAGCACACTGGACTCTAAATCCAGGAATCTGAGTTCAAATCTCGGTGGGACCT		
tr1719	Gln (Q)	CTA	Control	AGTTCCATGGTGTAATGGTAAGCACTCTGGACTCTAAATCCAGCGATCCGAGTTCGAGTCTCGGTGGAACTT		
tr1718	Gln (Q)	CTA	Control	GGTCCCATGGTGTAATGGTTAGCACTCTGGGCTCTAAACCCAGCGATCCGAGTTCAAATCTCGGTGGGACCT		
tr1715	Gln (Q)	CTA	Control	GGTCCCATGGTGTAATGGTTAGCACTCTGGACTCTAAATCCAGCGACCCGAGTTCGAATCTCGGTGGGACCT		
tr0860	Gln (Q)	CTG	Control	GGTTCCATGGTGTAATGGTTAGCACTCTGGACTCTGAATCCAGCGACCCGAGTTCAAATCTCGGTGGGACCT		
tr0856	Gln (Q)	CTG	Control	GGCCCCATGGTGTAATGGTCAGCACTCTGGACTCTGAATCCAGCGATCCGAGTTCAAATCTCGGTGGGACCC		
tr0854	Gln (Q)	CTG	Control	GGTTCCATGGTGTAATGGTAAGCACTCTGGACTCTGAATCCAGCGATCCGAGTTCGAGTCTCGGTGGAACCT		
tr0863	Gln (Q)	CTG	Control	GGTTCCATGGTGTAATGGCTAGCACTCTGGACTCTGAATCCAGCGATCCGAGTTCAAATCTCGGTGGGATTT		
tr0853	Gln (Q)	CTG	Control	GGTTCCATGGTGTAATGGTGAGCACTCTGGACTCTGAATCCAGCGATCCGAGTTCGAGTCTCGGTGGAACCT		
tr0862	Gln (Q)	CTG	Control	GGTTCCATGGTGTAATGGTGAGCACTCTGGACTCTGAATCCAGCGATCCGAGTTCAAATCTCGGTGGGACCT		
tr0852	Gln (Q)	CTG	Control	GGTTCCATGGTGTAATGGTTAGCACTCTGGACTCTGAATCCAGCGATCCGAGTTCAAGTCTCGGTGGAACCT		
tr0855	Gln (Q)	CTG	Control	GGTTCCATGGTGTAATGGTTAGCACTCTGGACTCTGAATCCGGTAATCCGAGTTCAAATCTCGGTGGAACCT		
tr0857	Gln (Q)	CTG	Control	GGTTCCATGGTGTAATGGTAAGCACTCTGGACTCTGAATCCAGCCATCTGAGTTCGAGTCTCTGTGGAACCT		
tr0861	Gln (Q)	CTG	Control	GGTTCCATGGTGTAATGGTTAGCACTCTGGACTCTGAATCCAGCGATCCGAGTTCAAATCTCGGTGGGACCT		
tr0851	Gln (Q)	CTG	Control	GGTTCCATGGTGTAATGGTTAGCACTCTGGACTCTGAATCCAGCGATCCGAGTTCAAATCTCGGTGGAACCT		
tr0545	Gln (Q)	TTG	Control	GGCCCCATGGTGTAATGGTTAGCACTCTGGACTTTGAATCCAGCGATCCGAGTTCAAATCTCGGTGGGACCT		
tr0859	Gln (Q)	TTG	Control	GGTCCCATGGTGTAATGGTTAGCACTCTGGGCTTTGAATCCAGCAATCCGAGTTCGAATCTTGGTGGGACCT		
tr0864	Gln (Q)	TTG	Control	GGTCCCATGGTGTAATGGTTAGCACTCTGGACTTTGAATCCAGCAATCTGAGTTCAAATCTCGGTGGGACCT		
tr0271	Gln (Q)	TTG	Control	GGTCCCATGGTGTAATGGTTAGCACTCTGGACTTTGAATCCAGCAATCCGAGTTCGAATCTCGGTGGGACCT		
tr0858	Gln (Q)	TTG	Control	GGTCCCATGGTGTAATGGTTAGCACTCTGGACTTTGAATCCAGCGATCCGAGTTCAAATCTCGGTGGGACCT		
tr0866	Gln (Q)	TTG	Control	GGACCCATGGTGTAATGGTTAGCACTCTGGACTTTGAATCCAGCAATCCAAGTTCAAATCTCGGTGGGACCT		
tr0865	Gln (Q)	TTG	Control	GGTCTCATGGTGTAATGGTTAGCACACTGGACTTTGAGTCCAGCAATCCGAGTTCGAGTCTTGGTGAGACCA		

Cohort	Mouse	Aldoa	Mybpc1	Myl1	Ttn (1)	Ttn (2)
WT	mD_0159					
WT	mD_0161					
WT	mD_0158					
WT	mD_0164					
WT	mD_0157					
WT	mD_0163					
WT	mD_0155					
WT	mD_0156					
WT	mD_0160					
WT	mD_0162					
mdx	mD_0182					
mdx	mD_0183					
mdx	mD_0177				38,852	
mdx	mD_0175					
mdx	mD_0176					
mdx	mD_0184					
mdx	mD_0181					
mdx	mD_0178					
mdx	mD_0179					
mdx	mD_0180					
1e14 vg/kg	mD_0194	1,417,350	126,310		125,320	18,970
1e14 vg/kg	mD_0193	1,910,440	172,252		144,898	19,117
1e14 vg/kg	mD_0189	1,087,150	101,875	122,011	110,707	12,015
1e14 vg/kg	mD_0187	956,382	122,196		84,243	18,164
1e14 vg/kg	mD_0190	2,313,590	158,547	332,214	181,607	41,761
1e14 vg/kg	mD_0185	2,139,980	112,186	284,599	124,348	32,771
1e14 vg/kg	mD_0188	1,128,010	114,488	130,740	57,187	13,810
1e14 vg/kg	mD_0192	1,519,950	149,703		133,981	21,631
1e14 vg/kg	mD_0191	1,760,330	181,097	261,024	136,682	15,018
1e14 vg/kg	mD_0186	1,274,990	154,794		108,002	16,926
2e14 vg/dg	mD_0199	3,470,770	314,065	449,146	227,799	33,913
2e14 vg/dg	mD_0201	4,266,980	542,494	533,059	292,312	35,577
2e14 vg/dg	mD_0204	2,451,040	403,821	302,057	181,110	35,171
2e14 vg/dg	mD_0200	3,183,420	333,164	386,705	224,466	49,050
2e14 vg/dg	mD_0203	4,649,750	605,249	602,829	282,235	72,413
2e14 vg/dg	mD_0195	2,060,900	271,159		145,077	40,899
2e14 vg/dg	mD_0198	2,718,300	411,196	383,787	231,946	40,537
2e14 vg/dg	mD_0196	3,006,310	231,615	368,102	190,261	38,370
2e14 vg/dg	mD_0197	2,269,820	181,408	221,340	158,203	16,657
2e14 vg/dg	mD_0202	2,817,310	389,654	483,597	210,906	9,306
4e14 vg/dg	mD_0274	5,214,910	617,159	696,794	334,066	44,488
4e14 vg/dg	mD_0273	3,418,520	437,912	369,112	212,086	45,723
4e14 vg/dg	mD_0270	3,515,520	355,940	409,218	226,072	36,592
4e14 vg/dg	mD_0272	4,902,910	685,150	383,746	284,150	35,239
4e14 vg/dg	mD_0271	3,691,310	463,680	463,097	209,065	53,103

Protein	Peptide
Aldoa	YTPSGQSGAAASESLFISNHAYQPELN
Mybpc1	WAVLLQAPPAR
Myl1	HIMSVQTEFSSTQC(UniMod:4)WGR
Ttn (peptide 1)	GPFTLDAHS
Ttn (peptide 2)	SMQEGQGQER

Table S2 . All 3'-UTR extended peptides detected by LC-MS/MS Peptide intensity for all 3'-UTR extended peptides detected by quantitative LC-MS/MS in quadriceps muscles of mice. Each row shows data from one animal; each column shows data for the indicated peptide.

REFERENCES

1. T. Markati, M. Oskoui, M. A. Farrar, T. Duong, N. Goemans, L. Servais, Emerging therapies for Duchenne muscular dystrophy. *Lancet Neurol.* **21**, 814–829 (2022).

2. L. Chung Liang, N. Sulaiman, M. D. Yazid, A decade of progress in gene targeted therapeutic strategies in Duchenne muscular dystrophy: A systematic review. *Front. Bioeng. Biotechnol.* **10**, 833833 (2022).

3. S. Szwec, Z. Kapłucha, J. S. Chamberlain, P. Konieczny, Dystrophin- and utrophin-based therapeutic approaches for treatment of Duchenne muscular dystrophy: A comparative review. *BioDrugs* **38**, 95–119 (2024).

4. S. Takeda, P. R. Clemens, E. P. Hoffman, Exon-skipping in Duchenne muscular dystrophy. *J. Neuromuscul. Dis.* **8**, S343–S358 (2021).

5. B. J. Byrne, K. M. Flanigan, S. E. Matesanz, R. S. Finkel, M. A. Waldrop, E. S. D'Ambrosio, N. E. Johnson, B. K. Smith, C. Bönnemann, S. Carrig, J. W. Rossano, B. Greenberg, L. Lalaguna, E. Lara-Pezzi, S. Subramony, M. Corti, C. Mercado-Rodriguez, C. Leon-Astudillo, R. Ahrens-Nicklas, D. Bharucha-Goebel, G. Gao, D. J. Gessler, W.-L. Hwu, Y.-H. Chien, N.-C. Lee, S. L. Boye, S. E. Boye, L. A. George, Current clinical applications of AAV-mediated gene therapy. *Mol. Ther.* **33**, 2479–2516 (2025).

6. M. Mort, D. Ivanov, D. N. Cooper, N. A. Chuzhanova, A meta-analysis of nonsense mutations causing human genetic disease. *Hum. Mutat.* **29**, 1037–1047 (2008).

7. A. M. Tabish, V. Azzimato, A. Alexiadis, B. Buyandelger, R. Knöll, Genetic epidemiology of titin-truncating variants in the etiology of dilated cardiomyopathy. *Biophys. Rev.* **9**, 207–223 (2017).

8. J. M. Goodenbour, T. Pan, Diversity of tRNA genes in eukaryotes. *Nucleic Acids Res.* **34**, 6137–6146 (2006).

9. J. Coller, Z. Ignatova, tRNA therapeutics for genetic diseases. *Nat. Rev. Drug Discov.* **23**, 108–125 (2024).

10. J. Wang, Y. Zhang, C. A. Mendonca, O. Yukselen, K. Muneeruddin, L. Ren, J. Liang, C. Zhou, J. Xie, J. Li, Z. Jiang, A. Kucukural, S. A. Shaffer, G. Gao, D. Wang, AAV-delivered suppressor tRNA overcomes a nonsense mutation in mice. *Nature* **604**, 343–348 (2022).

11. S. E. Pierce, S. Erwood, K. Oye, M. An, N. Krasnow, E. Zhang, A. Raguram, D. Seelig, M. J. Osborn, D. R. Liu, Prime editing-installed suppressor tRNAs for disease-agnostic genome editing. *Nature* **648**, 191–202 (2025).

12. K. Mangkalaphiban, F. He, R. Ganesan, C. Wu, R. Baker, A. Jacobson, Transcriptome-wide investigation of stop codon readthrough in Saccharomyces cerevisiae. *PLOS Genet.* **17**, e1009538 (2021).

13. W. D. Coley, L. Bogdanik, M. C. Vila, Q. Yu, J. H. Van Der Meulen, S. Rayavarapu, J. S. Novak, M. Nearing, J. L. Quinn, A. Saunders, C. Dolan, W. Andrews, C. Lammert, A. Austin, T. A. Partridge, G. A. Cox, C. Lutz, K. Nagaraju, Effect of genetic background on the dystrophic phenotype in mdx mice. *Hum. Mol. Genet.* **25**, 130–145 (2016).

14. J. Ji, E. Lefebvre, J. Laporte, Comparative in vivo characterization of newly discovered myotropic adeno-associated vectors. *Skelet. Muscle* **14**, 9 (2024).

15. M. Tabebordbar, K. A. Lagerborg, A. Stanton, E. M. King, S. Ye, L. Tellez, A. Krunnfusz, S. Tavakoli, J. J. Widrick, K. A. Messemer, E. C. Troiano, B. Moghadaszadeh, B. L. Peacker, K. A. Leacock, N. Horwitz, A. H. Beggs, A. J. Wagers, P. C. Sabeti, Directed evolution of a family of AAV capsid variants enabling potent muscle-directed gene delivery across species. *Cell* **184**, 4919–4938.e22 (2021).

16. C. Beekman, A. A. Janson, A. Baghat, J. C. van Deutekom, N. A. Datson, Use of capillary Western immunoassay (Wes) for quantification of dystrophin levels in skeletal muscle of healthy controls and individuals with Becker and Duchenne muscular dystrophy. *PLOS ONE* **13**, e0195850 (2018).

17. K. Bushby, R. Finkel, D. J. Birnkrant, L. E. Case, P. R. Clemens, L. Cripe, A. Kaul, K. Kinnett, C. McDonald, S. Pandya, J. Poysky, F. Shapiro, J. Tomezsko, C. Constantin, DMD Care Considerations Working Group, Diagnosis and management of Duchenne muscular dystrophy, part 1: Diagnosis, and pharmacological and psychosocial management. *Lancet Neurol.* **9**, 77–93 (2010).

18. M. van Putten, K. Putker, M. Overzier, W. A. Adamzek, S. Pasteuning-Vuhman, J. J. Plomp, A. Aartsma-Rus, Natural disease history of the *D2-mdx* mouse model for Duchenne muscular dystrophy. *FASEB J.* **33**, 8110–8124 (2019).

19. S. Albers, B. Beckert, M. C. Matthies, C. S. Mandava, R. Schuster, C. Seuring, M. Riedner, S. Sanyal, A. E. Torda, D. N. Wilson, Z. Ignatova, Repurposing tRNAs for nonsense suppression. *Nat. Commun.* **12**, 3850 (2021).

20. M. Dabrowski, Z. Bukowy-Bieryllo, E. Zietkiewicz, Translational readthrough potential of natural termination codons in eucaryotes—The impact of RNA sequence. *RNA Biol.* **12**, 950–958 (2015).

21. S. J. Carr, R. P. Zahedi, H. Lochmüller, A. Roos, Mass spectrometry-based protein analysis to unravel the tissue pathophysiology in Duchenne muscular dystrophy. *Proteomics Clin. Appl.* **12**, 1700071 (2018).

22. T. L. E. van Westering, H. J. Johansson, B. Hanson, A. M. L. Coenen-Stass, Y. Lomonosova, J. Tanihata, N. Motohashi, T. Yokota, S. Takeda, J. Lehtiö, M. J. A. Wood, S. El Andaloussi, Y. Aoki, T. C. Roberts, Mutation-independent proteomic signatures of pathological progression in murine models of Duchenne muscular dystrophy. *Mol. Cell. Proteomics MCP* **19**, 2047–2068 (2020).

23. M. V. Petkova, A. Stantzou, A. Morin, O. Petrova, S. Morales-Gonzalez, F. Seifert, J. Bellec-Dyevre, T. Manoliu, A. Goyenvalle, L. Garcia, I. Richard, C. Laplace-Builhé, M. Schuelke, H. Amthor, Live-imaging of revertant and therapeutically restored dystrophin in the $Dmd^{EGFP\text{-}mdx}$ mouse model for Duchenne muscular dystrophy. *Neuropathol. Appl. Neurobiol.* **46**, 602–614 (2020).

24. R. Klietsch, J. M. Ervasti, W. Arnold, K. P. Campbell, A. O. Jorgensen, Dystrophin-glycoprotein complex and laminin colocalize to the sarcolemma and transverse tubules of cardiac muscle. *Circ. Res.* **72**, 349–360 (1993).

25. D. J. Blake, A. Weir, S. E. Newey, K. E. Davies, Function and genetics of dystrophin and dystrophin-related proteins in muscle. *Physiol. Rev.* **82**, 291–329 (2002).

26. L. A. Megeney, B. Kablar, R. L. Perry, C. Ying, L. May, M. A. Rudnicki, Severe cardiomyopathy in mice lacking dystrophin and MyoD. *Proc. Natl. Acad. Sci. U.S.A.* **96**, 220–225 (1999).

27. H. M. Hayes, J. Angerosa, A. T. Piers, J. D. White, J. Koleff, M. Thurgood, J. Moody, M. M. Cheung, S. Pepe, Preserved left ventricular function despite myocardial fibrosis and myopathy in the dystrophin-deficient D2.B10-Dmd^{mdx} /J mouse. *Oxid. Med. Cell. Longev.* **2022**, 5362115 (2022).

28. D. W. Hammers, C. C. Hart, M. K. Matheny, L. A. Wright, M. Armellini, E. R. Barton, H. L. Sweeney, The D2.mdx mouse as a preclinical model of the skeletal muscle pathology associated with Duchenne muscular dystrophy. *Sci. Rep.* **10**, 14070 (2020).

29. D. Duan, Lethal immunotoxicity in high-dose systemic AAV therapy. *Mol. Ther.* **31**, 3123–3126 (2023).

30. K. Chwalenia, V.-Y. Feng, N. Hemmer, H. J. Friedrichsen, I. Vorobieva, M. J. A. Wood, T. C. Roberts, AAV microdystrophin gene replacement therapy for Duchenne muscular dystrophy: Progress and prospects. *Gene Ther.* **32**, 447–461 (2025).

31. Y. Zhang, H. Li, Y.-L. Min, E. Sanchez-Ortiz, J. Huang, A. A. Mireault, J. M. Shelton, J. Kim, P. P. A. Mammen, R. Bassel-Duby, E. N. Olson, Enhanced CRISPR-Cas9 correction of Duchenne muscular dystrophy in mice by a self-complementary AAV delivery system. *Sci. Adv.* **6**, eaay6812 (2020).

32. A. M. Domitrovich, G. R. Kunkel, Multiple, dispersed human U6 small nuclear RNA genes with varied transcriptional efficiencies. *Nucleic Acids Res.* **31**, 2344–2352 (2003).

33. U. Etxaniz, I. Marks, T. Albin, M. Diaz, R. Bhardwaj, A. Anderson, O. Tyaglo, T. Hoang, M. A. Missinato, K. Svensson, B. Badillo, P. R. Kovach, L. Leung, M. Cochran, H. W. Kwon, M. N. Ahad Shah, R. Maruyama, T. Yokota, V. R. Doppalapudi, B. Darimont, H. S. Younis, W. M. Flanagan, A. A. Levin, H. Huang, G. Karamanlidis, AOC 1044 induces exon 44 skipping and restores dystrophin protein in preclinical models of Duchenne muscular dystrophy. *Nucleic Acids Res.* **53**, gkaf241 (2025).

34. W. Cui, D. Zhao, J. Jiang, F. Tang, C. Zhang, C. Duan, tRNA modifications and modifying enzymes in disease, the potential therapeutic targets. *Int. J. Biol. Sci.* **19**, 1146–1162 (2023).

35. S. K. Schultz, U. Kothe, RNA modifying enzymes shape tRNA biogenesis and function. *J. Biol. Chem.* **300**, 107488 (2024).

36. C. L. Bladen, D. Salgado, S. Monges, M. E. Foncuberta, K. Kekou, K. Kosma, H. Dawkins, L. Lamont, A. J. Roy, T. Chamova, V. Guergueltcheva, S. Chan, L. Korngut, C. Campbell, Y. Dai, J. Wang, N. Barišić, P. Brabec, J. Lahdetie, M. C. Walter, O. Schreiber-Katz, V. Karcagi, M. Garami, V. Viswanathan, F. Bayat, F. Buccella, E. Kimura, Z. Koeks, J. C. van den Bergen, M. Rodrigues, R. Roxburgh, A. Lusakowska, A. Kostera-Pruszczyk, J. Zimowski, R. Santos, E. Neagu, S. Artemieva, V. M. Rasic, D. Vojinovic, M. Posada, C. Bloetzer, P.-Y. Jeannet, F. Joncourt, J. Díaz-Manera, E. Gallardo, A. A. Karaduman, H. Topaloğlu, R. El Sherif, A. Stringer, A. V. Shatillo, A. S. Martin, H. L. Peay, M. I. Bellgard, J. Kirschner, K. M. Flanigan, V. Straub, K. Bushby, J. Verschuuren, A. Aartsma-Rus, C. Béroud, H. Lochmüller, The TREAT-NMD DMD Global Database: Analysis of more than 7,000 Duchenne muscular dystrophy mutations. *Hum. Mutat.* **36**, 395–402 (2015).

37. J. R. Mendell, N. Khan, N. Sha, H. Eliopoulos, C. M. McDonald, N. Goemans, E. Mercuri, L. P. Lowes, L. N. Alfano, Eteplirsen Study Group, Comparison of long-term ambulatory function in patients with Duchenne muscular dystrophy treated with eteplirsen and matched natural history controls. *J. Neuromuscul. Dis.* **8**, 469–479 (2021).

38. L. Servais, E. Mercuri, V. Straub, M. Guglieri, A. M. Seferian, M. Scoto, D. Leone, E. Koenig, N. Khan, A. Dugar, X. Wang, B. Han, D. Wang, F. Muntoni, SKIP-NMD Study Group, Long-term safety and efficacy data of golodirsen in ambulatory patients with Duchenne muscular

dystrophy amenable to exon 53 skipping: A first-in-human, multicenter, two-part, open-label, phase 1/2 trial. *Nucleic Acid Ther.* **32**, 29–39 (2022).

39. B. Roy, W. J. Friesen, Y. Tomizawa, J. D. Leszyk, J. Zhuo, B. Johnson, J. Dakka, C. R. Trotta, X. Xue, V. Mutyam, K. M. Keeling, J. A. Mobley, S. M. Rowe, D. M. Bedwell, E. M. Welch, A. Jacobson, Ataluren stimulates ribosomal selection of near-cognate tRNAs to promote nonsense suppression. *Proc. Natl. Acad. Sci. U.S.A.* **113**, 12508–12513 (2016).

40. O. Pinkard, S. McFarland, T. Sweet, J. Coller, Quantitative tRNA-sequencing uncovers metazoan tissue-specific tRNA regulation. *Nat. Commun.* **11**, 4104 (2020).

41. T. R. Helliwell, N. T. Man, G. E. Morris, K. E. Davies, The dystrophin-related protein, utrophin, is expressed on the sarcolemma of regenerating human skeletal muscle fibres in dystrophies and inflammatory myopathies. *Neuromuscul. Disord.* **2**, 177–184 (1992).

42. P. Soblechero-Martín, A. López-Martínez, L. de la Puente-Ovejero, A. Vallejo-Illarramendi, V. Arechavala-Gomeza, Utrophin modulator drugs as potential therapies for Duchenne and Becker muscular dystrophies. *Neuropathol. Appl. Neurobiol.* **47**, 711–723 (2021).

43. C. Croissant, R. Carmeille, C. Brévart, A. Bouter, Annexins and membrane repair dysfunctions in muscular dystrophies. *Int. J. Mol. Sci.* **22**, 5276 (2021).

44. F. Cynthia Martin, M. Hiller, P. Spitali, S. Oonk, H. Dalebout, M. Palmblad, A. Chaouch, M. Guglieri, V. Straub, H. Lochmüller, E. H. Niks, J. J. G. M. Verschuuren, A. Aartsma-Rus, A. M. Deelder, Y. E. M. van der Burgt, P. A. C. ‘t Hoen, Fibronectin is a serum biomarker for Duchenne muscular dystrophy. *Proteomics Clin. Appl.* **8**, 269–278 (2014).

45. P. Dowling, S. Gargan, S. Murphy, M. Zweyer, H. Sabir, D. Swandulla, K. Ohlendieck, The dystrophin node as integrator of cytoskeletal organization, lateral force transmission, fiber stability and cellular signaling in skeletal muscle. *Proteomes* **9**, 9 (2021).

46. S. Mareedu, E. D. Million, D. Duan, G. J. Babu, Abnormal calcium handling in Duchenne muscular dystrophy: Mechanisms and potential therapies. *Front. Physiol.* **12**, 647010 (2021).

47. M. L. Law, H. Cohen, A. A. Martin, A. B. B. Angulski, J. M. Metzger, Dysregulation of calcium handling in Duchenne muscular dystrophy-associated dilated cardiomyopathy: Mechanisms and experimental therapeutic strategies. *J. Clin. Med.* **9**, 520 (2020).
